# Diazaphosphinanes as hydride, hydrogen atom, proton or electron donors under transition-metal-free conditions: thermodynamics, kinetics, and synthetic applications†

**DOI:** 10.1039/c9sc05883d

**Published:** 2020-03-05

**Authors:** Jingjing Zhang, Jin-Dong Yang, Jin-Pei Cheng

**Affiliations:** Center of Basic Molecular Science, Department of Chemistry, Tsinghua University Beijing 100084 China jdyang@mail.tsinghua.edu.cn jinpei_cheng@mail.tsinghua.edu.cn; State Key Laboratory of Elemento-Organic Chemistry, College of Chemistry, Nankai University Tianjin 300071 China

## Abstract

Exploration of new hydrogen donors is in large demand in hydrogenation chemistry. Herein, we developed a new 1,3,2-diazaphosphinane **1a**, which can serve as a hydride, hydrogen atom or proton donor without transition-metal mediation. The thermodynamics and kinetics of these three pathways of **1a**, together with those of its analog **1b**, were investigated in acetonitrile. It is noteworthy that, the reduction potentials (*E*_red_) of the phosphenium cations **1a-[P]+** and **1b-[P]+** are extremely low, being −1.94 and −2.39 V (*vs.* Fc^+/0^), respectively, enabling corresponding phosphinyl radicals to function as neutral super-electron-donors. Kinetic studies revealed an extraordinarily large kinetic isotope effect KIE(**1a**) of 31.3 for the hydrogen atom transfer from **1a** to the 2,4,6-tri-(*tert*-butyl)-phenoxyl radical, implying a tunneling effect. Furthermore, successful applications of these diverse P–H bond energetic parameters in organic syntheses were exemplified, shedding light on more exploitations of these versatile and powerful diazaphosphinane reagents in organic chemistry.

## Abstract 2

A new 1,3,2-diazaphosphinane, serving as a formal hydride, hydrogen-atom or proton donor without transition-metal mediation was exploited thermodynamically and kinetically. And, its promising potentials in versatile syntheses have been demonstrated.

## Graphical abstract

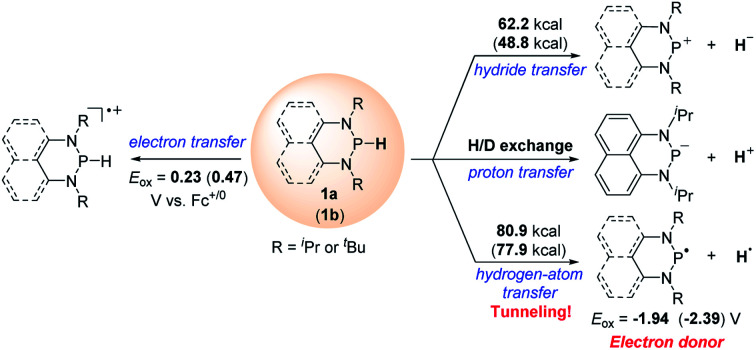

## Introduction

**Scheme 1:**
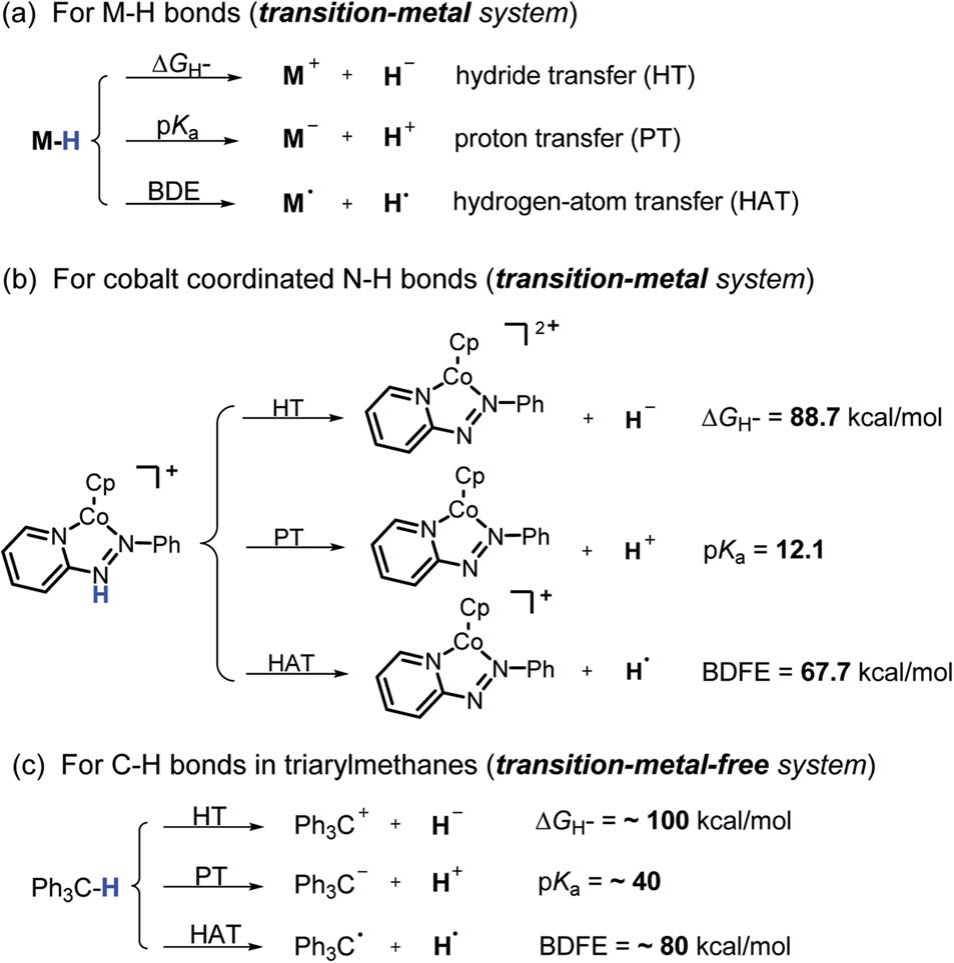
Possible pathways of hydrogen transfers for transition-metal and transition-metal-free systems as well as corresponding thermodynamic driving forces in acetonitrile.

Hydrogen transfer plays a very important role in chemical science and related fields. This process occurs through the cleavage of the targeted R–H bond in three possible pathways (Scheme 1), *i.e.*, hydride (H^−^),^1^ hydrogen atom (H˙)^2^ and proton (H^+^)^3^ transfers, which, from a thermodynamic aspect, depends on the R–H bond strength and the nature of the atom bound to the transferred hydrogen atom. Accordingly, knowledge of the relevant thermodynamics of hydricity Δ*G*_H^−^_,^1*b*,*c*,4^ bond-dissociation free-energy, BDFE^5^ and acidity, p*K*_a_^3*c*,6^ as well as the kinetics^7^ relevant to these processes would facilitate rational exploitations of new transformations. In recent years, considerable attention has been paid to the development of new hydrogen sources with versatile hydrogen donor reactivities, with particular interests in transition-metal hydrides M–H,^1*c*^ such as hydrogenase enzyme analogs^8^ and hydrogenation catalysts,^9^ and main-group organic hydrides X–H,^1*b*^ such as nicotinamide coenzyme models^10^ and Hantzsch esters,^11^ and so on.

It is well known that metal hydrides (M–H) could serve as H^−^, H˙ and H^+^ donors (Scheme 1a).^1*c*,12^ Their diverse hydrogen reactivities originate from the readily modulated polarity of M–H bonds through electronic communication between the metal centers and coordinated ligands. Compared to an M–H system, the X–H hydride manifested some advantages in mild reaction conditions, good functional-group compatibility, easy modification, *etc.* Integration of these three dissociation possibilities into one X–H covalent bond is a substantial challenge, due to the great disparity in the electronegativity of the X and H fragments. Nevertheless, Waymouth *et al.* very recently reported an excellent new example of such a X–H bond where the N–H bond of the phenylazopyridine ligand in the Co(i) complex can serve as either a H^−^, H˙ or a H^+^ donor (Scheme 1b).^13^ It is noted, however, that the diverse reactivity of this N–H bond may require latent electronic communication between the azopyridine ligand and cobalt atom within the five-membered ring, so it may be viewed as a quasi-M–H system.

For a true transition-metal-free system, till now only triarylmethane analogs, where there exist steric as well as resonance stabilization effects on the incipient radical or charges, were reported to have α-C–H bond-cleavage energies determined for all three pathways (Scheme 1c).^14^ However, their poor hydrogen donability and severe steric hindrance make these dissociation processes not very useful for synthesis. This, in turn, stimulated our interest to find more useful transition-metal-free X–H systems that promise both the desired energetic measurement and new synthetic applications.

Recently, diazaphospholenium hydrides (P–H) have attracted substantial research interest due to their superior hydricity endowed by the unique diazaphospholene skeleton.^15^ Many diazaphospholenes were developed and successfully used in various hydridic reductions.^16^ However, the superior hydricity resulted in their other promising applications, such as serving as hydrogen atom and proton donors, as well as the precursors of strong electron donors, being greatly overlooked. Considering the similar electronegativity of the hydrogen (*χ*^AR^ = 2.20) and phosphorus (*χ*^AR^ = 2.06) atoms,^15*b*^ we reckoned that the P–H bond, beside being a good hydride donor,^17^ may have the potential to release proton and hydrogen atoms^18^ as well, by fine-tuning the electrical properties of the P fragment. And indeed luckily enough, this was realized in the present work.

**Scheme 2:**
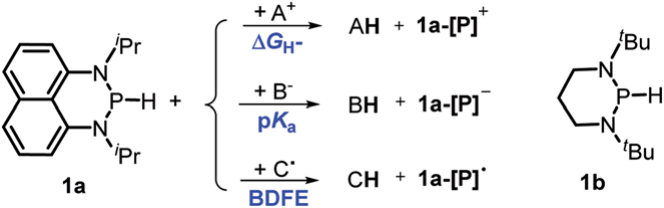
P–H reagents **1a** and **1b** serving as diverse hydrogen donors. **A+**, **B−** and **C˙** are the hydride, proton and hydrogen atom acceptors, respectively.

Herein, we reported a new N-heterocyclic phosphine **1a**, which could act as a H^−^, H˙ and H^+^ donor (Scheme 2). Thermodynamics and kinetics pertinent to these three processes were examined in detail. For comparison, **1b** was investigated as well. Based on the P–H bond energetic studies, we also exploited their versatile applications in the reduction of pyridines, synthesis of bisphosphines, H/D exchange, and activation of carbon–halogen bonds as original examples.

## Results and discussion

### Synthesis

**Fig. 1:**
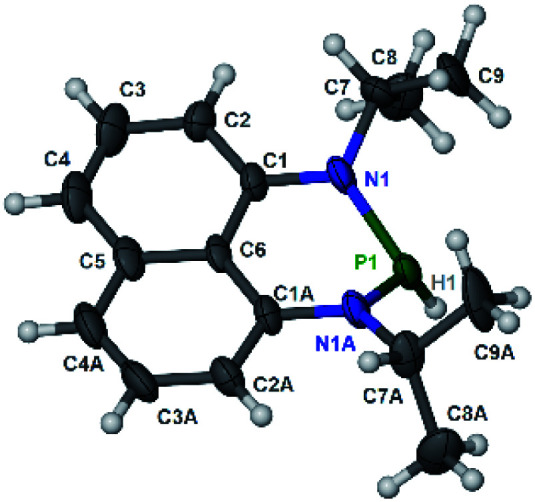
The crystal structure of **1a** (50% probability thermal ellipsoids).^20^ Selected bond lengths (in Å): P1–H1 1.42(2), P1–N1 1.747(6), N1–C1 1.329(9), N1–C7 1.440(8), and C1–C6 1.413(8).

**Scheme 3:**
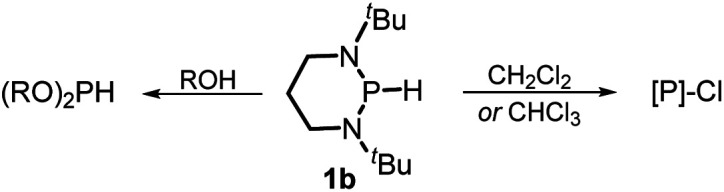
Decomposition of **1b** in alcohols and chlorinated hydrocarbons. [P] = (CH_2_)_3_(N^*t*^Bu)_2_P, R = Me or Et.

The substrate **1a** was prepared *via* a H/Cl exchange between the corresponding phosphoric chloride and LiAlH_4_ in THF according to the literature^15,17^ (see the ESI† for details). LiAlD_4_ was used for deriving the deuterated substrates **1a-D** and **1b-D**. The crystal structure of **1a** was shown in Fig. 1 and features an envelope configuration with the P atom standing out of the naphthalene plane and the P–H bond adopting a flagpole position, similar to the five-membered ring analogs disclosed previously.^15*a*^ And a P–H distance of 1.42(2) Å is slightly shorter than that of the five-membered ring analog.^15^^31^P NMR spectra of **1a** in CD_3_CN show a d*t* peak at 25.66 ppm that is split by the hydrogen atoms on the P atom and isopropyl groups. It moves to the higher magnetic field in comparison with other N-heterocyclic phosphines.^17^ Under an inert atmosphere, **1a** is stable enough for over 12 hours in tetrahydrofuran, acetonitrile, alcohols, toluene, dimethylsulfoxide and chloroform. On the other hand, **1b** is stable in these solvents as well except in alcohols and chlorinated hydrocarbons, where it decomposes rapidly to yield phosphonites (RO)_2_PH^19^ or 2-chloro-1,3,2-diazaphosphinane [P]-Cl (Scheme 3), showing that **1b** is more air- and moisture-sensitive than **1a**.

### Redox properties

The oxidation potentials of **1a** and **1b** were examined by cyclic voltammetry (CV), exhibiting a partially reversible oxidation peak of **1a** to **1a˙+** at 0.23 V (*vs.* Fc^+/0^, Fig. S1a†) and an irreversible oxidation wave of **1b** at 0.47 V (*vs.* Fc^+/0^, Fig. S1b†) in acetonitrile. The low oxidation potentials of **1a** and **1b** indicate their good reducing capacity. It is interesting to find that **1a** is a better electron donor, in spite of being a far poorer hydride donor than **1b** (*vide infra*). Besides, the redox behaviors of the respective phosphinyl radicals (**1a-[P]˙** and **1b-[P]˙**) were also examined through the reduction of the corresponding phosphenium cations (**1a-[P]+** ^21^ and **1b-[P]+**), resulting in the CV peaks at −1.94 V and −2.39 V (*vs.* Fc^+/0^) in acetonitrile, respectively (Fig. S1c and 1d†). The extremely low potentials, especially for **1b-[P]+**, indicate the very high reducing capacity of the corresponding **1a-[P]˙** and **1b-[P]˙** radicals. This suggests that they both have a very promising potential to serve as a super electron donor in organic synthesis to reduce aryl halides.^22^ And indeed, this was verified in the tentative application of the present work in radical hydrodebromination of bromobenzene (see Application part, *vide infra*).

### Thermodynamic driving force and reactivity

#### Hydride transfer (HT)

Hydride transfer from **1a** or **1b** ^17^ to *N*-methylacridinium iodide **A1+** yielded equimolar products (Fig. S3†). To exclude the suspected interruption of the hydridic isopropyl α-C–H, the deuterated substrate **1a-D** was employed, and over 95% deuterated **A1D** was derived (eqn (1) and Fig. S4†). This confirmed that the P–H hydride of **1a** is a stronger donor than that of the isopropyl α-C–H.1
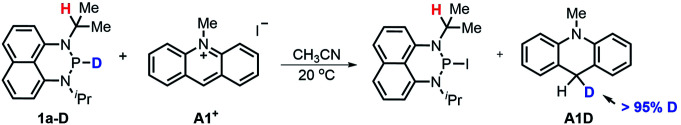


The hydricity (Δ*G*_H^−^_, defined by eqn (2)) of **1a** and **1b** in acetonitrile was determined by measuring their equilibrium constants with the acceptors of known hydricities (eqn (3)). For example, the measured hydride transfer equilibrium constant *K*_eq_(**1a**) of 0.48 (equivalent to 0.43 kcal mol^−1^) between **1a** and phenanthridinuim trifluoromethanesulfonate **A2+** (Δ*G*_H^−^_(**A2H**) = 61.4 kcal mol^−1^)^1*b*^ provided the hydricity Δ*G*_H^−^_ of **1a** to be 61.8 kcal mol^−1^ (see Fig. S5 in the ESI† for details) and other equilibria (in Fig. S6 and S7†) verified this value, giving a mean hydricity of 62.2 ± 1.0 kcal mol^−1^. This demonstrates **1a** to be a moderate hydride donor, similar to the hydride-donating ability of the NADH analogs.^1*b*^ Thus, it should be capable of reducing commonly used hydride acceptors, such as *N*-methylacridinium cations (Δ*G*_H^−^_ = 76 kcal mol^−1^), 9-phenylxanthylium cations (89 kcal mol^−1^) and trityl cations (99 kcal mol^−1^). Similarly, a much lower equilibrium constant *K*_eq_ (**1b**) of 1.6 × 10^−3^ (equivalent to 3.8 kcal mol^−1^, eqn (5) and Fig. S8†) established between **1b** and benzimidazolium perchlorate **A3+** (Δ*G*_H^−^_(**A3H**) = 45.0 kcal mol^−1^)^4*b*^ resulted in Δ*G*_H^−^_(**1b**) being 48.8 ± 1.0 kcal mol^−1^, which is comparable to that of the commonly used good hydride donor, 2,3-dihydrobenzo[*d*]imidazoles.^1*b*^ The good hydridic reactivity of **1b** was illustrated in our recent work in the reduction of various unsaturated compounds.^17^ It is interesting to note that **1b** is about 13 kcal mol^−1^ stronger than **1a** in hydricity, but is 0.24 V weaker in electron-donating ability.2
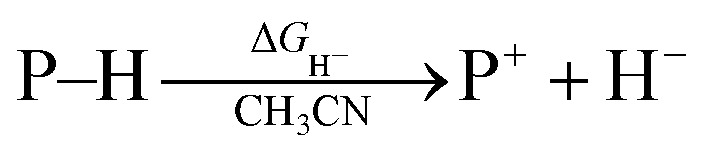

3 Δ*G*_rxn_ = −*RT* ln *K*_eq_ = Δ*G*_H^−^_(**1**) − Δ*G*_H^−^_(**AH**)

4
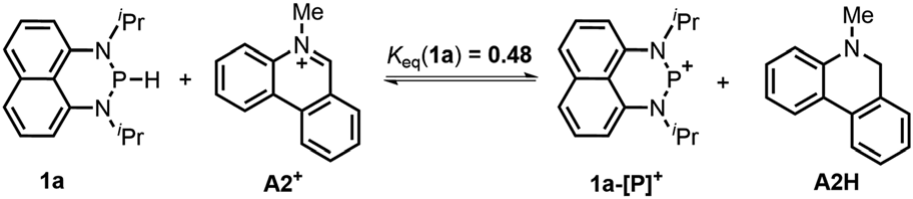
5
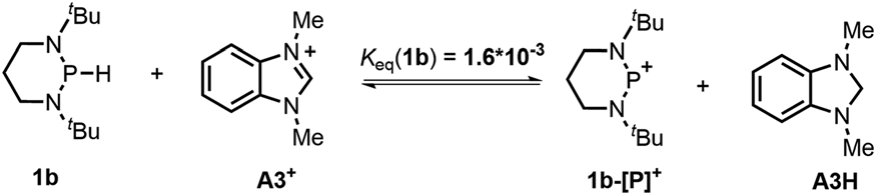


**Scheme 4:**
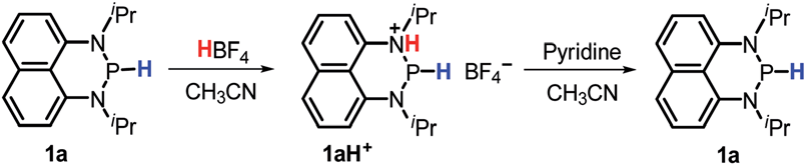
Protonation of **1a** by HBF_4_ and recovery of it by adding pyridine in CD_3_CN.

The hydricity can be used to rationalize the disparate reactivity between **1a** and some reported diazaphospholenium hydrides. The latter compounds were reported to react with strong acid HBF_4_ to give H_2_ and the corresponding phosphenium cations.^15*b*^ However, when **1a** was treated with HBF_4_ (or HOTf), no gaseous product was generated. Instead, a new phosphorus species with a doublet peak at about 80 ppm was detected in the ^31^P NMR spectrum (Fig. S9a and S9b†). To identify this newly formed species, a base pyridine was added to the reaction mixture, which rendered **1a** the primary product with a small amount of unidentified impurities (Fig. S9c†). Consequently, this mysterious P species was speculated to be protonated **1a** (**1aH+**, Scheme 4). Based on the doublet peak of the phosphorus atom in ^31^P NMR spectra (if the extraneous proton was connected to the P atom, a triplet ^31^P peak would be observed) and the unsymmetrical peaks of naphthyl and isopropyl hydrogen atoms in ^1^H NMR spectra (Fig. S10†), the protonated site was thus assigned to the N atom (Scheme 4). We thought that their dissimilarity in reactivity between **1a** and some reported diazaphospholenium hydrides should originate from the relatively weak hydricity of **1a** (Δ*G*_H^−^_ = 62.2 kcal mol^−1^). As a consequence, it makes the H_2_ release unable to compete with its protonation even under the condition of heating (80 °C for 5 hours). The moderate hydricity of **1a** endows it with good tolerance to even strong acids, avoiding direct elimination of dihydrogen. This in turn offers a possibility for **1a** to act as a hydride donor in acid-catalyzed reduction. Lots of examples in this connection^1*a*^ can be found, especially in the hydrogenation of imines by hydride donors with comparable hydricity to **1a** (∼60–70 kcal mol^−1^, like Hantzsch ester,^23^ benzothiazoline^24^ and cyclohexadiene^25^) under the catalysis of Brønsted acids (for examples BINOL-phosphoric acid, trifluoroacetic acid and Tf_2_NH).

**Fig. 2:**
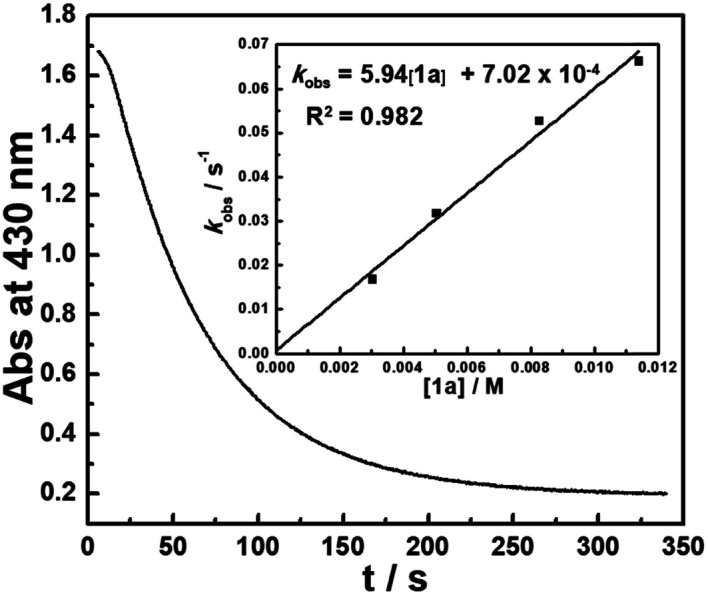
Monoexponential decay of the absorbance Abs (at 430 nm) with the time *t* (s) for the reaction of **1a** (3.03 × 10^−3^ M) with **A1+** (5.00 × 10^−5^ M) in CH_3_CN at 20 °C. Inset: Correlation of *k*_obs_ with [**1a**].

The kinetics of the hydride transfer from **1a** to **A1+** was conducted by following the decay of the absorption of **A1+** in CH_3_CN at 430 nm with a stopped-flow spectrophotometer (Fig. 2, see the ESI† for details). The second-order rate constant *k*_HT_(**1a**) was found to be 5.94 M^−1^ s^−1^ (Inset in Fig. 2 and Table S1†). Moreover, the nucleophilicity of **1a** can be estimated by the second-order rate constant of the reaction of **1a** with **A1+**. Because **1a** has a similar structure with **1b**, based on the nucleophile-specific sensitivity parameter *s*_N_ of **1b** (0.52) and the electrophilicity of **A1+** in our recent work,^17^ the nucleophilicity *N*, of **1a** is estimated to be 8.64. Similarly, the kinetics of **1a-D** with **A1+***k*_HT_(**1a-D**) was determined to be 2.34 M^−1^ s^−1^ (Table S2†). These gave a primary kinetic isotope effect KIE [*k*_HT_(**1a**)/*k*_HT_(**1a-D**)] of 2.5. A comparison with the previously determined *k*_HT_(**1b**) of 5.41 × 10^2^ M^−1^ s^−1^ for the reaction of **1b** with **A1+** in our earlier work^17^ indicates that **1b** is about 100 times more reactive than **1a**. This is exactly in accordance with the order of their hydricities (Δ*G*_H^−^_) of **1a***vs.***1b** found here.

#### Hydrogen atom transfer (HAT)

To examine the possibility of a HAT reaction from diazaphosphinane substrates **1**, the reactions of **1a** and **1b** with the 2,4,6-tri-*tert*-butyl-phenoxyl radical **O˙** were conducted, and yielded 2,4,6-tri-*tert*-butylphenol **OH** and the corresponding phosphorus species (eqn (6)) without generation of bisphosphines (compared to the reaction of **1a** or **1b** with AIBN, Table 1). A stoichiometric study found a [**1**]/[**O˙**] ratio of 1 : 2, consistent with the product analysis (Fig. S11 and S12†).6
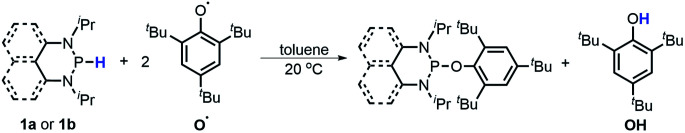


**Table 1:** Applications of **1a** and **1b** in organic syntheses

Entry	Reactant	Condition	Product	Yield	Type
(1)	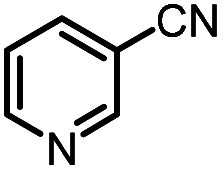	**1a-[P]+** (15 mol%), HBpin (1.5 equiv.), CH_3_CN, 80 °C, 36 h	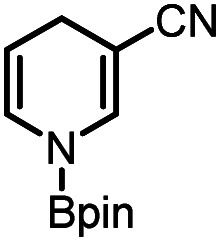	40%^a^	HT
(2)	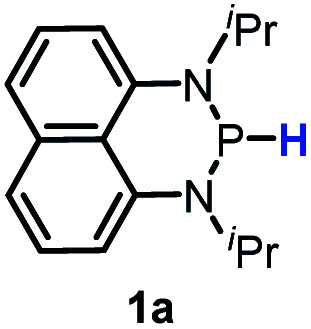	^*t*^BuOK, CD_3_CN, 20 °C, 10 min	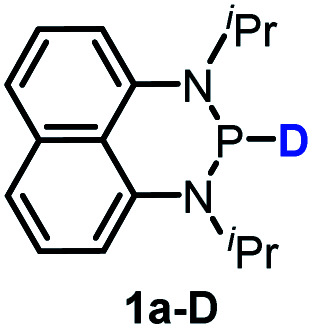	Quant.^b^	PT
(3)	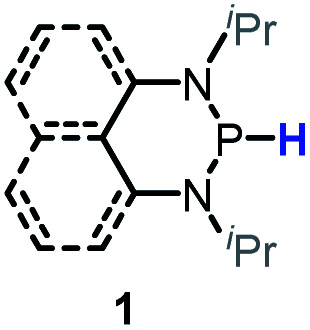	AIBN (1.5 equiv.), C_6_D_6_, 80 °C, 3 h	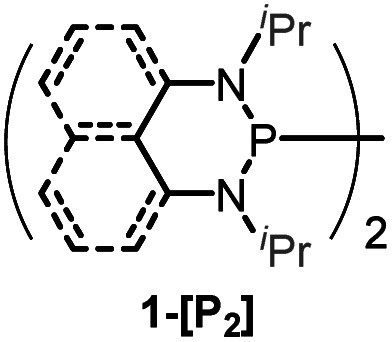	>90%^b^	HAT
(4)	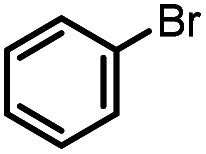	**1b** (1.5 equiv.), AIBN 15 mol%, toluene-d_8_, 90 °C, 5 h	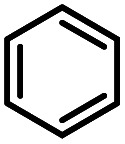	>90%^c^	HAT&ET

a Isolated yield.

b NMR yields determined by the amount of phosphorus species.

c NMR yields with 1,3,5-trimethoxybenzene as the internal standard.

**Scheme 5:**
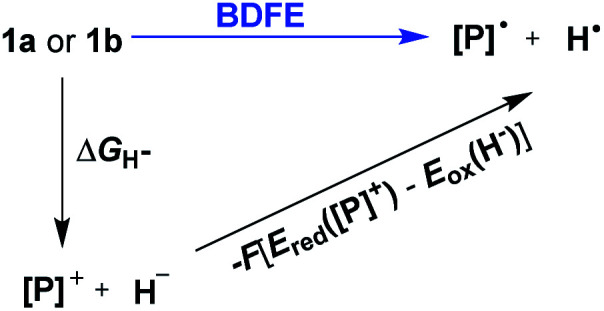
Thermodynamic cycle for deriving the P–H BDFEs of **1a** and **1b**.

To evaluate the hydrogen atom donability of diazaphosphinanes (*i.e.*, P–H BDFE), the thermodynamic cycle was applied on the basis of the Δ*G*_H^−^_ and *E*_red_(**[P]+**) in hand (Scheme 5 and eqn (7); *FE*_ox_(**H−**) is −26.0 kcal mol^−1^^1*c*^ in acetonitrile *vs.* Fc^0/+^).^26^ Substituting the known values into eqn (7) ^26*a*,27^ gives P–H BDFEs of 80.9 ± 2.0 and 77.9 ± 2.0 kcal mol^−1^ for **1a** and **1b**, respectively, being comparable with those of phenol and thiophenol antioxidants. Therefore, they could transfer hydrogen atoms smoothly to acceptors with X–H BDFEs around or larger than 78 kcal mol^−1^, such as 2,4,6-tri-*tert*-butyl-phenoxyl **O˙** (77.1 kcal mol^−1^), CN(CH_3_)_2_C˙ (about 88 kcal mol^−1^) and phenyl radicals (104.7 kcal mol^−1^).^5*b*,28^ Compared to **1b**, though the fused aryl groups attenuate the hydricity of **1a** by 13 kcal mol^−1^ thermodynamically, they show a negligible effect on the HAT reactivity.

7 BDFE = Δ*G*_H^−^_ − *F*(*E*_red_([**P**]^+^) − *E*_ox_(**H−**)) = Δ*G*_H^−^_ − 23.06*E*_red_([**P**]^+^) − 26.0 (kcal mol^−1^)

**Scheme 6:**
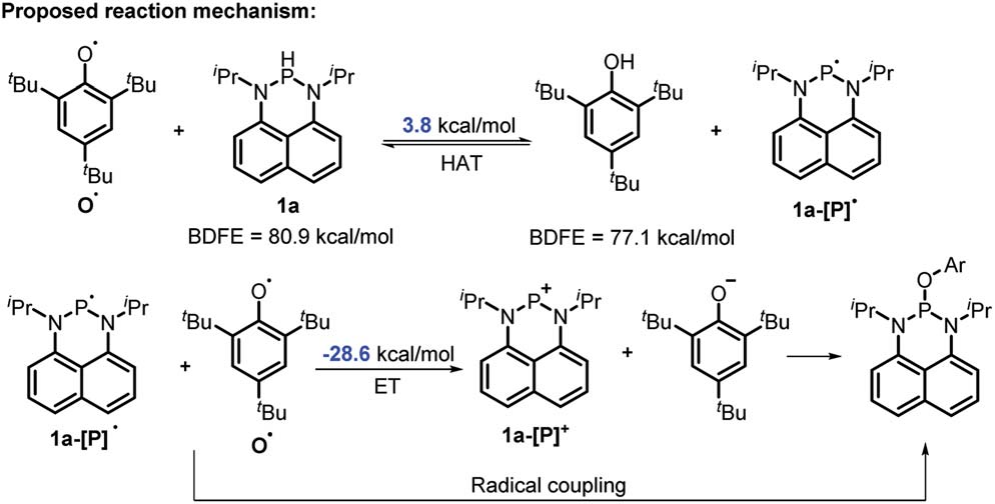
The possible mechanism for the reaction of **1a** with **O˙**. Ar = 2,4,6-tri-*tert*-butyl-phenyl group.

The reaction mechanism of **1a** with radical **O˙** (eqn (6)) is proposed in Scheme 6 based on the derived thermodynamic data (the reaction of **1b** with **O˙** is similar). Since hydrogen atom transfer from **1a** to **O˙** is slightly endothermic (∼4 kcal mol^−1^), it implies the possibility for a reversible HAT. This reversible HAT could be further driven to the far right by the largely exothermic electron transfer, ET of **1a-[P]˙** radical (*E*_ox_(**1a-[P]˙**) = −1.94 V) or a follow-up radical coupling. The observed stoichiometric ratio of 1 : 2 for the reactants [**1**] *vs.* [**O˙**] is in exact accordance with the HAT mechanism proposed. On the other hand, a suspected proton transfer between **1a** and the **O˙** radical can be easily ruled out due to the very weak acidity of **1a** (*vide infra*) and strong acidity (p*K*_a_ of ∼−3)^5*b*^ reported for the phenol radical cations (eqn (8)). The other suspected possibility, *i.e.* the ET path between **1a** and the **O˙** radical (*E*_red_ = −0.70 V ^5*b*^) can also be excluded by a similar strategy on the basis of their respective thermodynamics determined, that indicates that the ET from **1a** to **O˙** is remarkably uphill (Δ*G*_ET_ = 21.4 kcal mol^−1^, eqn (9)).^13,29^ It is thus safe to conclude that the initial HAT should be the rate-determining step (RDS) for the reaction expressed in eqn (6).8
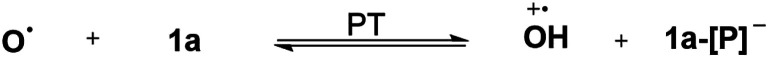
9
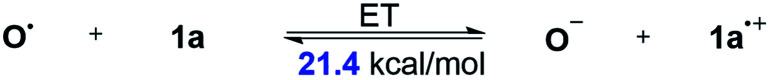


The rate constants of this hydrogen atom transfer from **1a** and **1a-D** to radical **O˙** were measured to be a *k*_HAT_(**1a**) of 0.70 M^−1^ s^−1^ and a *k*_HAT_(**1a-D**) of 2.24 × 10^−2^ M^−1^ s^−1^, respectively (Tables S3 and S4†). This intermediately points out an extremely large KIE(**1a**) of 31.3, implying a tunneling effect in this HAT process. Such a large KIE has been found in biochemical^30^ and transition-metal systems,^31^ but seldom observed in organic HAT processes. Similarly, the rate of the analogous HAT of **1b***k*_HAT_(**1b**) was determined to be 0.74 M^−1^ s^−1^ (Table S5†). The quite close *k*_HAT_ for **1a** and **1b** agrees well with their comparable P–H BDFEs (80.9 *vs.* 77.9 kcal mol^−1^, *vide supra*), verifying their similar HAT reactivity. However, the same kinetic study for **1b-D** gave a *k*_HAT_(**1b-D**) of 0.12 M^−1^ s^−1^ (Table S6†), resulting in a primary KIE(**1b**) of 6.2 with no tunneling phenomenon detected.

To understand this discrepancy, temperature-dependent kinetics were further performed (Tables S7–S10†). The ratio of Arrhenius pre-factors [*A*(**1a**)/*A*(**1a-D**)] was obtained to be 0.021, and the activation energy difference [*E*_a_(**1a**) − *E*_a_(**1a-D**)] was −5.53 kcal mol^−1^ (Fig. S2 and Table S11†). These kinetic characteristics [*A*(**1a**)/*A*(**1a-D**) ≪ 1 and *E*_a_(**1a**) − *E*_a_(**1a-D**) ≪ 0] are in fact in line with the tunneling model illustrated by Klinman,^30*a*^ which features a “through-the-barrier” transition state near the top of the classical barrier. In the **1b** system, on the other hand, the normal primary KIE of 6.2, *A*(**1b**)/*A*(**1b-D**) of 3.39 and [*E*_a_(**1b**) − *E*_a_(**1b-D**)] of −0.38 kcal mol^−1^ all support a classical “over-the-barrier” transition state. A more comprehensive investigation of the tunneling issue is presently underway.

#### Proton transfer (PT)

It's known that a good hydride donor usually exhibits poor acidity. This explains why the acidity of widely-used X–H type hydride donors, *e.g.* 2,3-dihydrobenzo[*d*]-imidazoles, has never been determined in the absence of metal-coordination. The hydricity of the present N-heterocyclic phosphines is also quite good (*vide supra*), hence an investigation of their acidic reactivity cannot be expected to be too straightforward. Nevertheless, as we learned from the initial failures in deprotonating **1a** or **1b** with some ordinary strong bases, such as 1,8-diazabicyclo[5,4,0]-7-undecene (DBU, p*K*_a_ = 24.34 in CH_3_CN), 1,3,4,6,7,8-hexahydro-2*H*-pyrimido[1,2-*a*]pyrimidine (TBD, p*K*_a_ = 26.03) and (*tert*-butylimino)tris(pyrrolidino)-phosphorane (BTPP, p*K*_a_ = 28.42)^28^ (see the ESI† for details), we finally found that the H/D exchange of the **1a** P–H proton in the presence of stoichiometric ^*t*^BuOK in CD_3_CN (eqn (10) and Fig. S13†) was accomplished in about 10 minutes at room temperature,^32^ whereas the same operation for **1a-D** in CH_3_CN was finished in 8 hours at room temperature (eqn (11) and Fig. S14†). The reversible H/D exchange of **1a** in acetonitrile under the promotion of ^*t*^BuOK suggested that the acidity of **1a** reaches the measurable acidity limitation in acetonitrile (see the ESI† for details).^33^ As for **1b** with much stronger hydricity, its acidity is, unfortunately, too weak to examine in solution by any existing experiment. However, it is worth noting that the acidity of a P–H bond can be significantly enhanced upon activation by coordination with transition metals^34^ or other Lewis acids.^35^10
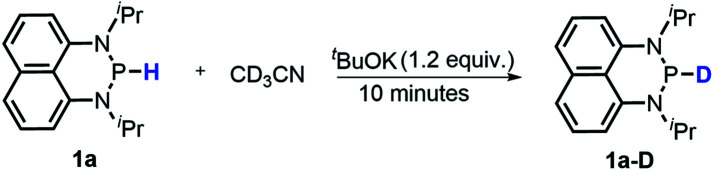
11
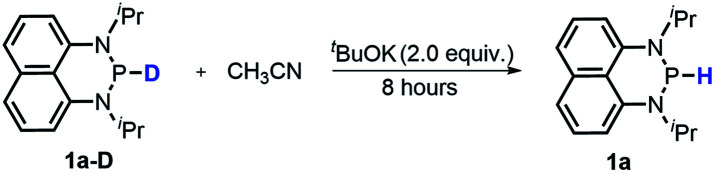


### Applications in syntheses

Based on the above thermodynamic and kinetic investigations, the potential of applying **1a** and **1b** in organic syntheses, encompassing the diverse hydrogen (H^+^, H˙ and H^−^) or electron transfer, was preliminarily explored and showcased herein.

Catalytic reduction of pyridines is a prevailing strategy for the synthesis of dihydropyridines. Recently, Kinjo *et al.*^16*g*^*and* Speed *et al.*^36^ exploited 1,3,2-diazaphosphenium-catalyzed hydroboration of pyridines with good regio- and chemo-selectivity.^16*g*^ However, in their system, the substrate 3-CN-pyridine gave a mixture of three dihydropyridine isomers. To our delight, we found that under the catalysis of 15 mol% of our **1a-[P]+**,^37^ the sole 1,4-hydroborated isomer of this reaction was selectively produced in a moderate yield of 40% (entry 1 in Table 1 and Fig. S15†). The good regio-selectivity may be ascribed to the steric factors of the isopropyl group on the nitrogen atom of **1a** based on Kinjo's DFT calculations.^16*g*^ This good regio-selectivity exhibits the catalytic potential of **1a** in the reduction of unsaturated compounds.

Moreover, the acidic character of **1a** (*vide supra*) offers a more economic method for the synthesis of deuterated reagent **1a-D** (entry 2 in Table 1) by employing the much cheaper deuterated solvent CD_3_CN instead of expensive LiAlD_4_ in an almost quantitative yield (see the ESI† for details about the synthetic method). Meanwhile, it also provides a new approach for isotope labeling of many other hydridic species. The generation of the hydridic hydrogen from a “protic” solvent as illustrated here may serve as an example of Umpolung which was also proposed by Gudat *et al.* for the P–H bond.^15*a*^

Since bisphosphines can easily decompose into phosphinyl radicals,^38^ they could serve as radical reservoirs for relevant studies and applications. Based on thermodynamic analysis, CN(CH_3_)_2_C˙ (C–H BDFE ≈ 85 kcal mol^−1^), generated from the homolysis of azo-bis-isobutyronitrile (AIBN), could abstract the hydrogen atoms from **1a** (P–H BDFE = 80.9 kcal mol^−1^). Then, the generated phosphinyl radical **1a-[P]˙** coupled quickly with each other to yield bisphosphines (entry 3 in Table 1, Fig. S16† for **1a** and Fig. S17† for **1b**). The targeted dimers^39^ are readily extracted from reaction mixtures in a high yield. This transformation, together with Gudat's photochemical dehydrocoupling,^18^ offers easy access to bisphosphines, avoiding complicated and harsh operations and workups required in other synthetic processes using metals such as sodium^38*d*^ and magnesium^38*a*–*c*^.

Furthermore, we attempted to design a new radical transformation by an integrated use of hydrogen atoms and electron transfers from N-heterocyclic phosphines. This was inspired by the reactions of AIBN with **1a** and **1b**, where phosphinyl radicals can be easily generated from their P–H precursors through hydrogen atom release. The highly negative oxidation potentials of phosphinyl radicals imply their competency in the reduction of challenging substrates. If a substrate in the reaction system is sufficiently oxidative, its single-electron capture from phosphinyl radicals may be able to compete with radical coupling. In such cases, subsequent transformations may be triggered. Based on the redox potentials of **1b-[P]˙** (*E*_ox_ = −2.39 V *vs.* Fc^+/0^, the same with the *E*_red_(**1b-[P]+**)) and bromobenzene (*E*_red_ = −2.8 V),^40^ hydrodehalogenation of bromobenzene by **1b** was performed in toluene with 15 mol% AIBN as the initiator (entry 4 in Table 1, Fig. S18†). After 5 hours, the expected reduction product, benzene was obtained in over 90% yield. Due to the poor reduction ability of **1a-[P]˙**, the reaction could not proceed when **1a** was used.^41^ Therefore, it could be anticipated that these N-heterocyclic phosphines may open up a promising avenue for the development of super electron donors.

## Conclusions

Hydrogen and electron transfers from phosphines **1a** and **1b** were thermodynamically and kinetically investigated in acetonitrile. The P–H bond of **1a** can participate in reactions involving formal transfer of a hydride/hydrogen atom/proton to a substrate under respective conditions in the absence of the usually required transition-metal- or Lewis acid-mediation. It was observed that **1a** is a moderate hydride and hydrogen atom donor, but a poor Brønsted acid. In comparison, **1b** is a good hydride donor and a moderate hydrogen atom donor; but did not show a detectable acidic reactivity even under the strongest basic solution. With regard to electron donability, **1a** (*E*_ox_(**1a**) = 0.23 V) is superior to **1b** (*E*_ox_(**1b**) = 0.47 V), contrasting with the order of their hydridic reactivity. Their corresponding phosphinyl radicals are very strong electron donors as reflected by their extremely negative redox potentials. Kinetic studies revealed a tunneling contribution (KIE = 31.3) to the hydrogen atom transfer from **1a** to the phenoxy radical. Based on the derived energetic parameters, their synthetic applications were tentatively exploited. Preliminary results from the synthesis attempts by using the new reagents **1a** and **1b** confirmed their competence as effective hydrogen and electron donors in various redox transformations. The effectiveness of applying the relevant physicochemical parameters in the analysis and design of hydrogen transfer reactions was also shown.

## Conflicts of interest

There are no conflicts to declare.

## Supplementary Material

SC-011-C9SC05883D-s001

SC-011-C9SC05883D-s002
