# Supplementary material for: Diazaphosphinanes as hydride, hydrogen atom, proton or electron donors under transition-metal-free conditions: thermodynamics, kinetics, and synthetic applications
Source: Chem Sci. 2020 Mar 5;11(14):3672–9. doi: 10.1039/c9sc05883d (PMC8152589; doi:10.1039/c9sc05883d)
Supplement: SC-011-C9SC05883D-s001 [file SC-011-C9SC05883D-s001.pdf]

Electronic Supplementary Material (ESI) for Chemical Science.
This journal is © The Royal Society of Chemistry 2020

Diazaphosphinanes as Hydride, Hydrogen-atom, Proton or Electron Donors under Transition-metal-free Conditions: Thermodynamics, Kinetics and Synthetic Applications

Jingjing Zhang[†], Jin-Dong Yang,[†,]* Jin-Pei Cheng[†,‡,]*

[†]Center of Basic Molecular Science (CBMS), Department of Chemistry, Tsinghua University, Beijing, 100084, China.

[‡]State Key Laboratory of Elemento-organic Chemistry, Nankai University, Tianjin, 300071, China.

Contents

1. General information. 2
2. The synthesis of **1a**, **1a-D** and **1b-D**. 3
3. The synthesis of **1a-[P]$^+$** and **1b-[P]$^+$**. 4
4. Cyclic voltammetry 5
5. Kinetics for the reactions of **1a** and **1a-D** with **A1$^+$**. 6
6. Kinetics for the reactions of **1a** and **1a-D** with 2,4,6-tri-tert-butylphenol **O$^•$**. 6
7. Kinetics for the reactions of **1a** and **1a-D** with 2,4,6-tri-tert-butylphenol **O$^•$** at 292 K – 320 K. 8
8. Kinetics for the reactions of **1b** and **1b-D** with 2,4,6-tri-tert-butylphenol **O$^•$** at 292 K – 322 K 9
9. Kinetics for the reactions of **1b** and **1b-D** with 2,4,6-tri-tert-butylphenol **O$^•$** at 292 K – 322 K 11
10. The reactions of **1a** and **1a-D** with *N*-methylacridinium ion **A1$^+$** in CD_3CN. 11
11. The equilibrium between **1a** and **A2$^+$** in CD_3CN 14
12. The equilibrium between **1b** and **A3$^+$** in CD_3CN. 18
13. The reactions of **1a** with HOTf and HBF_4•Et_2O in CD_3CN. 19
14. The reactions of **1a** and **1b** with **O$^•$** in toluene-d_8. 21
15. The reaction of **1a** and **1a-D** with ^{t}BuOK. 23
16. The reaction of **1a-[P]$^+$** with substituted pyridine. 25
17. The reactions of **1a** and **1b** with AIBN in C_6D_6. 28
18. The hydrodehalogenation reaction of bromobenzene. 30
19. DFT Calculations. 31
20. NMR spectra. 33
21. Crystal data of **1a**. 42
22. SMD-M06-2X/6-31+G(d) calculated cartesian coordinates and energies. 42
23. Reference. 47

1. General information.

Chemicals: **1a**, **1a-D**, **1b-D** and **1b-[P]**$^+$ were synthesized in this work and the methods were shown below. **1a-[P]**$^+$[1] and **1b**[2] were synthesized according to literature procedures, and all ^{1}H NMR as well as ^{31}P NMR spectra were shown in the NMR part of Supporting Information. *N*-methylacridinium iodide **A1**$^+$,[3] phenanthridinuim trifluoromethanesulfonate **A2**$^+$,[4] benzimidazolium perchlorate **A3**$^+$,[5] 3,5-bis(ethoxycarbonyl)-1,2,6-trimethylpyridin-1-ium tetrafluoroborate **A4**$^+$,[6] and 2,4,6-tri-*tert*-butylphenol **O**$^•$[7] were prepared as described before. CH_3CN was purchased from J&K Chemical (99.9 %, Extra dry, water < 10 ppm, J&K seal). THF, n-pentane and toluene were distilled by standard solvent treatment methods. Unless otherwise mentioned, all chemicals were purchased from commercial sources and used without further purification. Reaction temperature refers to temperature of an aluminum heating block or a silicon oil bath, which was controlled by an electronic temperature modulator from IKA.

Reactions and characterizations: All reactions involving **1a** and **1b** were carried out in very dried glass wares under an argon atmosphere using Schlenk technique until the end of the reactions. ^{1}H and ^{13}C NMR spectra were recorded in acetonitrile-d_3 (δ_H 1.94, δc 118.26), toluene-d_8 δ_H 7.09, 7.01, 6.97, 2.08, δc 137.48, 128.87, 127.96, 125.13, 20.43) and C_6D_6 (δ_H 7.16, δc 128.06) on 400 MHz NMR instrument at Center of Basic Molecular Science (CBMS) of Tsinghua University. Data for ^{1}H NMR spectra are reported as follows: chemical shift (multiplicity, coupling constants, number of hydrogens). Abbreviations are as follows: s (singlet), d (doublet), t (triplet), q (quartet), m (multiplet) and br (broad).

Electrochemical: All samples were prepared and all electrochemical experiments were performed in an inert Ar atmosphere. The supporting electrolyte was $[Bu_4N]PF_6$, which was recrystallized three times by EtOH and dried about 12 hours before use, and the concentration is about 0.1 M in acetonitrile. A standard three-electrode cell consists of a glassy carbon disk as work electrode, a platinum wire as a counter electrode, and 0.1 M $AgNO_3$/Ag (in 0.1 M $[Bu_4N]PF_6$-acetonitrile) as reference electrode. Ferrocene ($Fc^{0/+}$) was used as an external reference and was found to be 0.04 V with respect to our reference electrode. The sample concentrations of **1a**, **1b**, **1a-[P]**$^+$ and **1b-[P]**$^+$ are about 1.0 mM. The scan rate was 100 mV/s. All potentials are reported in volts (V) *vs.* $Fc^{+/0}$.

***Kinetics*:** The rates of all reactions were determined by UV/Vis spectroscopy in CH_3CN by using Stopped-flow apparatus. The temperature of the solutions was maintained at 20 ± 0.2 °C by using circulating bath cryostats. **1a** and **1b** are air-sensitive compounds, so all solutions used for measurement were prepared in glove box. Concentrations of approximate 10^{-5} M were used for the acceptors **A**$^+$ and **O**$^•$ to achieve an initial absorbance A_0 of approximate 1.0. In order to satisfy pseudo first-order kinetics with $k_{obs} = k_2[\mathbf{1}]_0 + C,$ the concentrations of nucleophiles **1** were selected by the criterion $[\mathbf{1}]_0/[\mathbf{A}^+ \text{ or } \mathbf{O}^•]_0 > 10$. All concentrations are specified in the Tables below. Pseudo-first order rate constants k_{obs} (s^{-1}) were obtained by fitting the monoexponential function $A_t = A_0\exp(-k_{obs}t) + C$ to the observed time-dependent absorbance A_t. To obtain the second-order rate constants k_{HT} and k_{HAT} (M^{-1} s^{-1}), each acceptor-donor combination was measured in 3 to 5 different concentrations of **1a** or **1b**. For hydride transfers, $k_{obs} = k_{HT}[\mathbf{1}]$ and for hydrogen-atom

transfers, $k_{obs} = 2k_{HAT}[\mathbf{1}]$. As for the measurements for Arrhenius and Eyring correlations, kinetics were performed at 5 different temperatures from 292 K to 322 K. Kinetic runs were reported three times at each temperature.

Acidity estimation: *N*-heterocyclic phosphines with strong hydricity are generally too low to be determined or synthetically used. Although the hydricity of phosphines have been extensively exploited for synthetic applications, their acidic properties remain elusive. This stimulated us to identify the feasibility of **1a** and **1b** as proton donors. We first chose several strong bases, such as 1,8-diazabicyclo[5,4,0]-7-undecene (DBU, pK_a = 24.34 in acetonitrile), 1,3,4,6,7,8-hexahydro-2H-pyrimido[1,2-a]pyrimidine (TBD, pK_a = 26.03) and (*tert*-butylimino)tris(pyrrolidino)-phosphorane (BTPP, pK_a = 28.42) to deprotonate **1a** and **1b**.[8] Disappointingly, only negative results were obtained. When stoichiometric ^{t}BuOK was added to the CD_3CN solution of **1a**, it is pleasant to find that a fast H/D exchange of P-H hydrogen was completed in about 10 minutes Such a result definitely confirmed the acidic reactivity of P-H hydrides. Reversibly, combing ^{t}BuOK with **1a-D** in CH_3CN resulted in an almost quantitative recover of **1a** after 8 hours. According to the acidities of CH_3CN (pK_a = 31.3) and ^{t}BuOH (pK_a = 32.3) in DMSO solution,[8] the ^{t}BuOK may be a very strong base in CH_3CN which could react with solvent CH_3CN or **1a**. Present results indicated that a reaction between **1a** and ^{t}BuOK was established in CH_3CN (Eq. S1), and, a complete conversion of P-H into P-D demonstrates that **1a** may reach the limited value of acidity in CH_3CN.

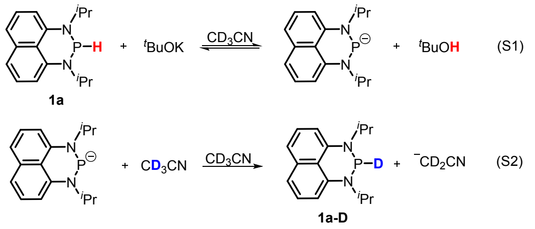

2. The synthesis of 1a, 1a-D and 1b-D.

<u>Preparation of 1,3-diisopropyl-2,3-dihydro-1H-naphtho[1,8-de][1,3,2]diazaphosphinine 1a.</u>

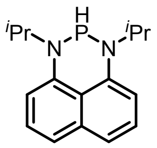

1a

A yellow solution of 2-chloro-1,3-diisopropyl-2,3-dihydro-1H-naphtho[1,8-*de*][1,3,2]diazaphosphinine[1, 9] (1.6 g, 5.0 mmol) in THF (20 mL) was cooled to 0 °C, and 1.0 M $LiAlH_4$ solution in THF (1.25 mL, 1.25 mmol) was slowly added into the mixture. The mixture was stirred for 10 min at 0 °C and then warmed to room temperature for 1 hour. All solvents were evaporated in vacuum, and the residue was extracted with n-hexane (50 mL) and then filtered under Ar atmosphere. The filtrate was evaporated, producing **1e** as yellow solid, 1.1 g (80%).

^{1}H NMR (400 MHz, CD_3CN) δ 7.35 – 7.17 (m, 4H), 6.86 (dd, *J* = 5.9, 2.9 Hz, 2H), 5.64 (d, *J* = 224.7 Hz, 1H), 4.30 – 4.20 (m, 2H), 1.38 (dd, *J* = 8.5, 6.7 Hz, 12H). **^{13}C NMR** (101 MHz, CD_3CN) δ 146.38 (d, *J* = 4.8 Hz), 136.62, 125.79, 120.77, 119.51, 107.94, 50.23 (d, *J* = 23.3 Hz), 22.22 (d, *J* = 14.6 Hz), 22.05 (d, *J* = 12.3 Hz). **^{31}P NMR** (162 MHz, CD_3CN) δ 25.66 (dt, *J* = 225.0, 13.6 Hz). **ESI-HR** calcd for $C_{16}H_{22}N_2P$ (M-H$^+$) 273.1515, found 273.1504.

<u>Preparation of 1,3-diisopropyl-2,3-dihydro-1H-naphtho[1,8-de][1,3,2]diazaphosphinine-2-d 1a-D.</u>

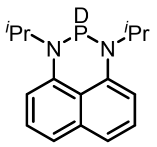

1a-D

The preparation of **1a-D** was the same as **1a**. A yellow solution of 2-chloro-1,3-

diisopropyl-2,3-dihydro-1H-naphtho[1,8-*de*][1,3,2]diazaphosphinine[1, 9] (1.6 g, 5.0 mmol) in THF (20 mL) was cooled to 0 °C, and 2.0 M $LiAlD_4$ solution in THF (0.6 mL, 1.25 mmol) was slowly added into the mixture. The mixture was stirred for 10 min at 0 °C and then warmed to room temperature for 1 hour. All solvents were evaporated in vacuum, and the residue was extracted with n-hexane (50 mL) and then filtered under Ar atmosphere. The filtrate was evaporated, producing **1a-D** as yellow solid, 1.0 g (73%).

^{1}H NMR (400 MHz, CD_3CN) δ 7.35 – 7.18 (m, 4H), 6.85 (dd, *J* = 5.6, 3.0 Hz, 2H), 4.29 – 4.19 (m, 2H), 1.39 – 1.36 (m, 12H). **^{31}P NMR** (162 MHz, CD_3CN) δ 24.44 (tt, *J* = 48.6 Hz, 13.0 Hz).

<u>Preparation of 1,3-di-tert-butyl-1,3,2-diazaphosphinane-2-d 1b-D.</u>

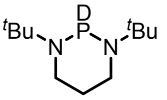

1b-D

A solution of 1,3-di-tert-butyl-2-chloro-1,3,2-diazaphosphinane[2] (2.5 g, 10.0 mmol) in THF (40 mL) was cooled to 0 °C, and 2.0 M $LiAlD_4$ solution (1.3 mL, 2.5 mmol) was slowly added. The mixture was stirred for 10 min at 0 °C and then warmed to room temperature for 3 hours. All solvents were evaporated in vacuum. Then the residue was extracted with n-hexane (50 mL) and filtered. The solvent of filtrate was removed and produced **1b-D** yellow oil 1.6 g (74%).

^{1}H NMR (400 MHz, toluene-d_8) δ 3.42 – 3.26 (m, 2H), 2.88 – 2.82 (m, 2H), 1.81 – 1.72(m, 1H), 1.34 – 1.31(m, 1H), 1.22 (s, 18H). **^{31}P NMR** (162 MHz, toluene-d_8) δ 48.53 (t, *J* = 34.0 Hz).

3. The synthesis of 1a-[P]$^+$ and 1b-[P]$^+$.

<u>Preparation of 1a-[P]$^+$.</u>

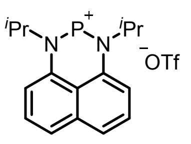

1a-[P]$^+$

$AgSO_3CF_3$ (AgOTf) (0.51 g, 2.0 mmol) was added into the solution of 2-chloro-1,3-diisopropyl-2,3-dihydro-1H-naphtho[1,8-*de*][1,3,2]diazaphosphinine[1, 9] (0.61 g, 2.0 mmol) in 10 mL of acetonitrile. After 1 hours stirring, the reaction mixture was filtered through a glass frit packed with Celite. The filtrate was concentrated to yield red solid **1a-[P]$^+$** 0.78 g (93%).

^{1}H NMR (400 MHz, CD_3CN) δ 7.47 – 7.33 (m, 4H), 6.94 (dd, *J* = 5.7, 2.9 Hz, 2H), 4.40 – 4.31 (m, *J*, 2H), 1.54 (dd, *J* = 6.7, 1.1 Hz, 12H). **^{31}P NMR** (162 MHz, CD_3CN) δ 111.47 (s). **^{13}C NMR** (101 MHz, CD_3CN) δ 137.22, 135.78, 126.75, 121.43, 119.48, 108.95, 51.13 (d, *J* = 27.6 Hz), 21.33 (d, *J* = 17.5 Hz).

^{1}H NMR (400 MHz, toluene-d_8) δ 7.19 (d, *J* = 8.0 Hz, 2H), 7.12 – 7.08 (m, 2H), 6.51 (d, *J* = 7.7 Hz, 2H), 3.89 (tt, *J* = 13.5, 6.6 Hz, 2H), 1.32 (dd, *J* = 6.6, 2.2 Hz, 12H). **^{31}P NMR** (162 MHz, toluene-d_8) δ 141.26 (s).

The NMR spectroscopic data are in good agreement with those in the literature.[1]

<u>Preparation of 1b-[P]$^+$.</u>

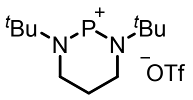

1b-[P]$^+$

The preparation of **1b-[P]$^+$** was the same as **1a-[P]$^+$**. $AgSO_3CF_3$ (AgOTf) (0.51 g, 2.0 mmol) was added into the solution of 1,3-di-tert-butyl-2-chloro-1,3,2-diazaphosphinane[2] (0.50 g, 2.0 mmol) in 10 mL of acetonitrile. After 1 hours stirring, the reaction mixture was filtered through a glass frit packed with Celite. The filtrate was concentrated to yield white solid **1b-[P]$^+$** 0.67 g (92%).

^{1}H NMR (400 MHz, CD_3CN) δ 3.39 (dd, *J* = 11.3, 5.8 Hz, 4H), 2.10 – 1.99 (m, 2H), 1.45 (d, *J* = 3.1 Hz, 18H). **^{13}C NMR** (101 MHz, CD_3CN) δ 62.63 (d, *J* = 18.7 Hz), 43.98 (d, *J* = 7.8 Hz), 28.52 (d, *J* =

14.6 Hz), 24.43. **^{31}P NMR** (162 MHz, CD_3CN) δ 248.59 (s). **ESI-HR** calcd for $C_{11}H_{24}N_2P$ (M^+) 215.1672, found 215.1667.

4. Cyclic voltammetry.

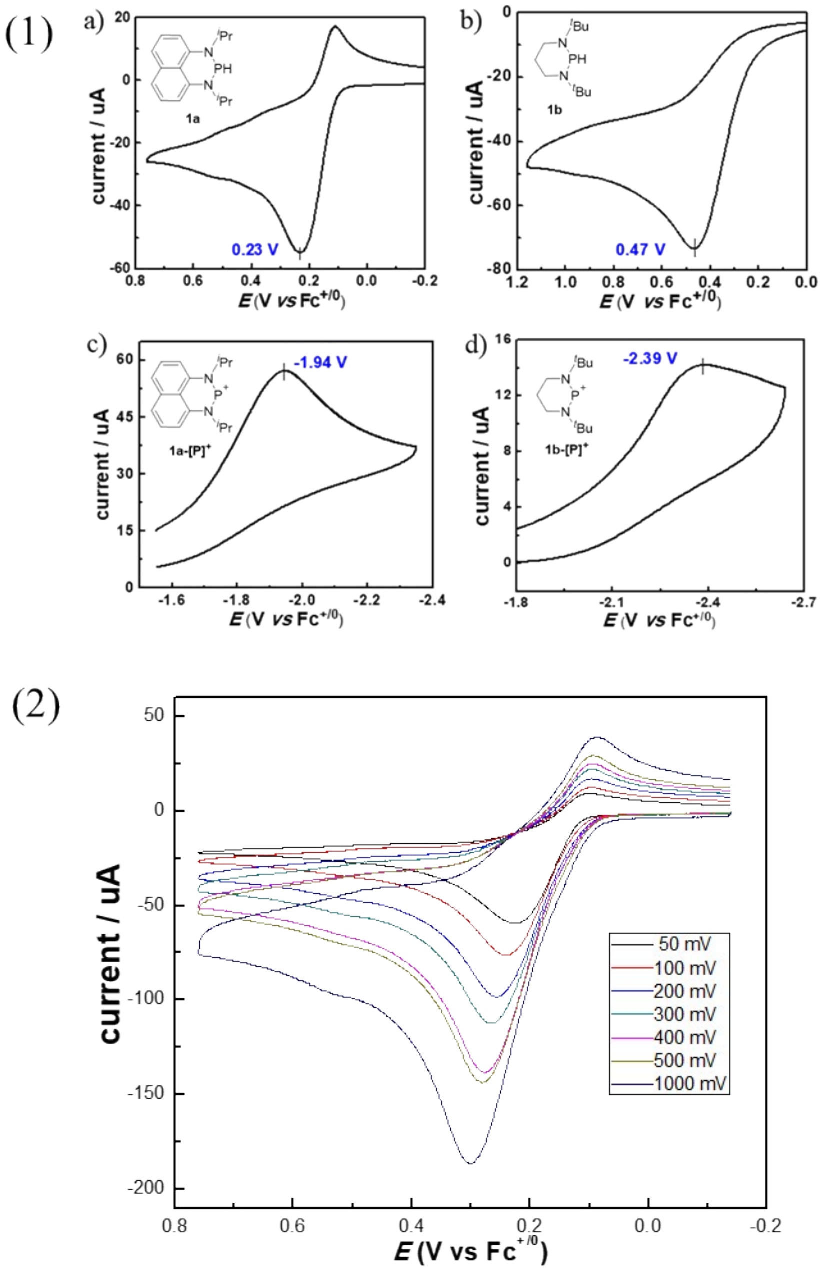

Figure S1. 1) Cyclic voltammetry for the oxidation of a) 1.2 mM **1a**, b) 1.2 mM **1b**, and the reduction of c) 3 mM **1a-[P]**$^+$ and d) 1 mM **1b-[P]**$^+$ in acetonitrile at 20 °C with 0.1 M $[Bu_4N]PF_6$ as supporting electrolytes and Ferrocene ($Fc^{0/+}$) as an external reference at a scan rate of 100 mV/s. 2) Cyclic voltammetry for the oxidation of 1.2 mM **1a** at different sweep rates from 50 mV to 1000 mV.

5. Kinetics for the reactions of 1a and 1a-D with A1$^+$.

Table S1. Kinetics of the reaction of **1a** with **A1**$^+$ in CH_3CN at 20 ℃ (Stopped-flow, λ = 430 nm).

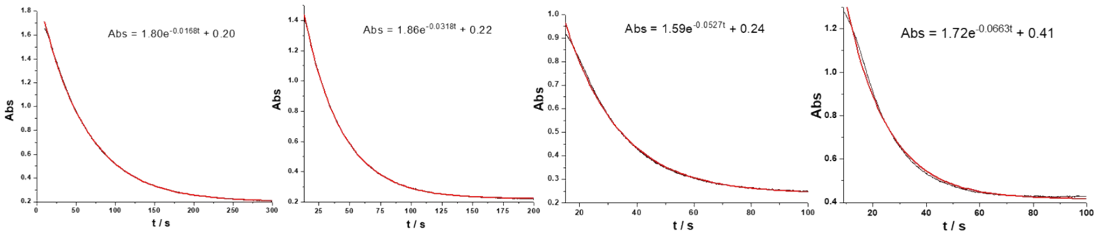

$[\mathbf{A1}^+]$ / M	$[\mathbf{1a}]$ / M	k_{obs} / s^{-1}
≈ 5.00×10^{-5}	3.03×10^{-3}	1.68×10^{-2}
≈ 5.00×10^{-5}	5.06×10^{-3}	3.18×10^{-2}
≈ 5.00×10^{-5}	8.27×10^{-3}	5.27×10^{-2}
≈ 5.00×10^{-5}	1.14×10^{-2}	6.63×10^{-2}
$k_{HT} = 5.94 \pm 0.46$ $M^{-1}s^{-1}$		

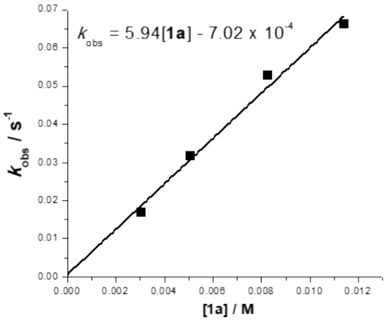

Table S2. Kinetics of the reaction of **1a-D** with $\mathbf{A1}^+$ in CH_3CN at 20 ℃ (Stopped-flow, λ = 430 nm).

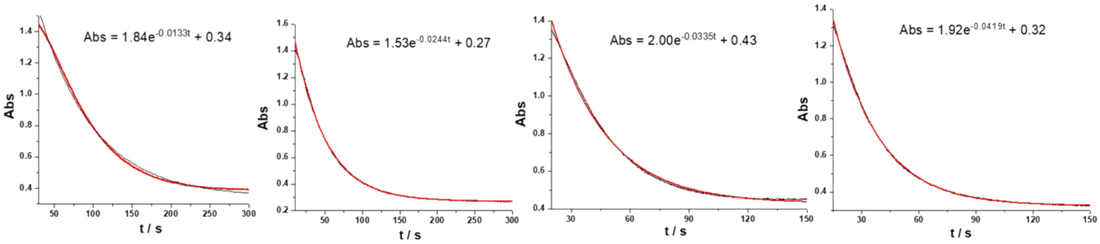

$[\mathbf{A1}^+]$ / M	$[\mathbf{1a\text{-}D}]$ / M	k_{obs} / s^{-1}
≈ 5.00×10^{-5}	7.33×10^{-3}	1.33×10^{-2}
≈ 5.00×10^{-5}	1.14×10^{-2}	2.44×10^{-2}
≈ 5.00×10^{-5}	1.51×10^{-2}	3.35×10^{-2}
≈ 5.00×10^{-5}	1.96×10^{-2}	4.19×10^{-2}
$k_{HT} = 2.34 \pm 0.15$ $M^{-1}s^{-1}$		

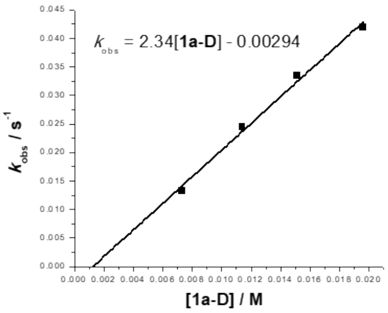

6. Kinetics for the reactions of 1a and 1a-D with 2,4,6-tri-tert-butylphenol $O^{\bullet}$.

Table S3. Kinetics of the reaction of **1a** with $\mathbf{O}^{\bullet}$ in CH_3CN at 20 °C (Stopped-flow, λ = 630 nm).

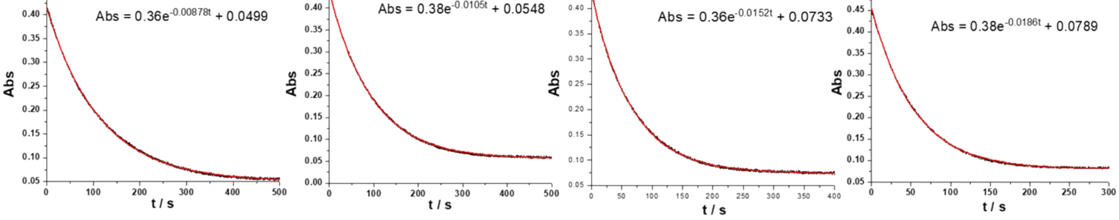

[O•]/ M	[**1a**] / M	k_{obs} / s^{-1}
≈ 2.00 × 10^{-4}	5.74 × 10^{-3}	8.78 × 10^{-3}
≈ 2.00 × 10^{-4}	7.81 × 10^{-2}	1.05 × 10^{-2}
≈ 2.00 × 10^{-4}	1.05 × 10^{-2}	1.52 × 10^{-2}
≈ 2.00 × 10^{-4}	1.31 × 10^{-2}	1.86 × 10^{-2}
$2k_{HAT}$ = 1.39 ± 0.11 $M^{-1}s^{-1}$		

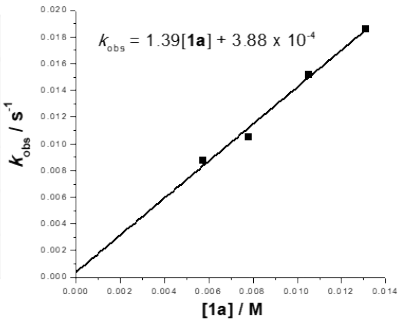

Table S4. Kinetics of the reaction of **1a-D** with **O•** in CH_3CN at 20 ℃ (Stopped-flow, λ = 630 nm).

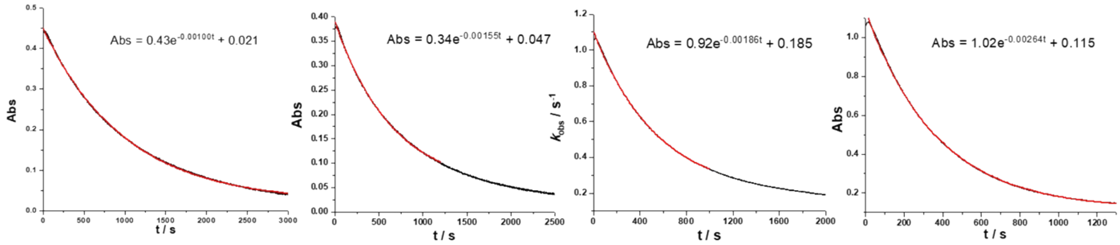

[O•]/ M	[**1a-D**] / M	k_{obs} / s^{-1}
≈ 2.00 × 10^{-4}	2.65 × 10^{-2}	1.00 × 10^{-3}
≈ 2.00 × 10^{-4}	3.97 × 10^{-2}	1.55 × 10^{-3}
≈ 5.00 × 10^{-4}	4.76 × 10^{-2}	1.86 × 10^{-3}
≈ 5.00 × 10^{-4}	6.30 × 10^{-2}	2.64 × 10^{-3}
$2k_{HAT}$ = 0.0448 ± 0.0020 $M^{-1}s^{-1}$		

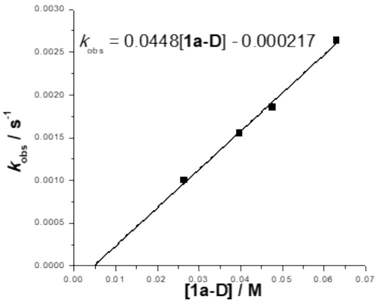

Table S5. Kinetics of the reaction of **1b** with **O•** in CH_3CN at 20 ℃ (Stopped-flow, λ = 630 nm).

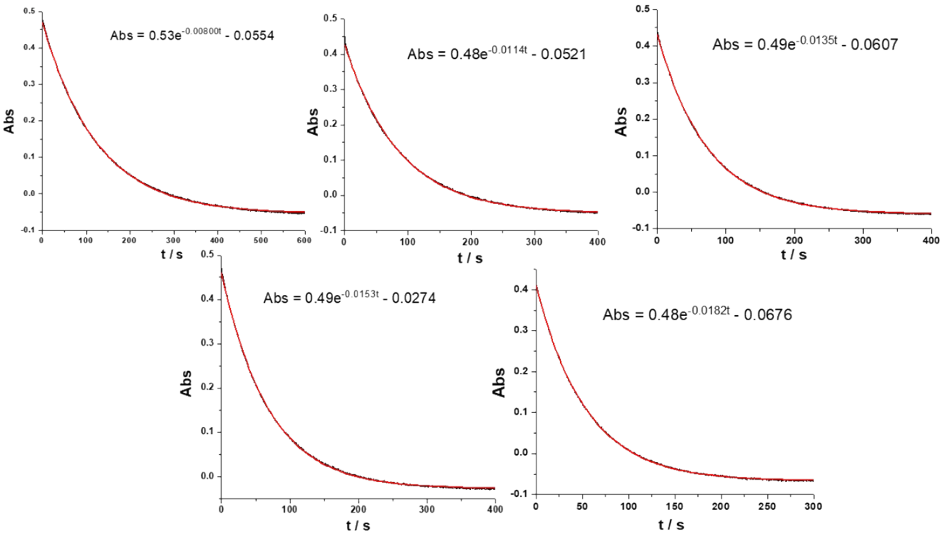

[O•]/ M	[**1b**] / M	k_{obs} / s^{-1}
≈ 2.00 × 10^{-4}	6.69 × 10^{-3}	8.00 × 10^{-3}
≈ 2.00 × 10^{-4}	8.62 × 10^{-3}	1.14 × 10^{-2}
≈ 2.00 × 10^{-4}	1.00 × 10^{-2}	1.35 × 10^{-2}
≈ 2.00 × 10^{-4}	1.15 × 10^{-2}	1.53 × 10^{-2}
≈ 2.00 × 10^{-4}	1.35 × 10^{-2}	1.82 × 10^{-2}
$2k_{HAT}$ = 1.47 ± 0.059 M^{-1}s^{-1}		

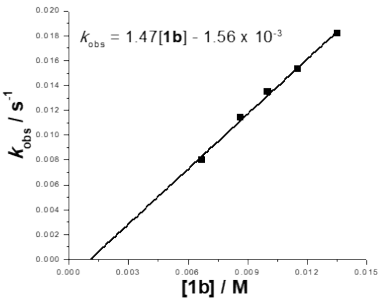

Table S6. Kinetics of the reaction of **1b-D** with **O•** in CH_3CN at 20 ℃ (Stopped-flow, λ = 630 nm).

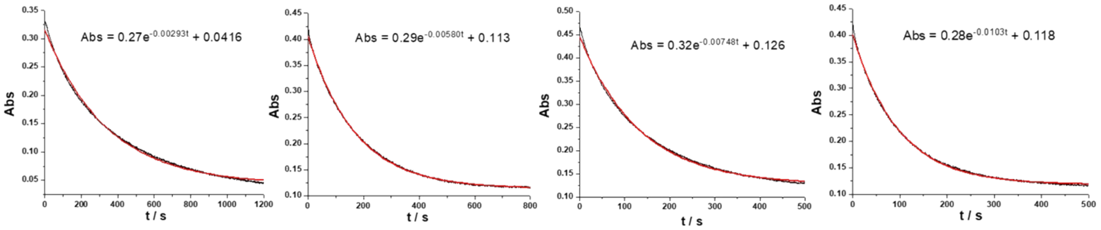

[O•]/ M	[**1b-D**] / M	k_{obs} / s^{-1}
≈ 2.00 × 10^{-4}	1.04 × 10^{-2}	2.93 × 10^{-3}
≈ 2.00 × 10^{-4}	2.07 × 10^{-2}	5.80 × 10^{-3}
≈ 2.00 × 10^{-4}	2.80 × 10^{-2}	7.48 × 10^{-3}
≈ 2.00 × 10^{-4}	4.12 × 10^{-2}	1.03 × 10^{-2}
$2k_{HAT}$ = 0.238 ± 0.012 M^{-1}s^{-1}		

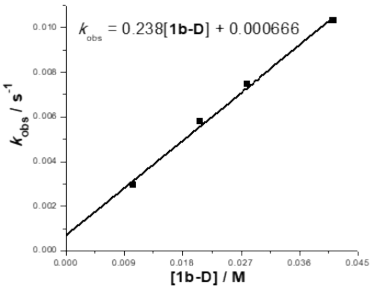

7. Kinetics for the reactions of 1a and 1a-D with 2,4,6-tri-tert-butylphenol O• at 292 K – 320 K.

Table S7. Kinetics of the reaction of **1a** with **O•** in CH_3CN at different temperatures (Stopped-flow, λ = 630 nm).

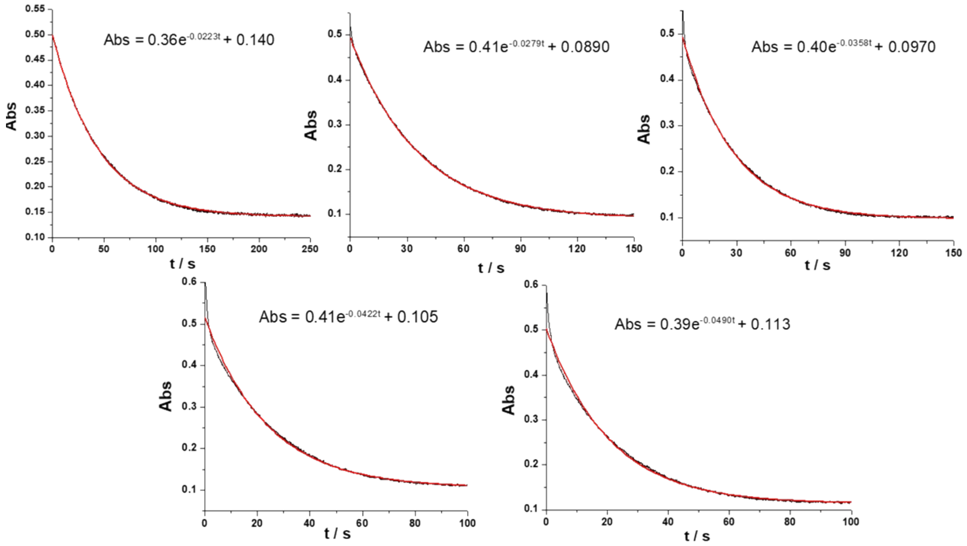

[**1a**] = 0.0110 M					
T / K	292.9	300.1	305.8	312	320
1/T	0.003414	0.003332	0.00327	0.003205	0.003125
k_{obs} (s^{-1})	0.0223	0.0279	0.0358	0.0422	0.049
k_{HAT} (**1a**)	1.013636	1.268182	1.627273	1.918182	2.227273
$\ln k_{HAT}$	0.013544	0.237584	0.486905	0.651378	0.800778

Table S8. Kinetics of the reaction of **1a-D** with **O•** in CH_3CN at different temperatures (Stopped-flow, λ = 630 nm).

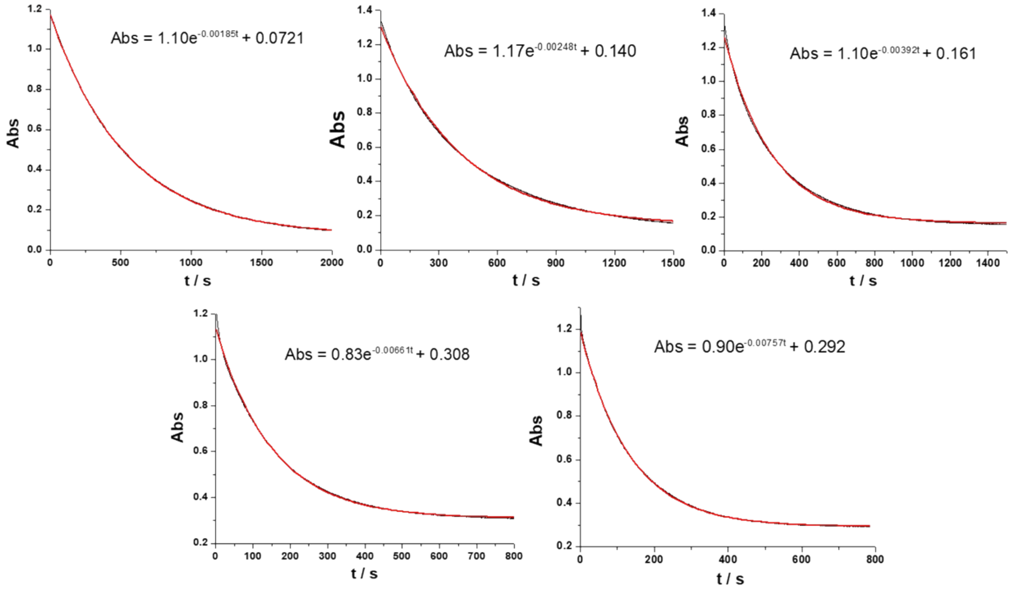

[**1a-D**] = 0.0397 M					
T / K	294.2	301.4	309.3	316.8	322
1/T	0.003399	0.003318	0.003233	0.003157	0.003106
k_{obs} (s^{-1})	0.00185	0.00248	0.00392	0.00661	0.00757
k_{HAT} (**1a-D**)	0.0233	0.031234	0.04937	0.083249	0.09534
$\ln k_{HAT}$	-3.75931	-3.46624	-3.00841	-2.48591	-2.35031

8. Kinetics for the reactions of 1b and 1b-D with 2,4,6-tri-tert-butylphenol O• at 292 K – 322 K.

Table S9. Kinetics of the reaction of **1b** with **O•** in CH_3CN at different temperatures (Stopped-flow, λ = 630 nm).

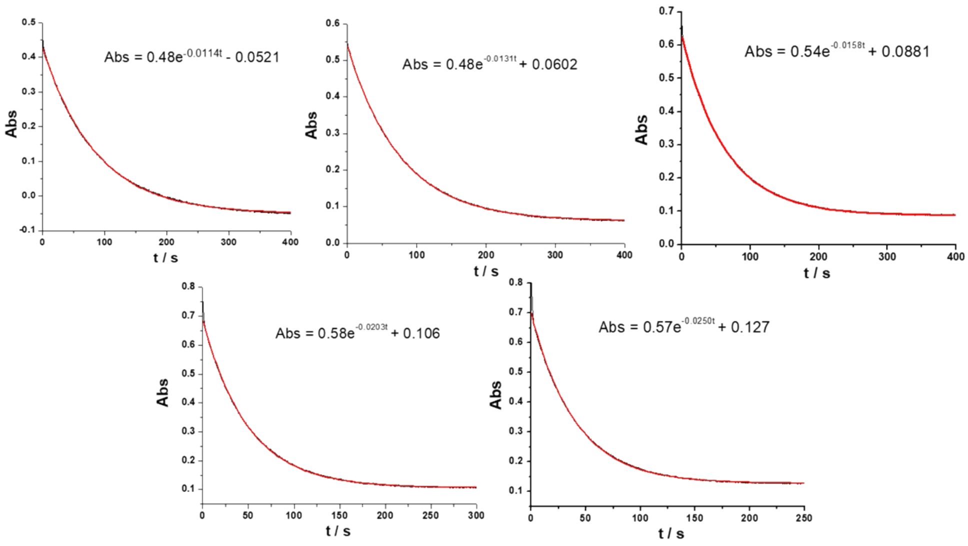

[**1b**] = 0.00862 M					
T / K	293	298	303	310.5	317.5
1/T	0.003413	0.003356	0.0033	0.003221	0.00315
k_{obs} (s^{-1})	0.0114	0.0131	0.0158	0.0203	0.025
k_{HAT} (**1b**)	0.661253	0.759861	0.916473	1.177494	1.450116
lnk_{HAT}	-0.41362	-0.27462	-0.08722	0.163389	0.371644

Table S10. Kinetics of the reaction of **1b-D** with **O$^{•}$** in CH_3CN at different temperatures (Stopped-flow, λ = 630 nm).

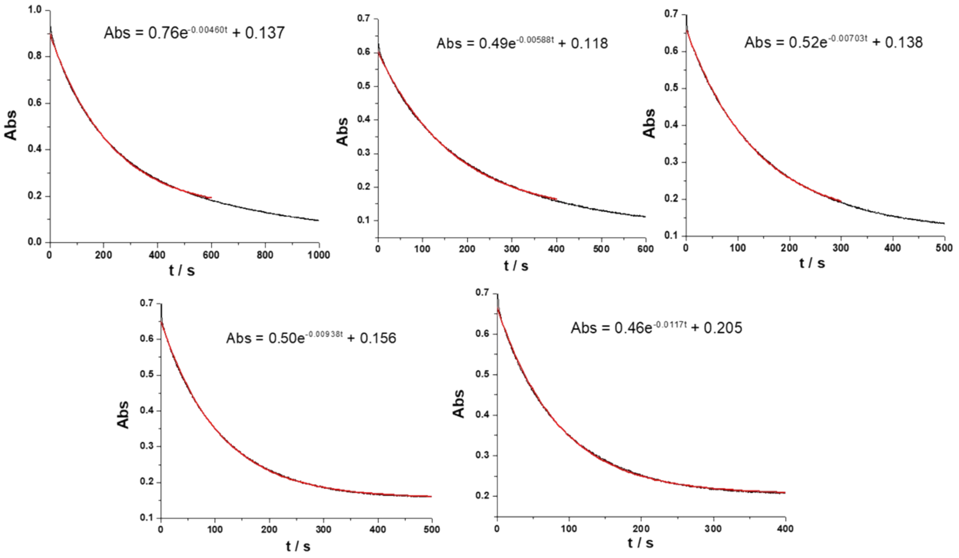

$[\mathbf{1b\text{-}D}] = 0.0207$ M					
T / K	295	303	309.4	317.2	322
1/T	0.00339	0.0033	0.003232	0.003153	0.003106
k_{obs} (s^{-1})	0.0046	0.00588	0.00703	0.00938	0.0117
k_{HAT} (**1b-D**)	0.111111	0.142029	0.169807	0.22657	0.282609
$\ln k_{HAT}$	-2.19722	-1.95172	-1.77309	-1.4847	-1.26369

9. Kinetics for the reactions of 1b and 1b-D with 2,4,6-tri-tert-butylphenol O• at 292 K – 322 K.

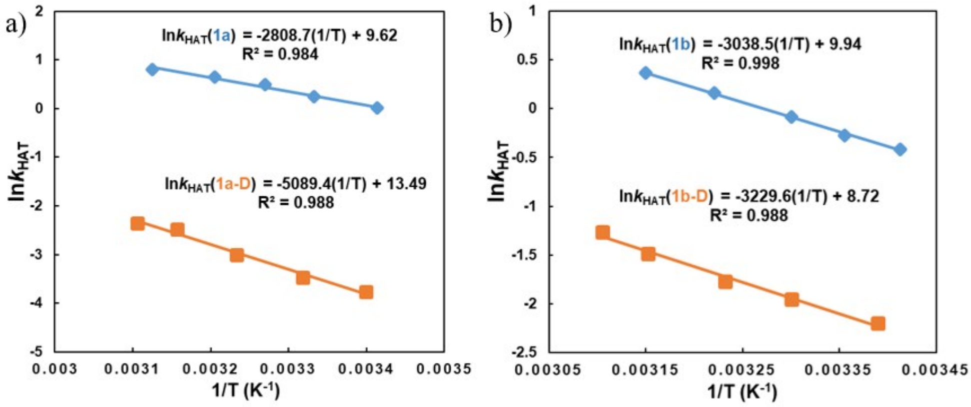

Figure S2. The plots of $\ln k_{HAT}$ vs 1/T for a) the reactions of **O•** with **1a** (blue diamonds) and **1a-D** (orange squares); b) the reactions of **O•** with **1b** (blue diamonds) and **1b-D** (orange squares).

Table S11. Eyring and Arrhenius parameters for hydrogen transfers from **1a**, **1a-D**, **1b** and **1b-D** to **O•** in acetonitrile at 293 K.

	$\Delta H^{\ddagger}$ [a]	$\Delta S^{\ddagger}$ [d]	$\Delta G^{\ddagger}$ [c]	E_a [d]	$\ln A$
**1a**	4.97	-41.45	17.11	5.58	9.62
**1a-D**	9.50	-33.78	19.40	10.11	13.49
**1b**	5.43	-40.81	17.39	6.04	9.94
**1b-D**	5.81	-43.26	18.49	6.42	8.72

[a] In units of kcal/mol. [b] In units of cal/mol/K. [c] At 293 K, in units of kcal/mol. [d] In units of kcal/mol.

10. The reactions of 1a and 1a-D with *N*-methylacridinium ion A1⁺ in CD_3CN.

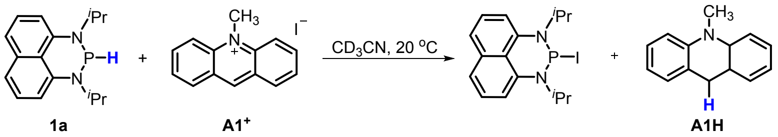

A1⁺ (0.02 mmol) was added into the CD_3CN (0.5 mL) solution of **1a** (0.02 mmol), and the

mixture was checked by NMR spectrum after 10 minutes.

(1) ^{1}H NMR in CD_3CN

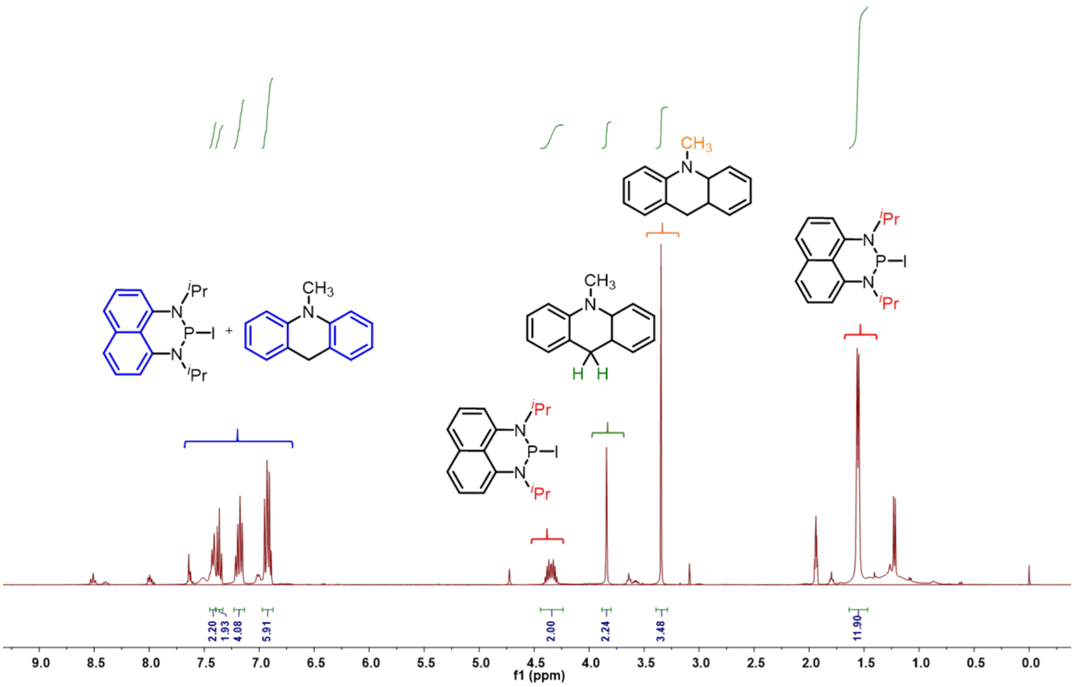

(2) ^{31}P NMR in CD_3CN

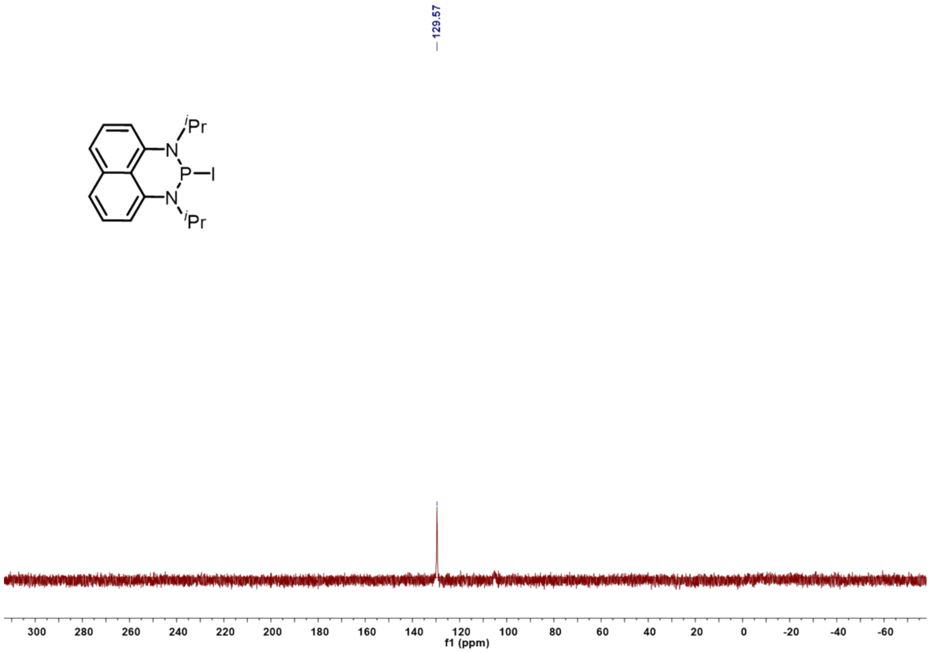

Figure S3. ^{1}H and ^{31}P NMR spectra comparison for the stoichiometric reaction between **1a** and **A1**$^+$.

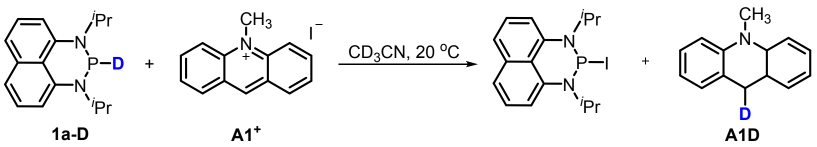

A1$^+$ (0.02 mmol) was added into the CD_3CN (0.5 mL) solution of **1a-D (**0.02 mmol**)**, and the mixture was checked by NMR spectra after 10 minutes.

(1) ^1H NMR in CD_3CN

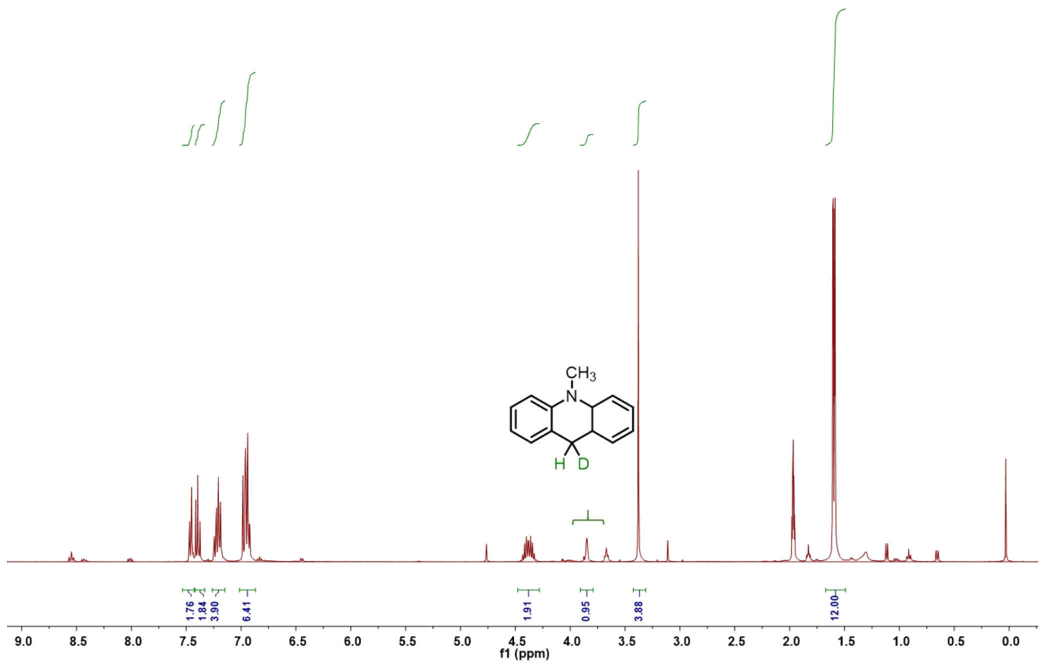

(2) ^{31}P NMR in CD_3CN

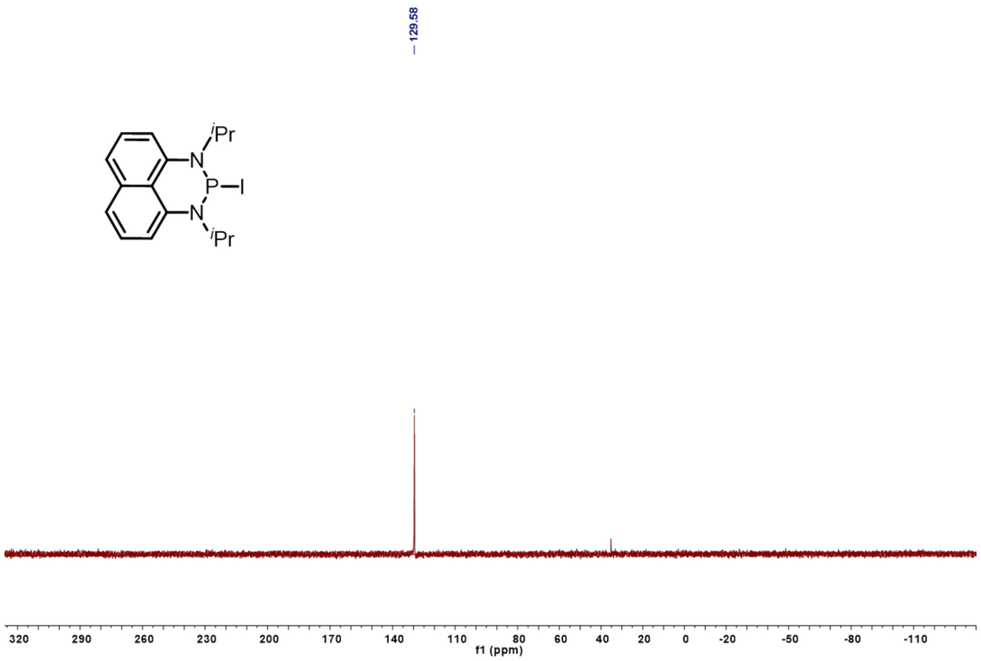

Figure S4. ^{1}H and ^{31}P NMR spectra comparison for the stoichiometric reaction between **1a-D** and **A1**$^+$. Deuterated ratio > 95% based on the analysis of ^{1}H NMR.

11. The equilibrium between 1a and A2$^+$ in CD$_3$CN.

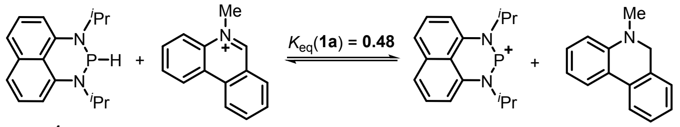

1a **A2$^+$** **1a-[P]$^+$** **A2H**

Equilibrium for **1a** (0.013 mmol) and **A2**$^+$ (0.013 mmol) was established about 24 hours in acetonitrile-d_3 (0.5 mL) at 20 ^{o}C. All the four components in the mixture can be well monitored by ^{1}H NMR and ^{31}P NMR, albeit slight oxidative deterioration of **1a**. The equilibrium constant was obtained by the concentration ratio of corresponding four components and calculated by the following equation:

$$K_{eq}(\mathbf{1a}) = \frac{[\mathbf{1a\text{-}[P]^+}][\mathbf{A2H}]}{[\mathbf{1a}][\mathbf{A2^+}]} = 0.48$$

$$\Delta G_{rxn} = -RT\ln K_{eq} = 0.43 \text{ kcal/mol}$$

$$\Delta G_{rxn} = \Delta G_{H^-}(\mathbf{1a}) - \Delta G_{H^-}(\mathbf{A2H})$$

Referenced to the hydricity of 5-methyl-5,6-dihydrophenanthridine **A2H** (ΔG_{H^-}(**A2H**) = 61.4 kcal/mol), the hydricity ΔG_{H^-} of **1a** can be obtained as 61.8 kcal/mol.

(1) ^{1}H NMR in CD$_3$CN

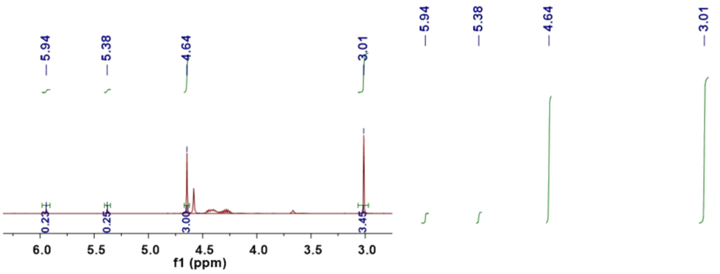

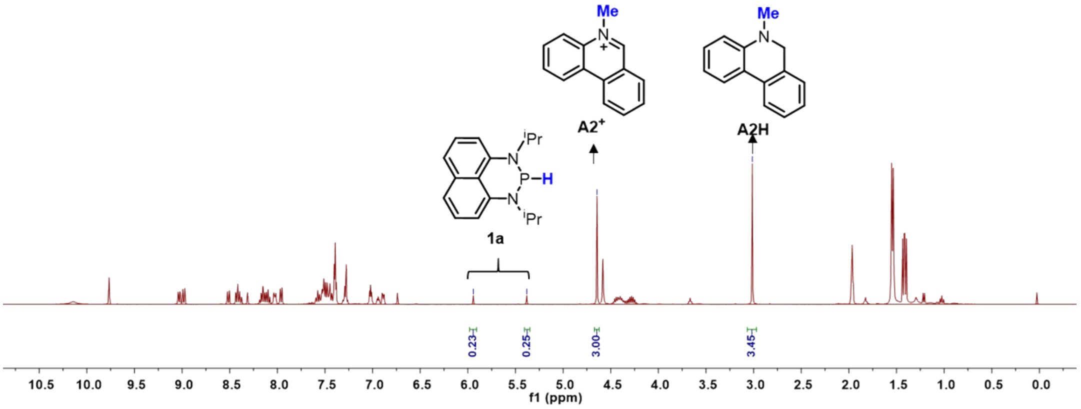

(2) ^{31}P NMR in CD$_3$CN

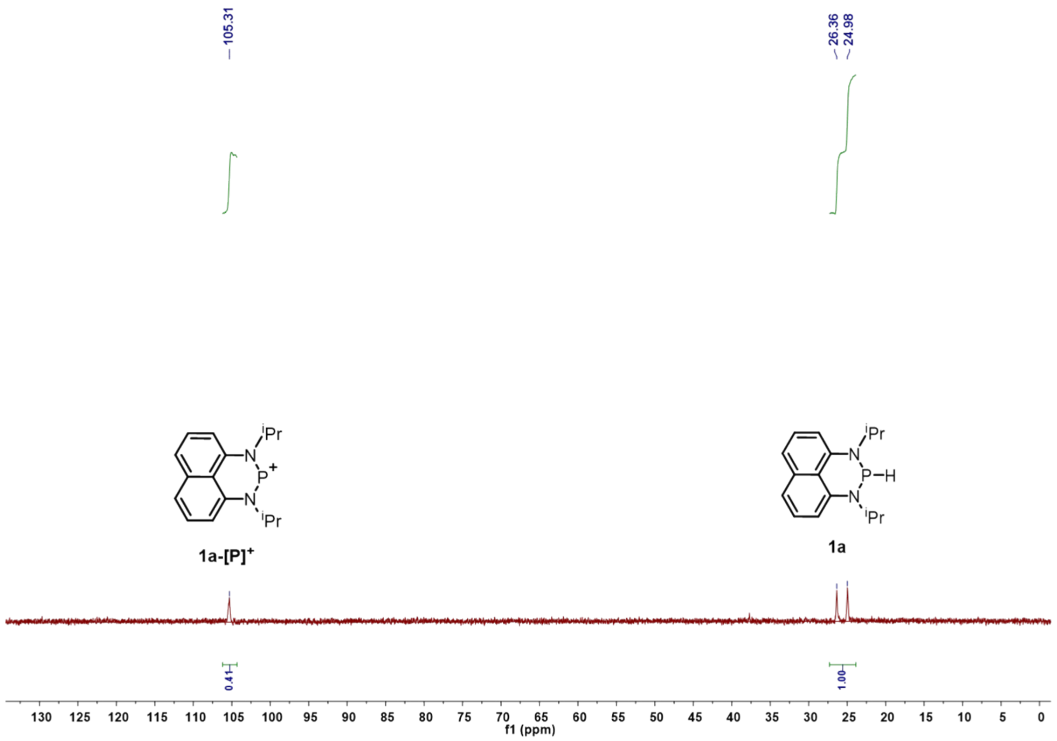

Figure S5. Construction of equilibrium for the reaction of **1a** (0.013 mmol) and **A2**$^+$ (0.013 mmol) in CD_3CN at 20 °C.

A. The verification of the hydricity ΔG_{H^-} of 1a by the reaction of 1a-[P]$^+$ with A2H:

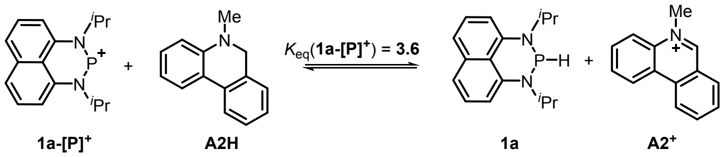

Equilibrium for **1a-[P]**$^+$ (0.01 mmol) and **A2H** (0.015 mmol) was established in about 24 hours in acetonitrile-d_3 (0.5 mL) at 20 °C. All the four components in the mixture can be well monitored by ^{1}H NMR and ^{31}P NMR, albeit slight deterioration. The equilibrium constant was obtained from the concentration ratio of the corresponding four components by using the following equation:

$$K_{eq}(\mathbf{1a\text{-}[P]^+}) = \frac{[\mathbf{1a}][\mathbf{A2^+}]}{[\mathbf{1a\text{-}[P]^+}][\mathbf{A2H}]} = 3.6$$

$$\Delta G_{rxn} = -RT\ln K_{eq} = -0.75 \text{ kcal/mol}$$

$$\Delta G_{rxn} = \Delta G_{H^-}(\mathbf{A2H}) - \Delta G_{H^-}(\mathbf{1a})$$

Referenced to the hydricity of 5-methyl-5,6-dihydrophenanthridine **A2H** (ΔG_{H^-}(**A2H**) = 61.4 kcal/mol), the hydricity ΔG_{H^-} of **1a** can be obtained as 62.1_5 kcal/mol.

(1) ^{1}H NMR in CD_3CN

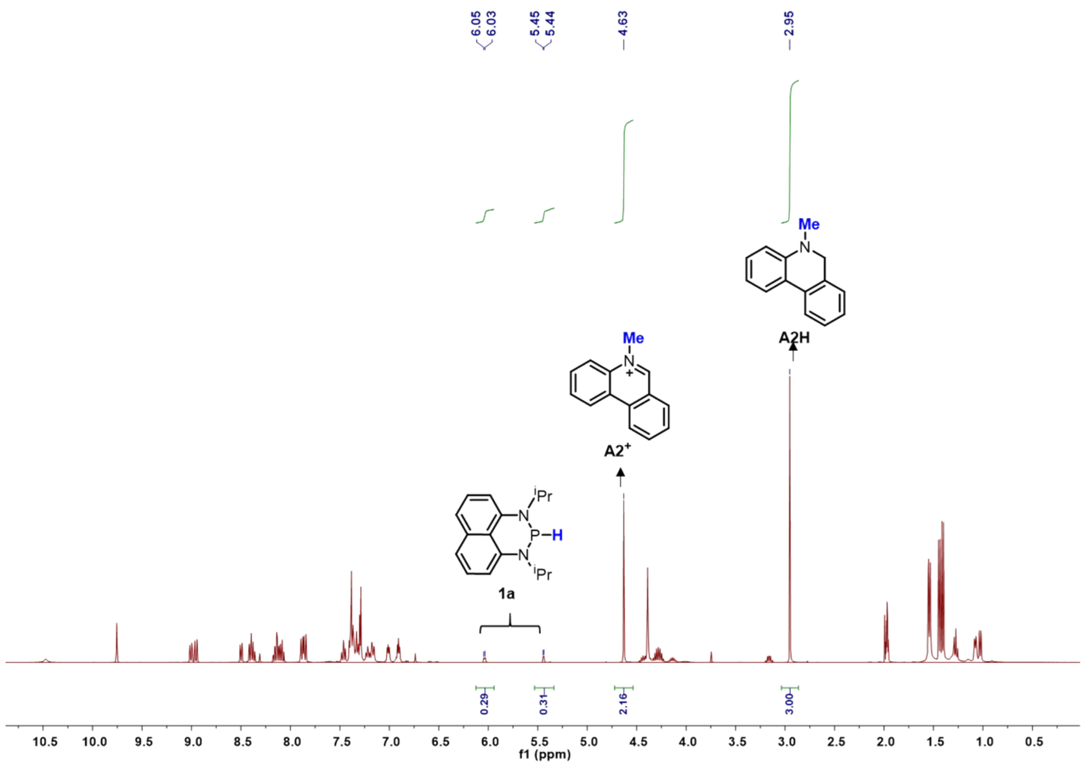

(2) ^{31}P NMR in CD_3CN

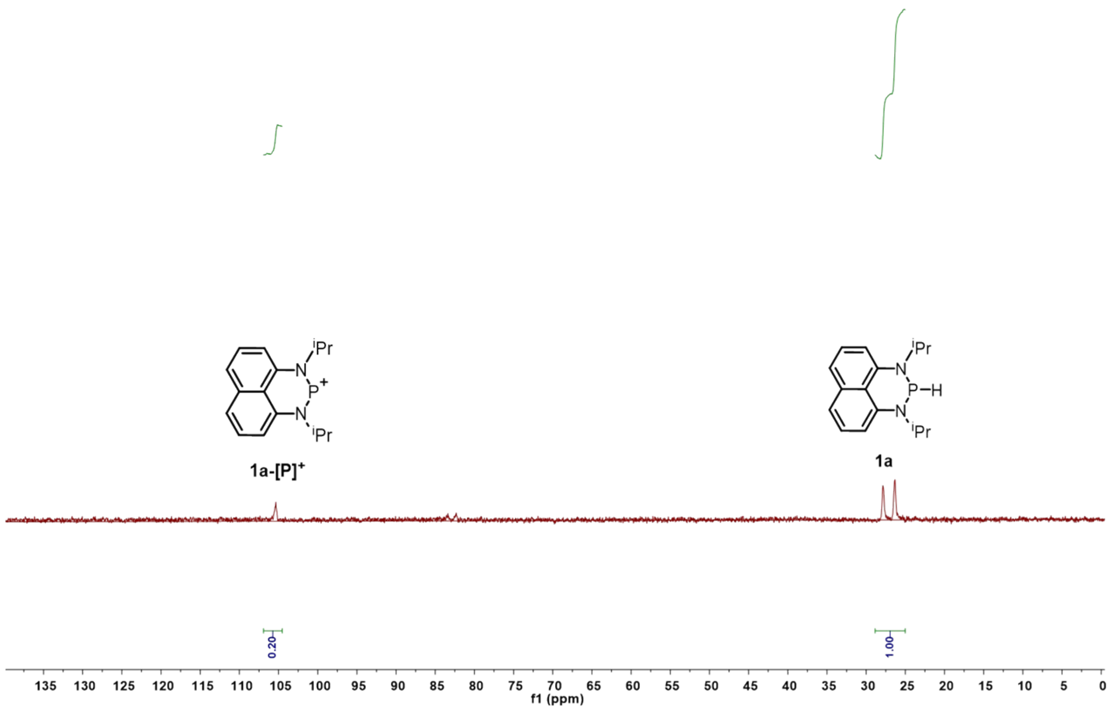

Figure S6. The equilibrium for the reaction of **1a-[P]**$^+$ (0.015 mmol) with **A2H** (0.015 mmol) in CD_3CN at 20 ^oC.

B. The verification of the hydricity ΔG_H- of 1a by the reaction of 1a with A4$^+$:

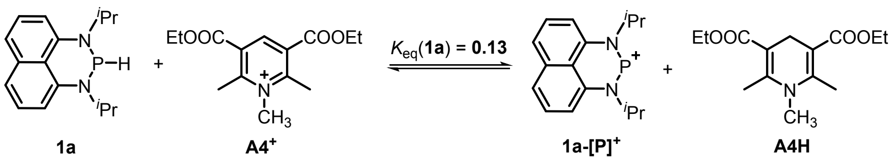

Equilibrium for **1a** (0.015 mmol) and **A4**$^+$ (0.015 mmol) was established in about 24 hours in acetonitrile-d_3 (0.5 mL) at 20 °C. All the four components in the mixture can be well monitored by ^{1}H NMR and ^{31}P NMR, albeit slight deterioration. The equilibrium constant was obtained by the concentration ratio of their corresponding four components by using the following equation:

$$K_{eq}(\mathbf{1a}) = \frac{[\mathbf{1a\text{-}[P]^+}][\mathbf{A4H}]}{[\mathbf{1a}][\mathbf{A4^+}]} = 0.13$$

$$\Delta G_{rxn} = -RT\ln K_{eq} = 1.2 \text{ kcal/mol}$$

$$\Delta G_{rxn} = \Delta G_{H^-}(\mathbf{1a}) - \Delta G_{H^-}(\mathbf{A4H})$$

Reference to the hydricity of diethyl 1,2,6-trimethyl-1,4-dihydropyridine-3,5-dicarboxylate **A4H** (ΔG_{H^-}(**A4H**) = 61.5 kcal/mol)[10], the hydricity ΔG_{H^-} of **1a** can be obtained as 62.7 kcal/mol.

(1) ^{1}H NMR in CD_3CN

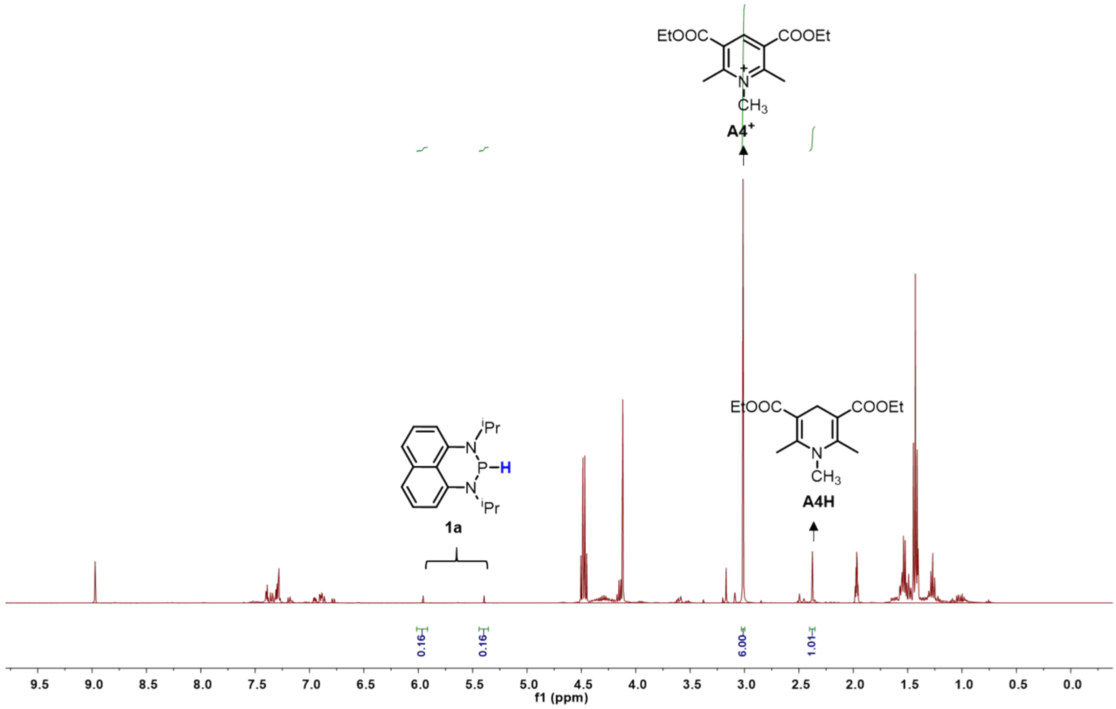

(2) ^{31}P NMR in CD_3CN

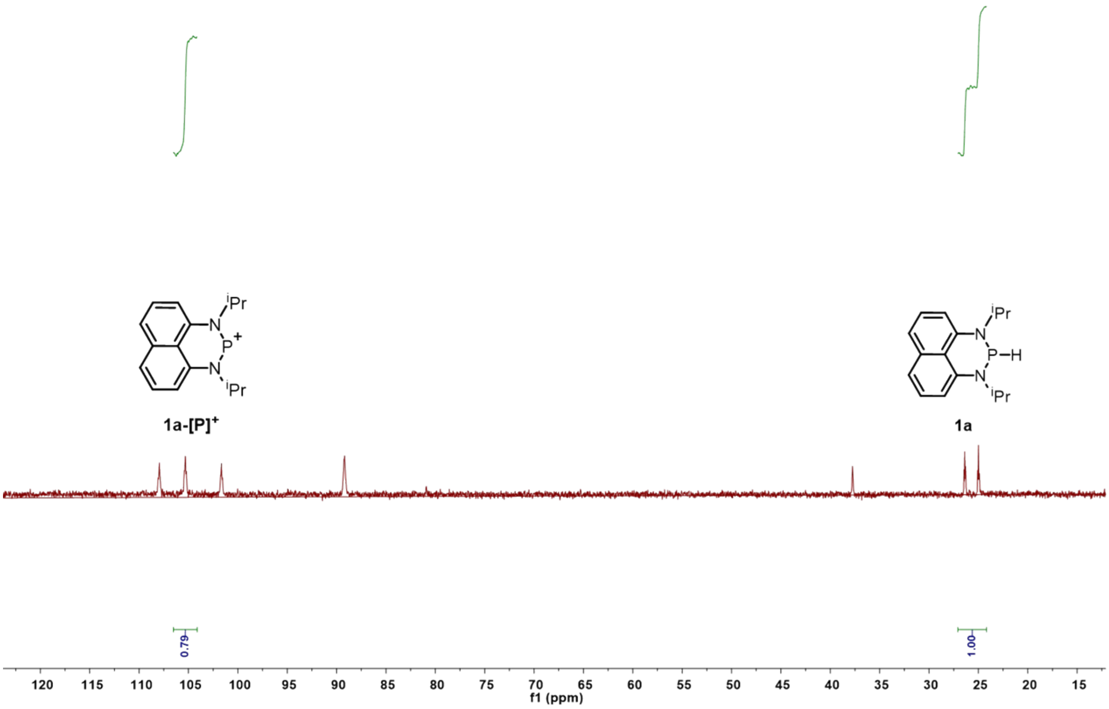

Figure S7. The equilibrium for the reaction of **1a** (0.015 mmol) with **A4**$^+$ (0.015 mmol) in CD_3CN at 20 ^{o}C.

12. The equilibrium between 1b and A3$^+$ in CD_3CN.

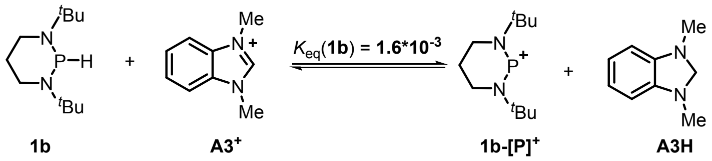

The weak equilibrium for the reaction of **1b** (0.010 mmol) with **A3**$^+$ (0.010 mmol) was established in about 48 hours in acetonitrile-d_3 (0.5 mL) at 20 ^{o}C. The three components (**1b**, **A3**$^+$ and **A3H**) in the mixture can be well monitored by ^{1}H NMR. The **1b-[P]**$^+$ could not be well monitored by ^{1}H NMR due to the overlap of spectrum, but it could be estimated by the concentration of **A3H**. The equilibrium was obtained by the concentration ratio of corresponding four components by using the following equation:

$$K_{eq}(\mathbf{1b}) = \frac{[\mathbf{1b\text{-}[P]^+}][\mathbf{A3H}]}{[\mathbf{1b}][\mathbf{A3^+}]} = 0.0016$$

$$\Delta G_{rxn} = -RT\ln K_{eq} = 3.75 \text{ kcal/mol}$$

$$\Delta G_{rxn} = \Delta G_{H^-}(\mathbf{1b}) - \Delta G_{H^-}(\mathbf{A3H})$$

Reference to the hydricity of 1,3-dimethyl-2,3-dihydro-1*H*-benzo[*d*]imidazole **A3H** (ΔG_{H^-}(**A3H**) = 45.0 kcal/mol), the hydricity ΔG_{H^-} of **1b** was obtained as 48.8.

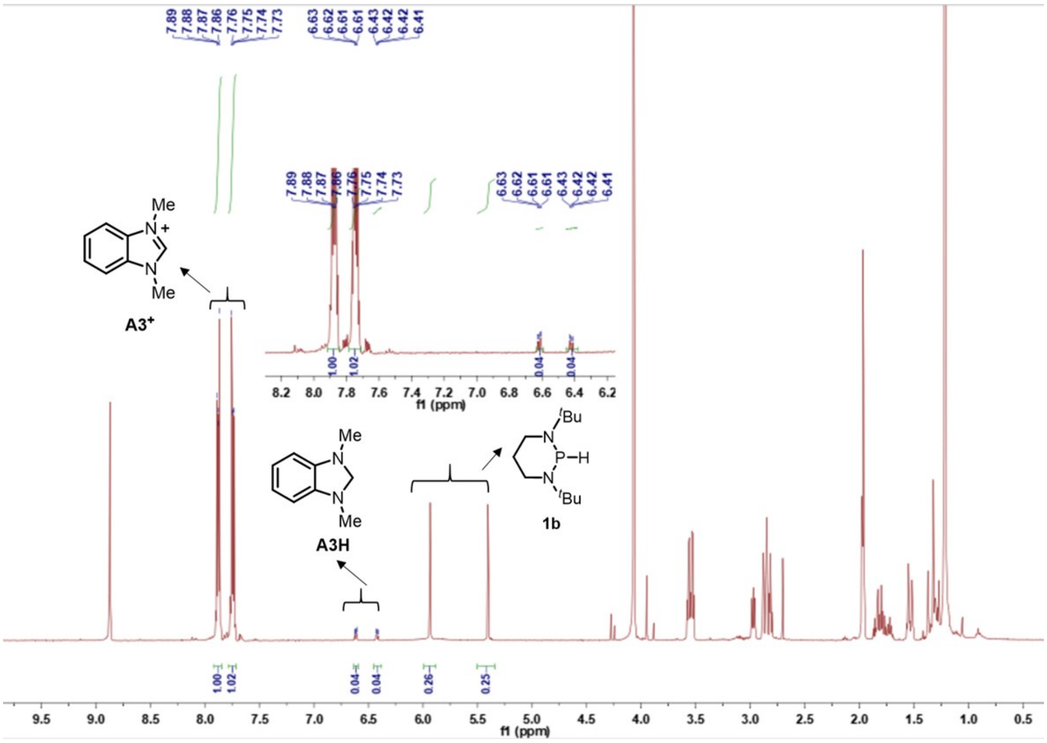

Figure S8. Construction of equilibrium for the reaction of **1b** (0.010 mmol) and **A3**$^+$ (0.010 mmol) in CD_3CN at 20 ^oC.

13. The reactions of 1a with HOTf and HBF_4•Et_2O in CD_3CN.

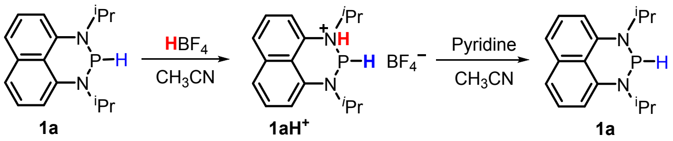

Excess HBF_4•Et_2O was added into the CD_3CN (0.5 mL) solution of **1b** (0.02 mmol), and the mixture was checked by NMR spectrum immediately.

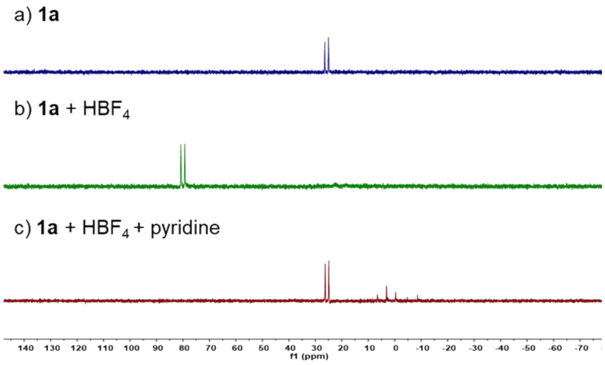

Figure S9. 1) (a) The ^{31}P NMR spectrum of **1a**. (b) The ^{31}P NMR spectrum of **1aH**$^+$ generated by the reaction of HBF_4•Et_2O and **1a** in CD_3CN, which is almost same with generated by HOTf, and the ^{1}H NMR spectrum was not shown here due to the effect of Et_2O. (c) The ^{31}P NMR spectrum of the mixture that adding pyridine to the reaction of **1a** and HBF_4•Et_2O in CD_3CN.

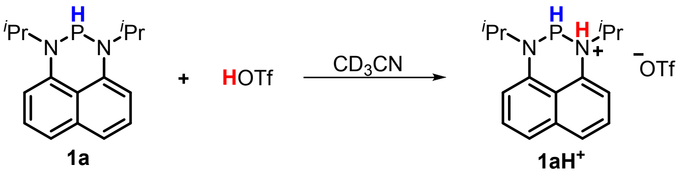

Excess HOTf (purity > 98%) was added into the CD_3CN (0.5 mL) solution of **1b** (0.02 mmol), and the mixture was checked by NMR spectrum immediately.

Analysis: We thought the position of proton is on the nitrogen atom of **1a**, because a doublet peak was detected in the ^{31}P NMR (a triplet peak will be detected if the position of proton is on the phosphorus atom) and the unsymmetrical peaks of naphthyl ring and isopropyl group were also shown in the ^{1}H NMR.

$1aH^+$: **^{1}H NMR** (400 MHz, CD_3CN) δ 8.41 (s, 1H), 8.08 (d, *J* = 8.3 Hz, 1H), 7.77 (d, *J* = 8.2 Hz, 1H), 7.65 (t, *J* = 8.0 Hz, 1H), 7.58 (t, *J* = 7.9 Hz, 1H), 7.49 (d, *J* = 7.9 Hz, 1H), 7.46 (d, *J* = 7.4 Hz, 1H), 6.26 (d, *J* = 236.1 Hz, 1H), 4.58 – 4.49 (m, 1H), 3.87 – 3.51 (m, 1H), 1.68 (d, *J* = 6.5 Hz, 3H), 1.58 (d, *J* = 6.6 Hz, 3H), 1.54 (d, *J* = 6.6 Hz, 3H), 1.06 (d, *J* = 6.5 Hz, 3H). **^{31}P NMR** (162 MHz, CD_3CN) δ 79.12 (d, *J* = 236.2 Hz).

(1) ^{1}H NMR of **$1aH^+$** in CD_3CN

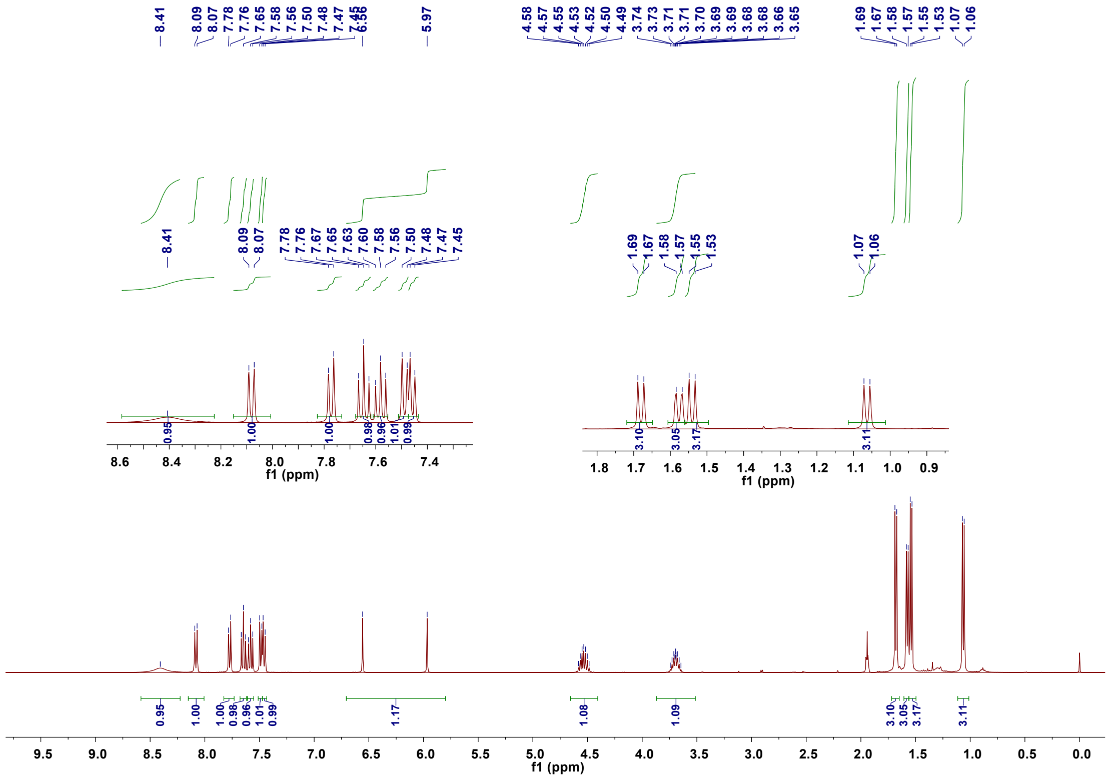

(2) ^{31}P NMR of **$1aH^+$** in CD_3CN

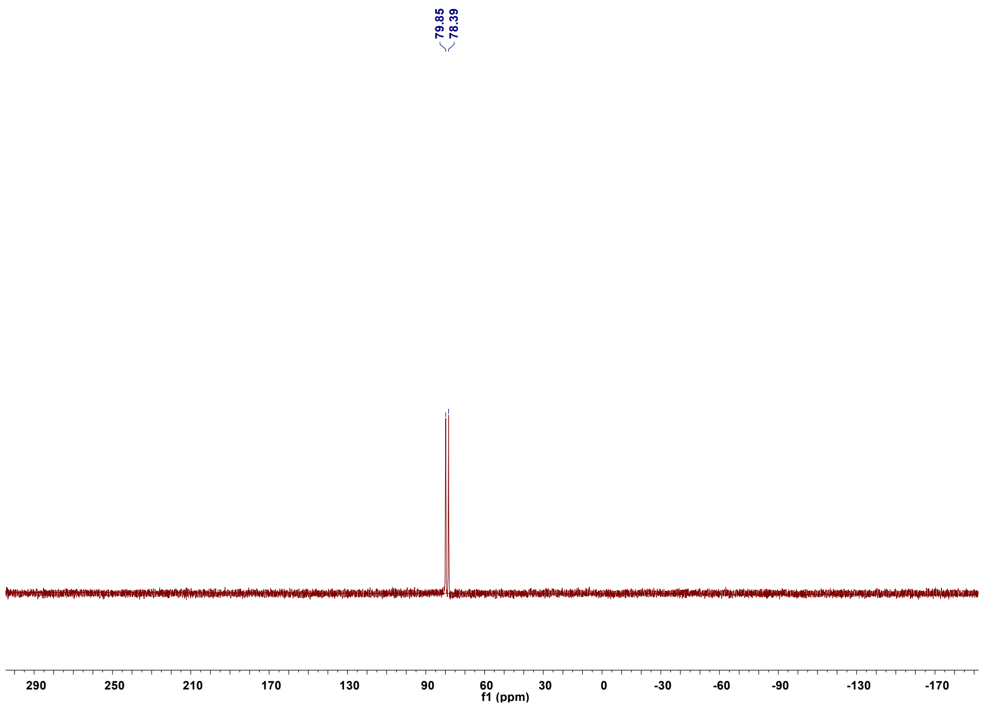

Figure S10. 1) ^{1}H and 2)^{31}P NMR spectra comparison for the reaction between **1a** and HOTf in CD_3CN.

14. The reactions of 1a and 1b with O$^{\bullet}$ in toluene-d_8.

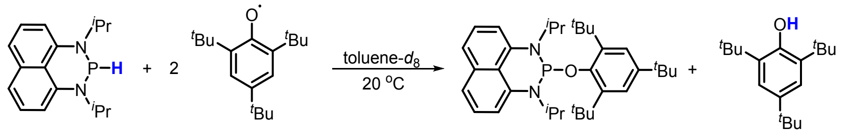

1a O· **OH**

O$^{\bullet}$ (0.02 mmol) was added into the toluene-d_8 (0.5 mL) solution of **1a** (0.01 mmol), and the mixture was checked by NMR spectra after 10 minutes.

(1) ^{1}HNMR in toluene-d_8

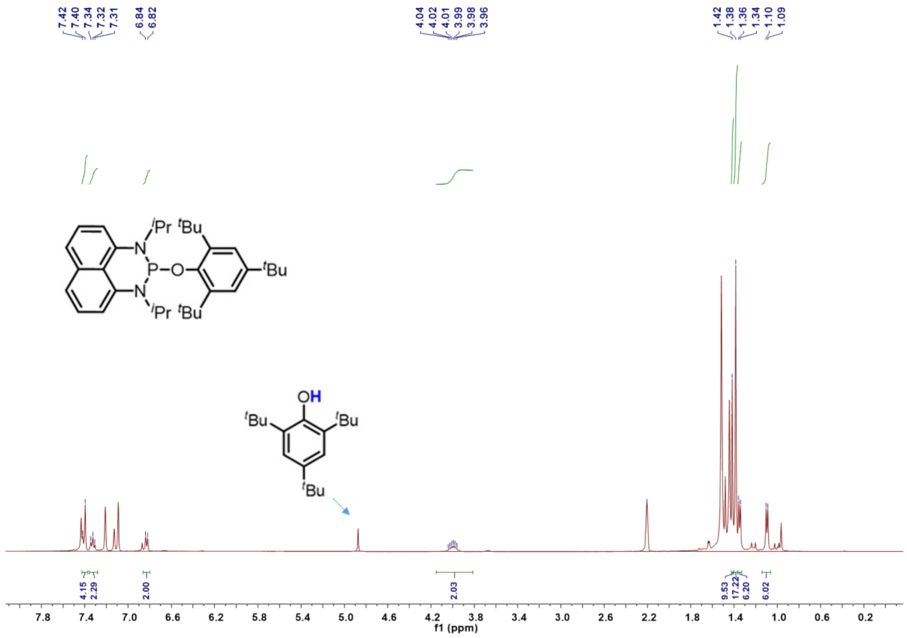

(2) ^{31}P NMR in toluene-d_8

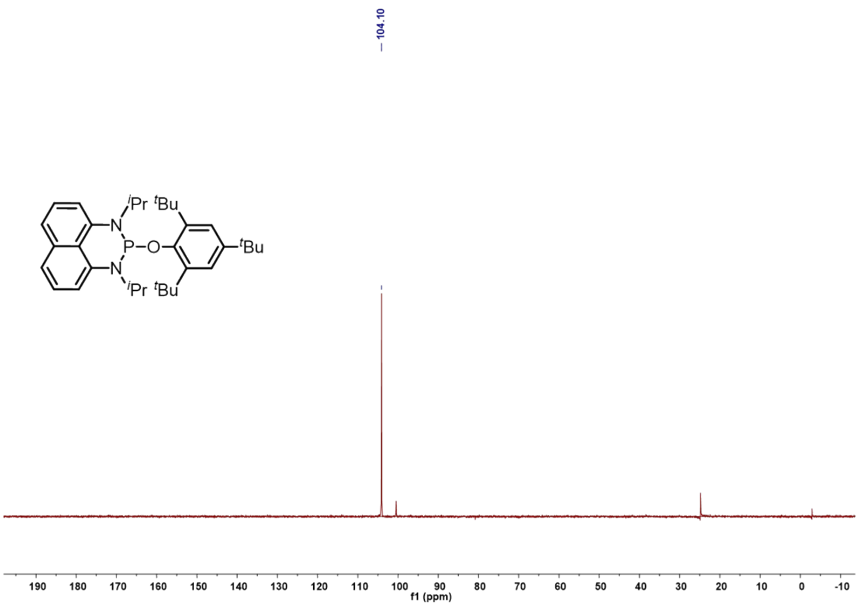

Figure S11. ^{1}H and ^{31}P NMR spectra for the reaction between **1a** and **O•**. The integrations of ^{1}H NMR spectrum are assigned to the structure shown in the spectra, and the shift of 4.76 ppm in ^{1}H NMR spectrum is OH of product 2,4,6-tri-tert-butylphenol, other hydrogen shifts of 2,4,6-tri-tert-butylphenol are not be marked.

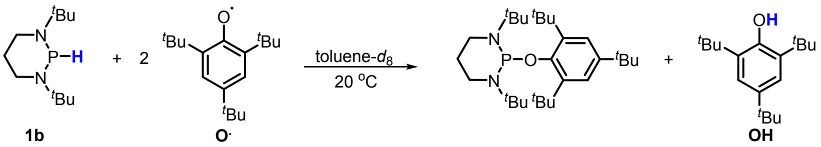

O• (0.02 mmol) was added into the toluene-d_8 (0.5 mL) solution of **1b** (0.01 mmol), and the mixture was checked by NMR spectrum after 10 minutes.

(1) ^{1}H NMR in toluene-d_8

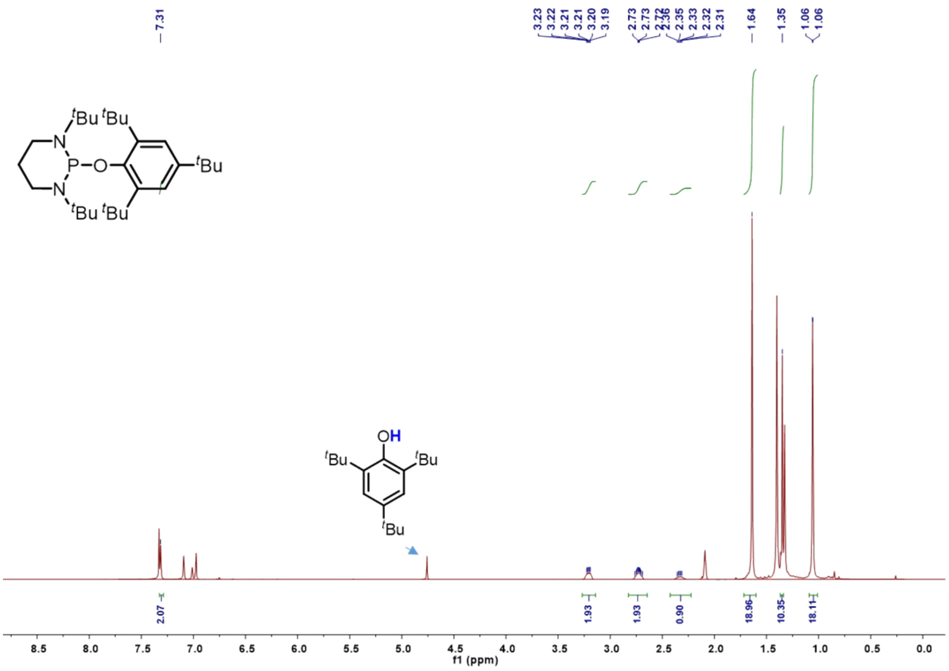

(2) ^{31}P NMR in toluene-d_8

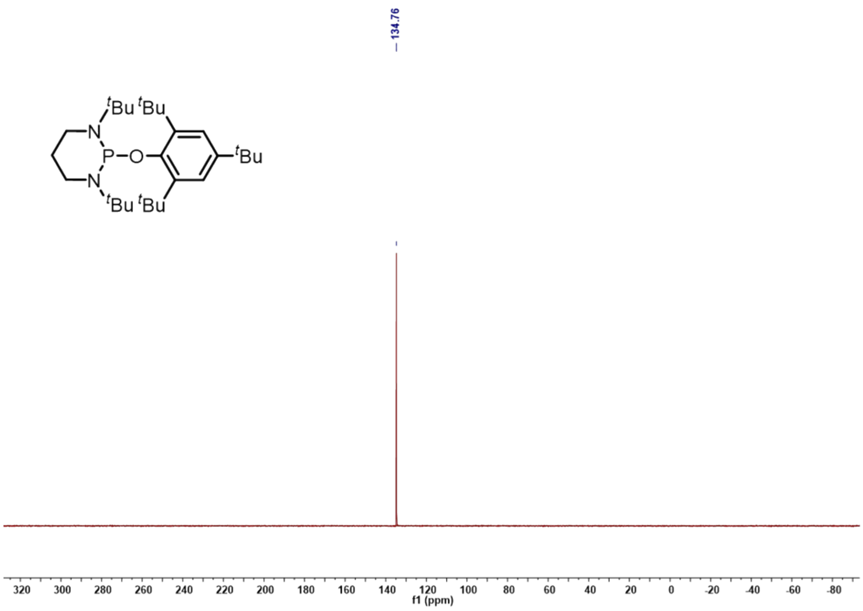

Figure S12. ^{1}H and ^{31}P NMR spectra for the reaction between **1b** and **O•**. The integrations of ^{1}H NMR spectrum are assigned to the structure shown in the spectra, and the shift of 4.76 ppm in ^{1}H NMR spectrum is OH of product 2,4,6-tri-tert-butylphenol **OH**, other hydrogen shifts of 2,4,6-tri-tert-butylphenol are not be marked.

15. The reaction of 1a and 1a-D with ^{t}BuOK.

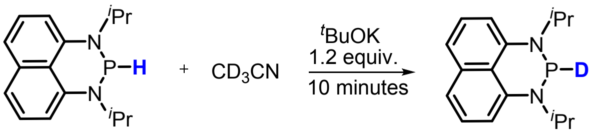

1a **1a-D**

^{t}BuOK (1.2 mmol, 1.2 eq.) was added into CD_3CN (1.0 mL) solution of **1a** (1.0 mmol) at room temperature. After 10 minutes, the solvent was rotary-evaporated, and the residue was extracted with n-hexane (20 mL) and then filtered under Ar atmosphere. The filtrate was evaporated to dryness, producing **1a-D** as a yellowish solid, 265 mg (97%).

However, when the same reaction was performed in the more acidic MeOH solution (with other conditions identical), the P-H species didn't change at all. Similar phenomenon was also observed in the toluene solution.

(1) ^{1}H NMR in CD_3CN

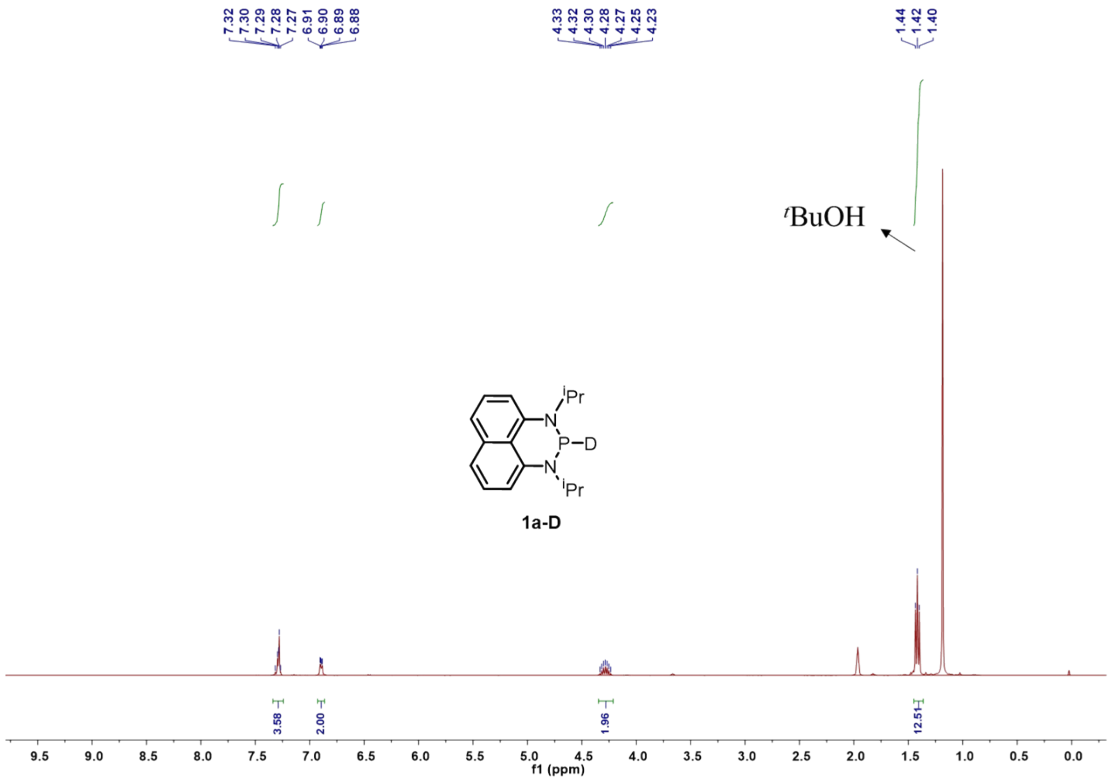

(2) ^{31}P NMR in CD_3CN

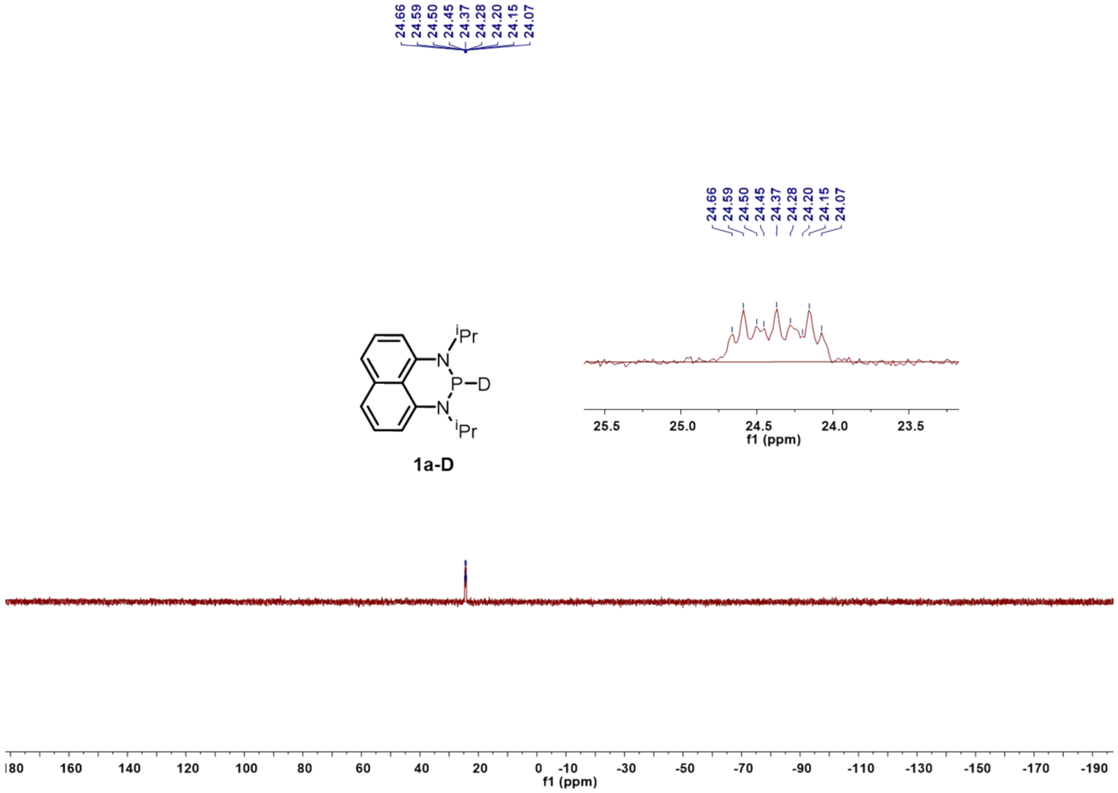

Figure S13. ^{1}H and ^{31}P NMR spectra for the reaction mixture between **1a** and *ᵗ*BuOK in CD_3CN.

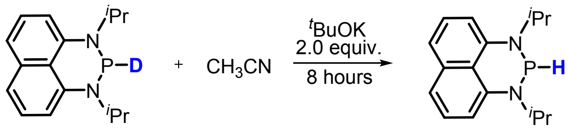

1a-D **1a**

^{t}BuOK (0.04 mmol, 2.0 eq.) was added into the CH_3CN (0.5 mL) solution of **1a** (0.02 mmol) at room temperature, and the mixture was checked by ^{31}P NMR spectra.

^{31}P NMR of the reaction mixture in CH_3CN:

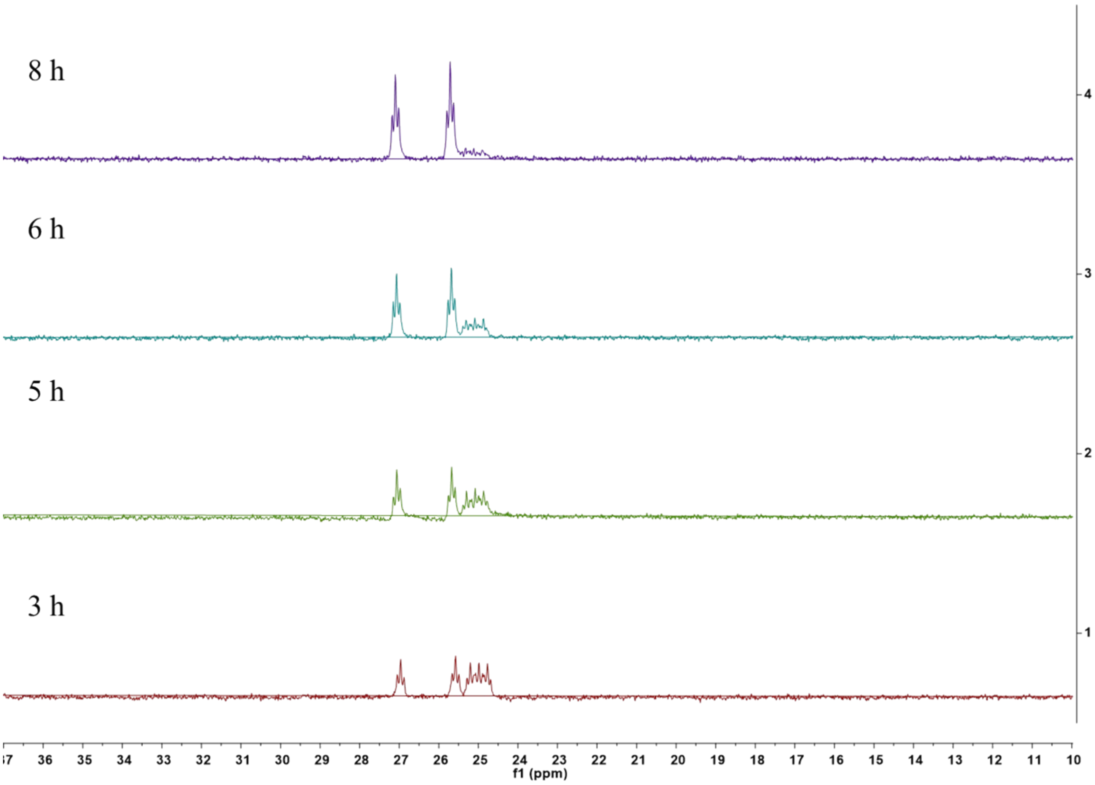

Figure S14. ^{31}P NMR spectra for the reaction mixture between **1a-D** and ^{t}BuOK in CH_3CN.

16. The reaction of 1a-[P]$^+$ with substituted pyridine.

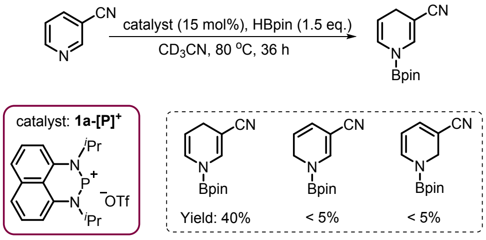

15 mol% load of catalyst and 1.5 equivalents of HBpin were added into the solution of 3-CN-pyridine (2.0 mmol) in 1.0 mL CD_3CN. The mixture was heated to 80 °C for 36 hours. The solvent concentrated in the vacuum. Purification of the residues by flash column chromatography under Ar atmosphere (eluent: n-hexane/CH_3CN: 50/1) gave 1-(4,4,5,5-tetramethyl-1,3,2-dioxaborolan-2-yl)-1,4-dihydropyridine-3-carbonitrile a yellow solid (0.186 g, 40%). And the NMR spectra of mixture and product were shown as below:

^{1}H NMR (400 MHz, CD_3CN) δ 6.86 (d, *J* = 0.9 Hz, 1H), 6.09 (ddd, *J* = 8.3, 3.1, 1.8 Hz, 1H), 4.83 (dt, *J* = 8.2, 3.4 Hz, 1H), 3.03 – 2.95 (m, 2H), 1.25 (s, 12H). **^{13}C NMR** (101 MHz, CD_3CN) δ 141.02, 125.52, 120.48, 104.19, 85.83, 85.26, 24.40, 23.46. **^{11}B NMR** (128 MHz, CD_3CN) δ 23.70.
The NMR spectroscopic data of the product are in good agreement with those in the literature.[11]

(1) ^{1}H NMR of the mixture in CD_3CN

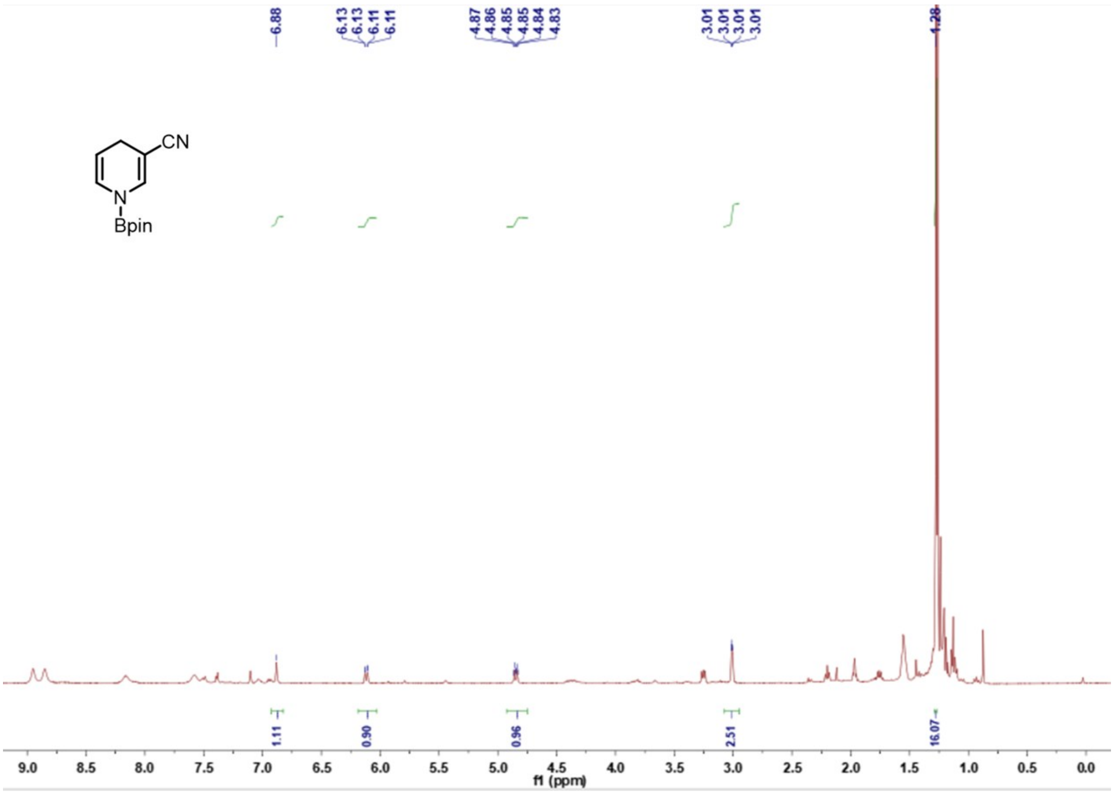

(2) ^{1}H NMR of the isolated product in CD_3CN

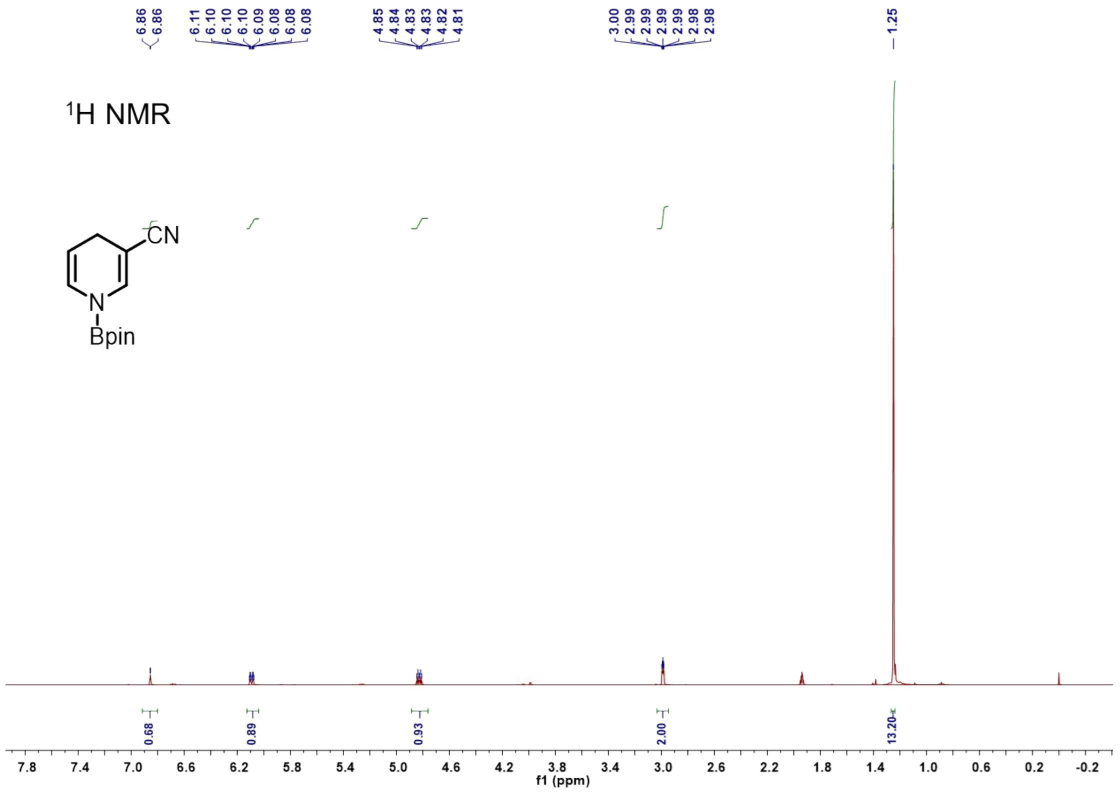

(3) ^{13}C NMR of the isolated product in CD_3CN

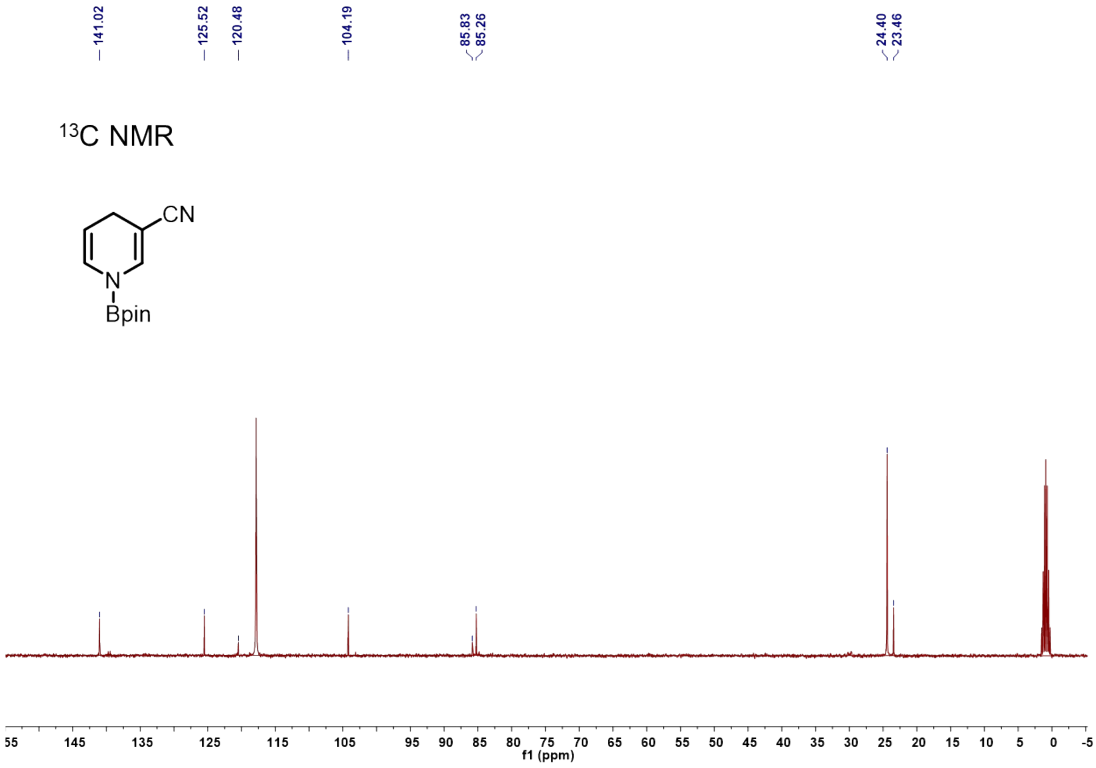

(4) ^{11}B NMR of the isolated product in CD_3CN

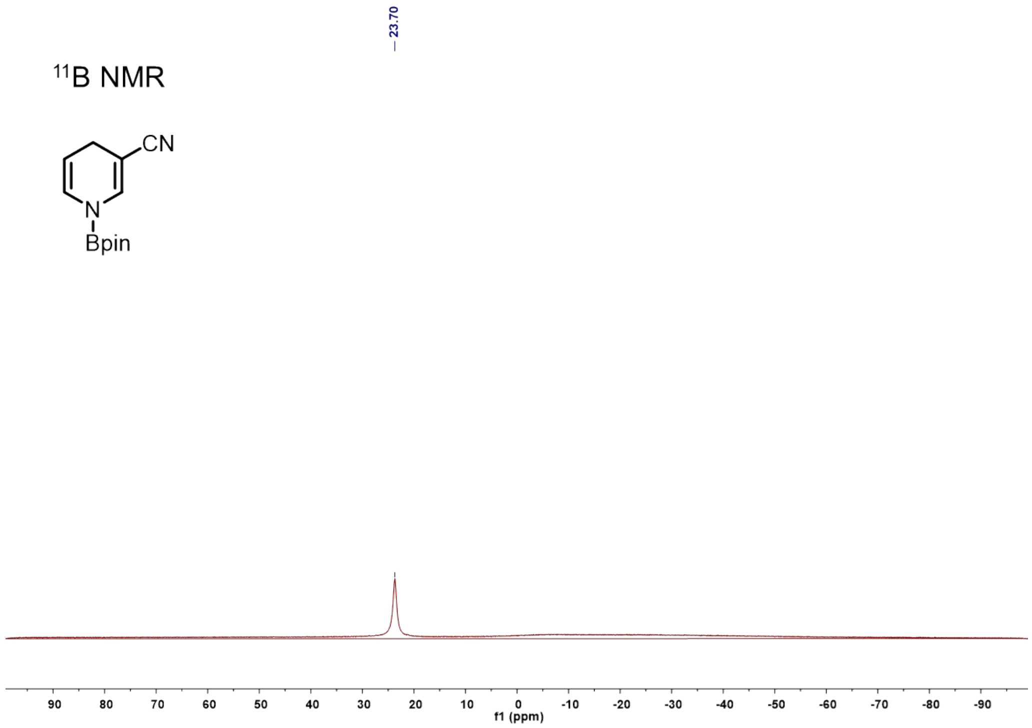

Figure S15. The NMR spectra of the catalytic reaction of **1a-[P]**$^+$ with substituted pyridine.

17. The reactions of 1a and 1b with AIBN in C_6D_6.

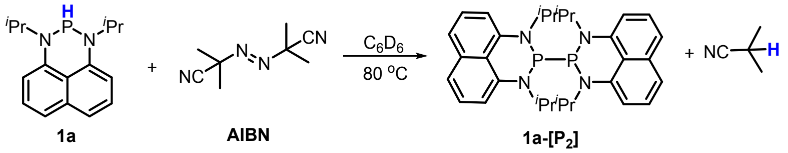

AIBN (0.03 mmol, 1.5 eq.) was added into the C_6D_6 (0.5 mL) solution of **1a** (0.02 mmol), and the mixture was heated at 80 °C for 3 hours. The reaction was monitored by NMR spectrum.

(1) ^{1}H NMR in C_6D_6

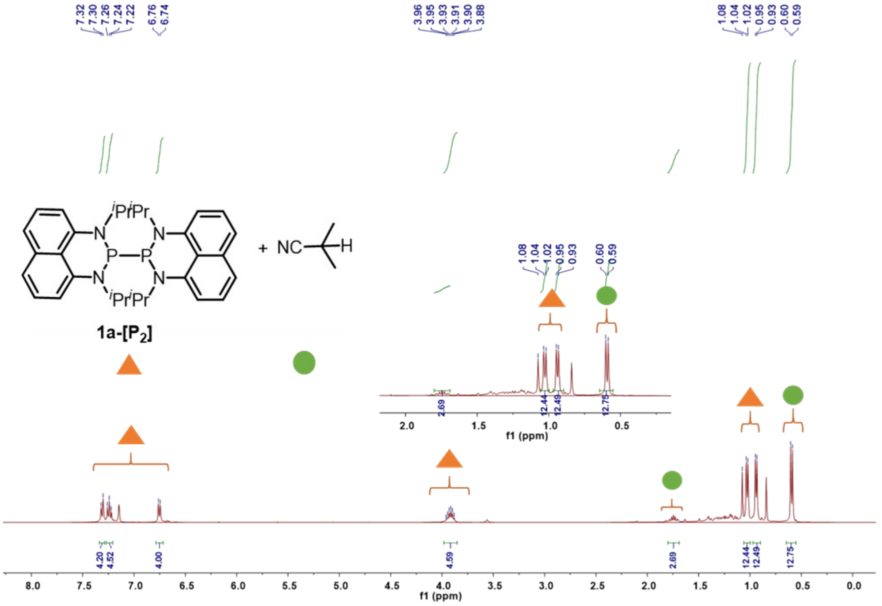

(2) ^{31}P NMR in C_6D_6

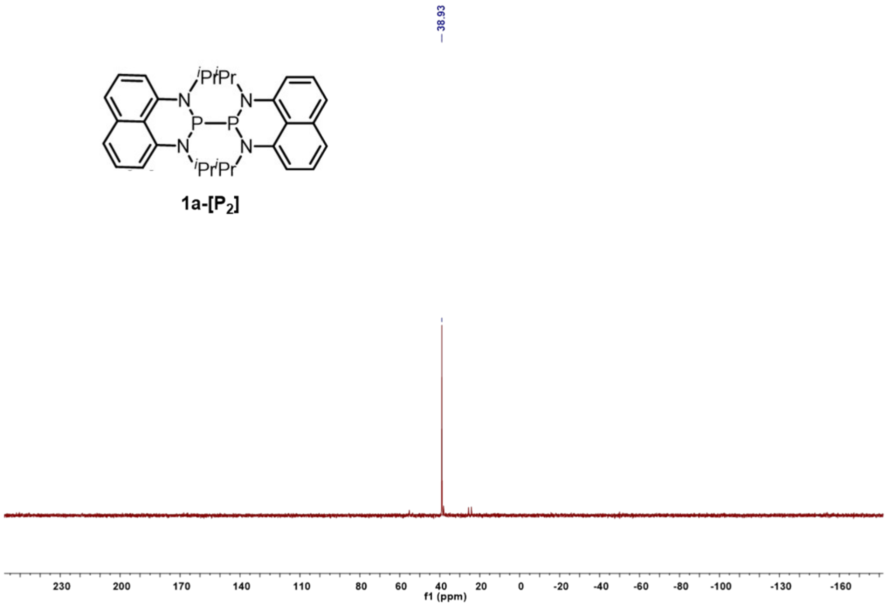

Figure S16. ^{1}H and ^{31}P NMR spectra comparison for the reaction between **1a** and AIBN. The integrations of ^{1}H NMR spectrum are assigned to the structure shown in the spectra and the byproduct $(CNC(CH_3)_2)_2$ was not marked.

The synthesis of 1a-[P₂] using the reaction of 1a with AIBN.

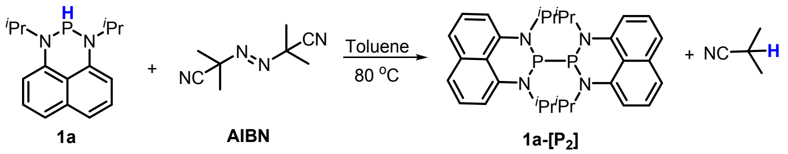

AIBN (0.3 mmol, 1.5 eq.) was added into the toluene (1.0 mL) solution of **1a** (0.2 mmol), and the mixture was stirred at 80 °C for about 3 hours. The solvent was rotary-evaporated, and the residue was washed with pentane (5 mL) and then filtered, producing **1a-[P₂]** as a yellow solid (50 mg, 92%).

^{1}H NMR (400 MHz, C_6D_6) δ 7.33 (d, J = 8.0 Hz, 4H), 7.26 (t, J = 7.8 Hz, 4H), 6.77 (d, J = 7.6 Hz, 4H), 3.99 - 3.88 (m, 4H), 1.04 (d, J = 6.7 Hz, 12H), 0.95 (d, J = 6.4 Hz, 12H). **^{31}P NMR** (162 MHz, C_6D_6) δ 38.77. **^{13}C NMR** (101 MHz, C_6D_6) δ 143.77, 136.50, 125.41, 122.99 (t, J = 4.1 Hz), 120.05, 110.70, 52.21 (t, J = 12.1 Hz), 23.22 (t, J = 9.6 Hz), 22.62 (t, J = 5.6 Hz).

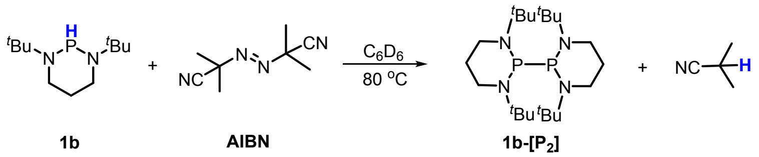

AIBN (0.03 mmol, 1.5 eq.) was added into the C_6D_6 (0.5 mL) solution of **1b (**0.02 mmol**)**, and the mixture was heated at 80 °C for 3 hours. The reaction was monitored by NMR spectrum.

The NMR spectroscopic data of **1b-[P₂]** are in good agreement with those reported in the literature.[12]

(1) ^{1}H NMR in C_6D_6

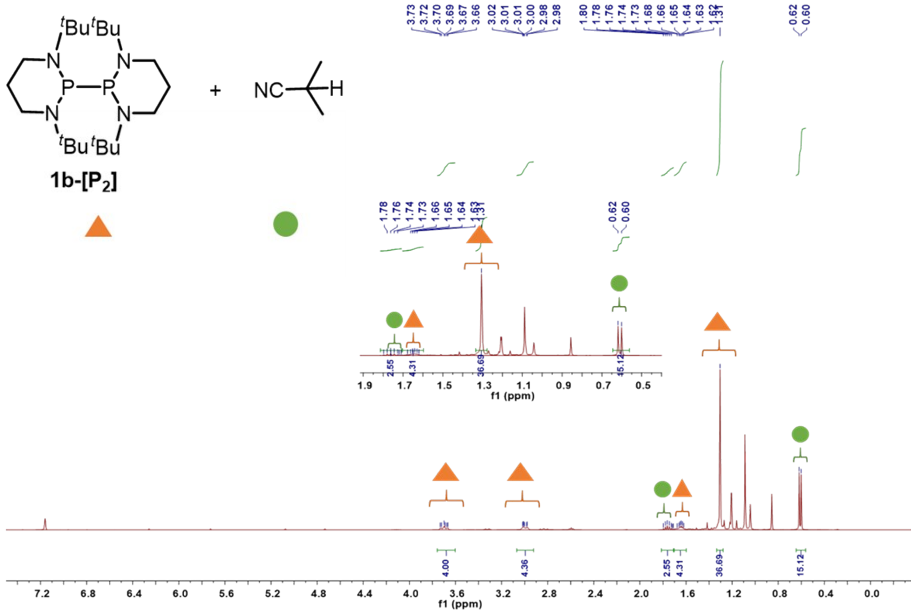

(2) ^{31}P NMR in C_6D_6

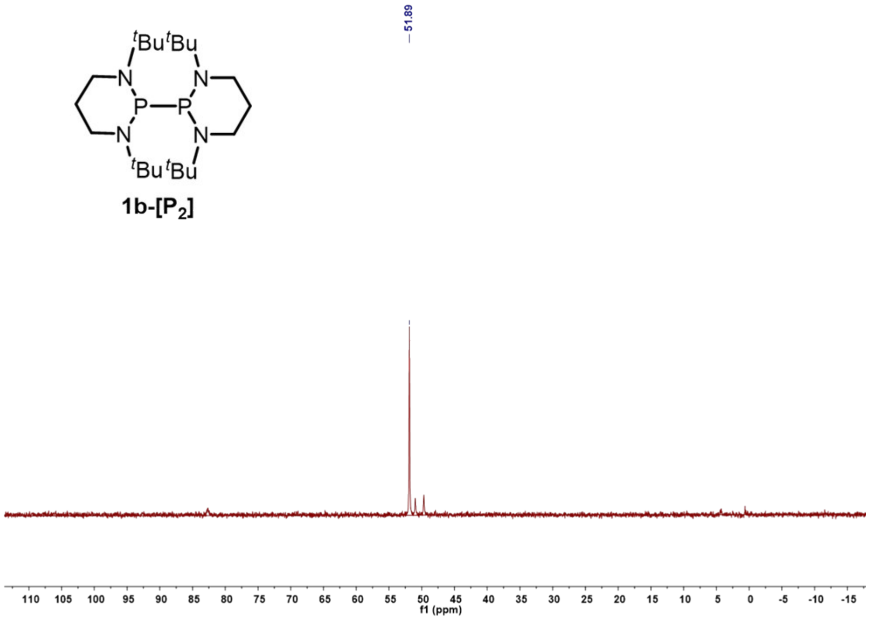

Figure S17. ^{1}H and ^{31}P NMR spectra for the reaction between **1b** and AIBN. The integrations of ^{1}H NMR spectrum are assigned to the structure shown in the spectrum and the byproduct $(CNC(CH_3)_2)_2$ was not marked.

18. The hydrodehalogenation reaction of bromobenzene.

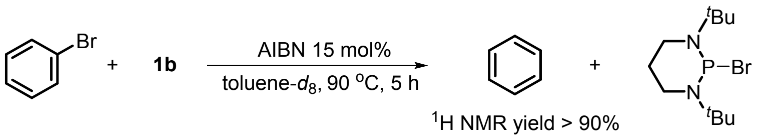

Bromobenzene (0.1 mmol), AIBN (15 mol%), **1b** (0.15 mmol) and toluene-d_8 (0.5 mL) were mixed in a Schlenk tube under argon and stirred at 90 °C for 5 hours. The ^{1}H NMR yield was given using 1,3,5-trimethoxybenzene (0.11 mmol) as internal standard.

(1) ^1H NMR of the mixture in toluene-d_8

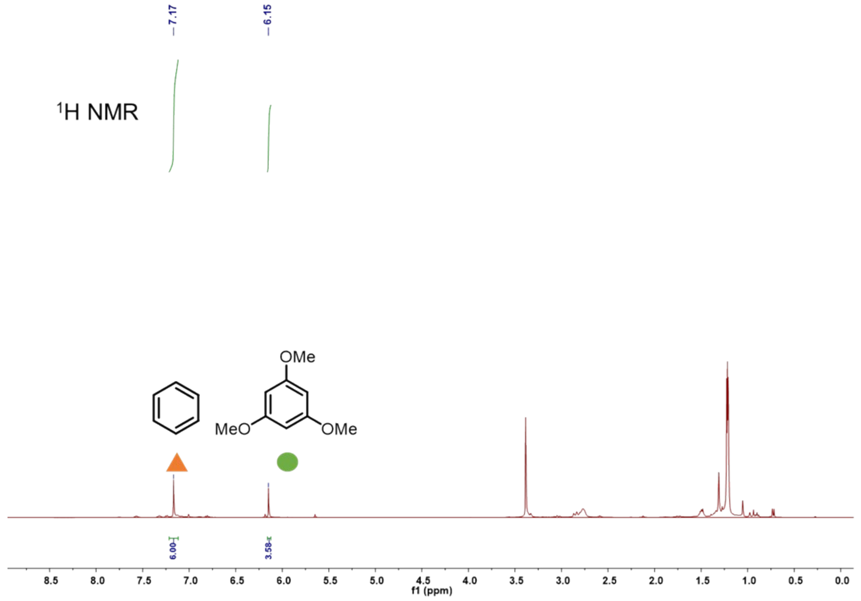

(2) ^{31}P NMR of the mixture in toluene-d_8

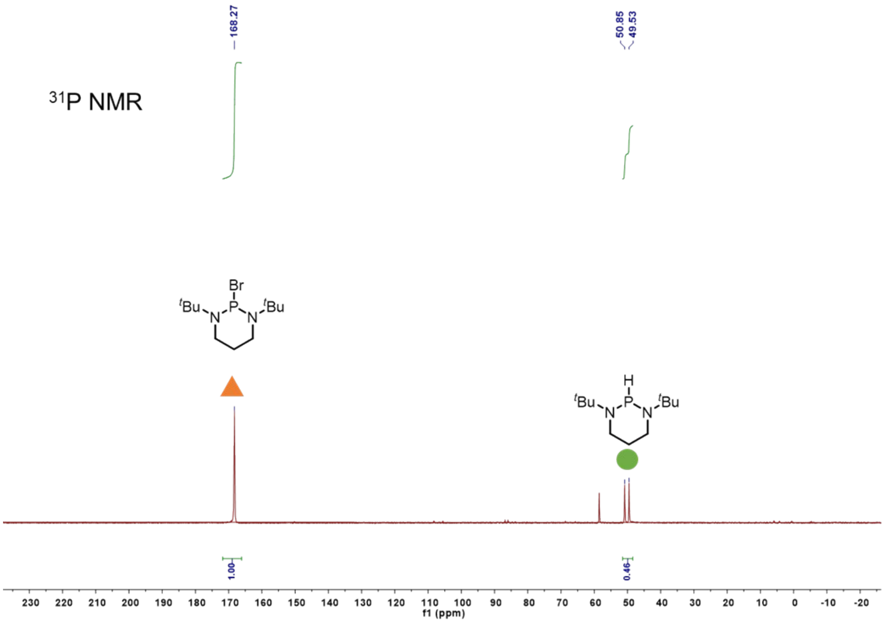

Figure S18. The NMR spectra of the mixture solution of hydrodehalogenation reaction of bromobenzene.

19. DFT Calculations.

Quantum calculations were conducted by using Gaussian 09[13]. Geometry optimizations and frequency computations were performed using the M06-2X[14] density functional in conjunction with the 6-31+G(d) basis set and an ultrafine integration grid. The SMD[15] model was used to account for the solvation effects of toluene, the solvent used experimentally. All of the optimized geometries were characterized as minima structures by frequency calculations. Thermal free energy corrections were obtained at 293.15 K. To obtain more accurate electronic energies, single-point energy calculations were performed at the (SMD)-M06-2X/6-311++G(2df,2p) level of

theory with the (SMD)-M06-2X/6-31+G(d) optimized structure.

The difference of bond dissociation free energies of P-Br bonds of **1a-Br** and **1b-Br** was calculated on the basis of reaction Gibbs free energy changes of Eq. S1 and S2 through DFT calculations. The result showed **1a-[P]•** and **1b-[P]•** should have a comparable ability (with an energy difference of 1.3 kcal/mol) in abstracting bromine atom. This failed to explain the disparate yields of <10% for **1a-[P]•** and 90% for **1b-[P]•**. Hence, the bromine abstraction pathway seems unlikely.

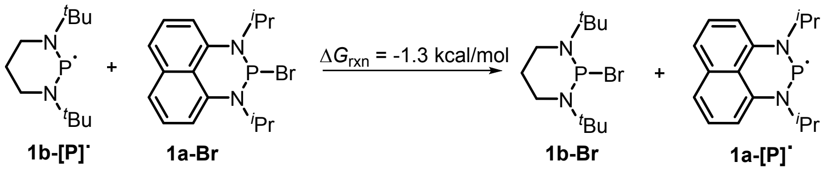

(S1)

$$\Delta G_{rxn} = \mathrm{BDFE}_{\mathbf{1b\text{-}Br}}(\text{P-Br}) - \mathrm{BDFE}_{\mathbf{1a\text{-}Br}}(\text{P-Br}) = 1.3\ \text{kcal/mol} \quad \text{(S2)}$$

20. NMR spectra.

^{1}H NMR

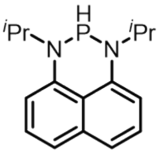

1a

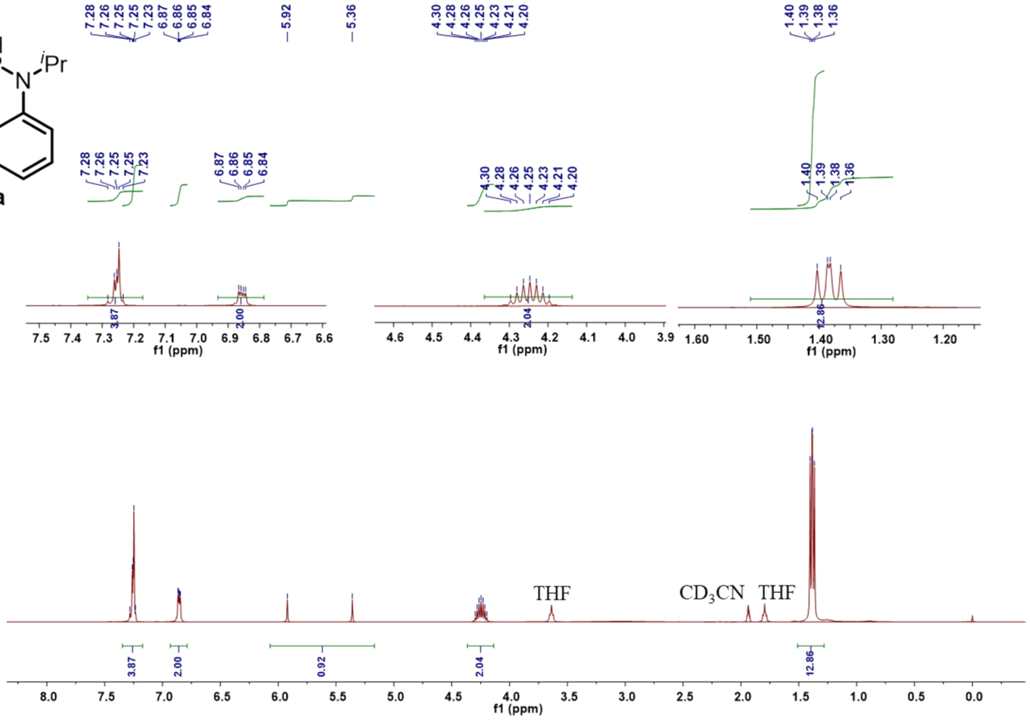

^{13}C NMR

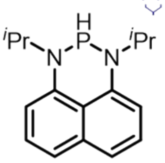

1a

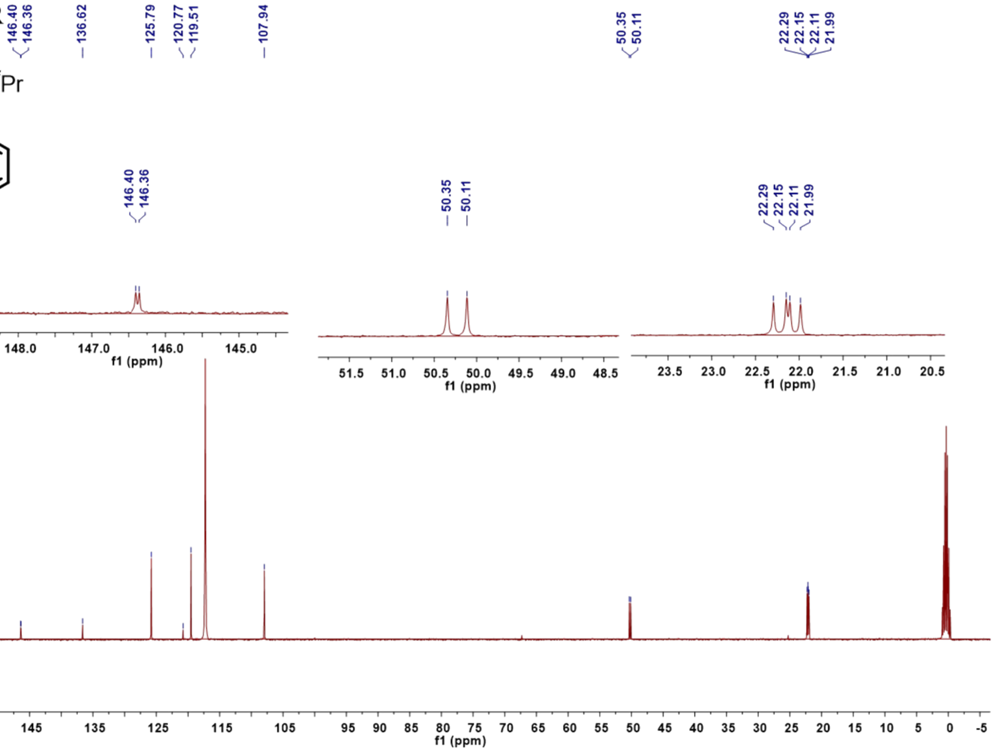

^{31}P NMR

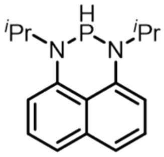

1a

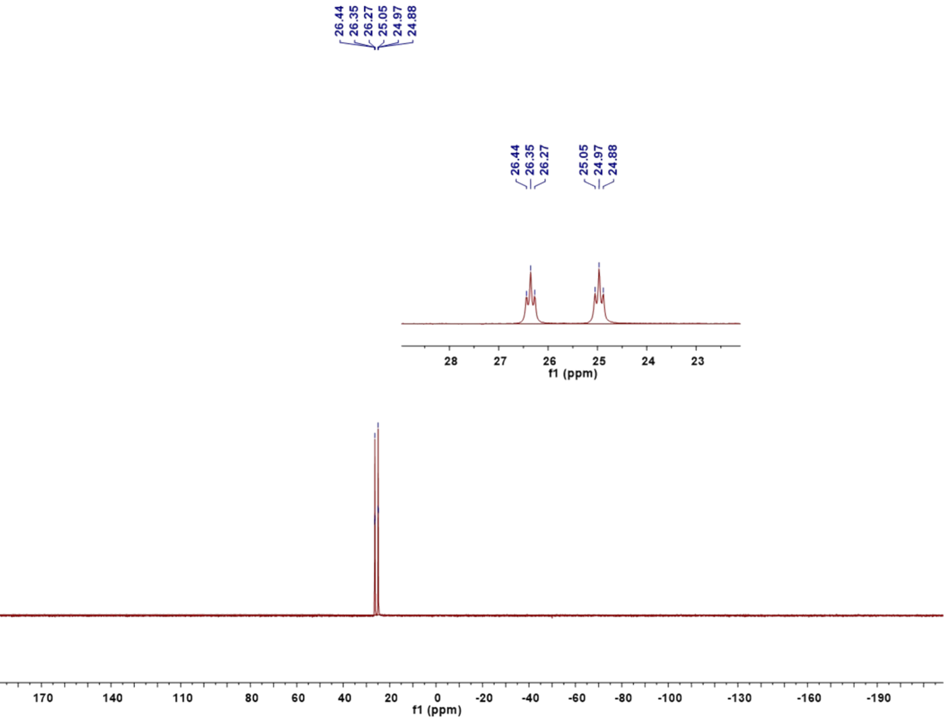

^{1}H NMR

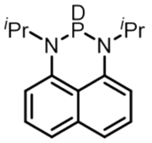

1a-D

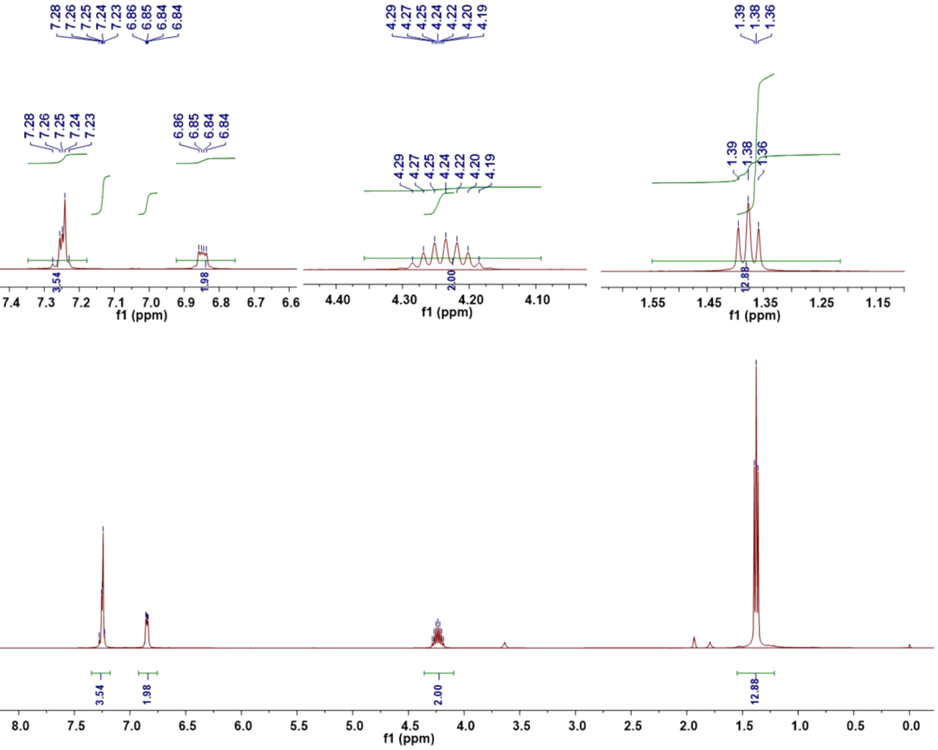

^{31}P NMR

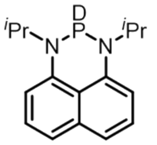

1a-D

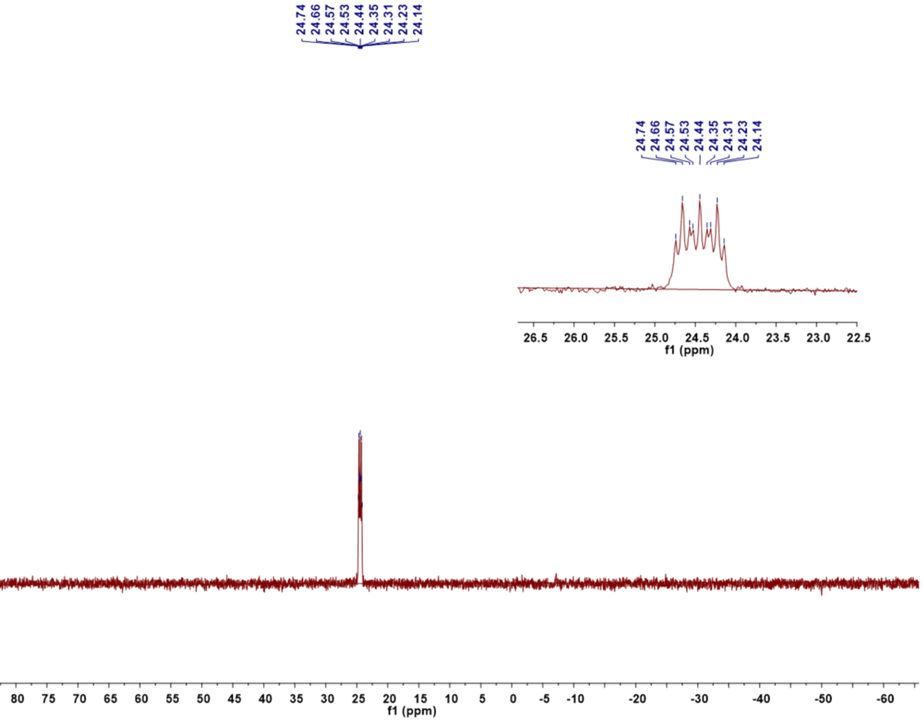

^{1}H NMR

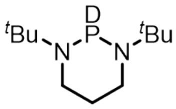

1b-D

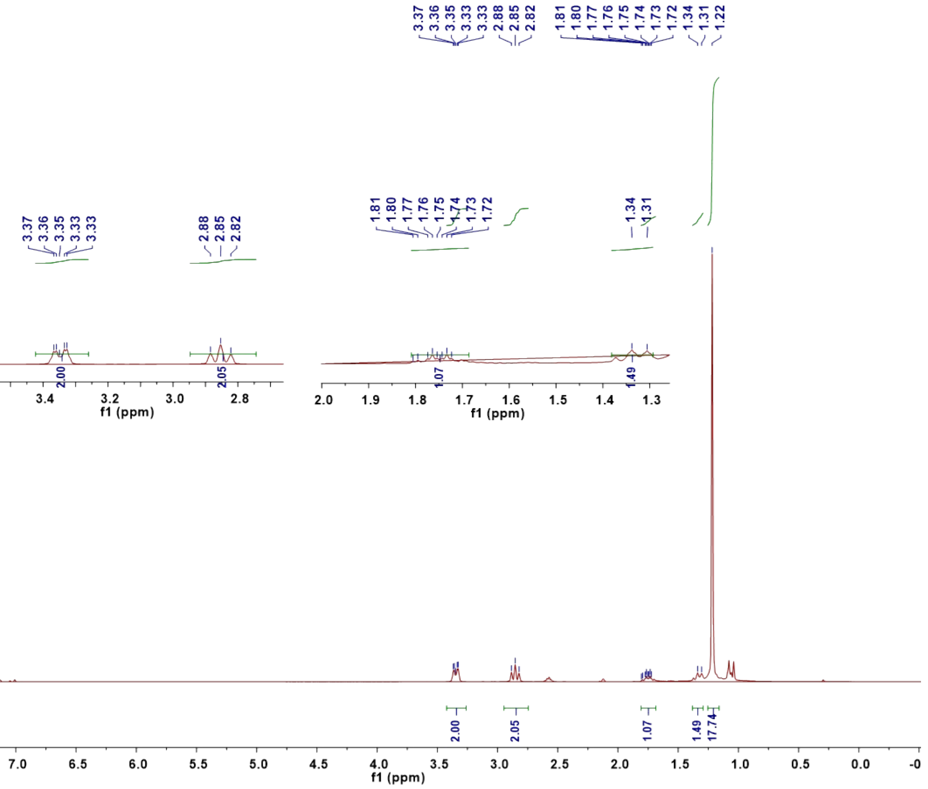

^{31}P NMR

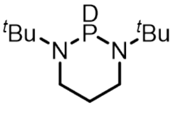

1b-D

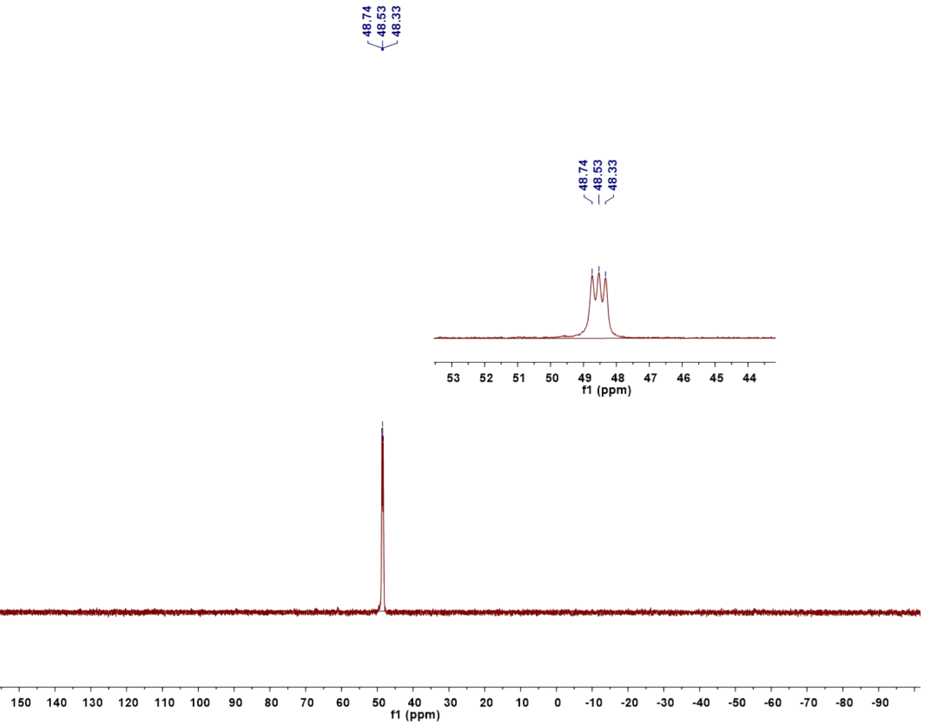

^{1}H NMR in CD_3CN

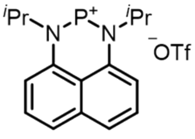

1a-[P]$^+$

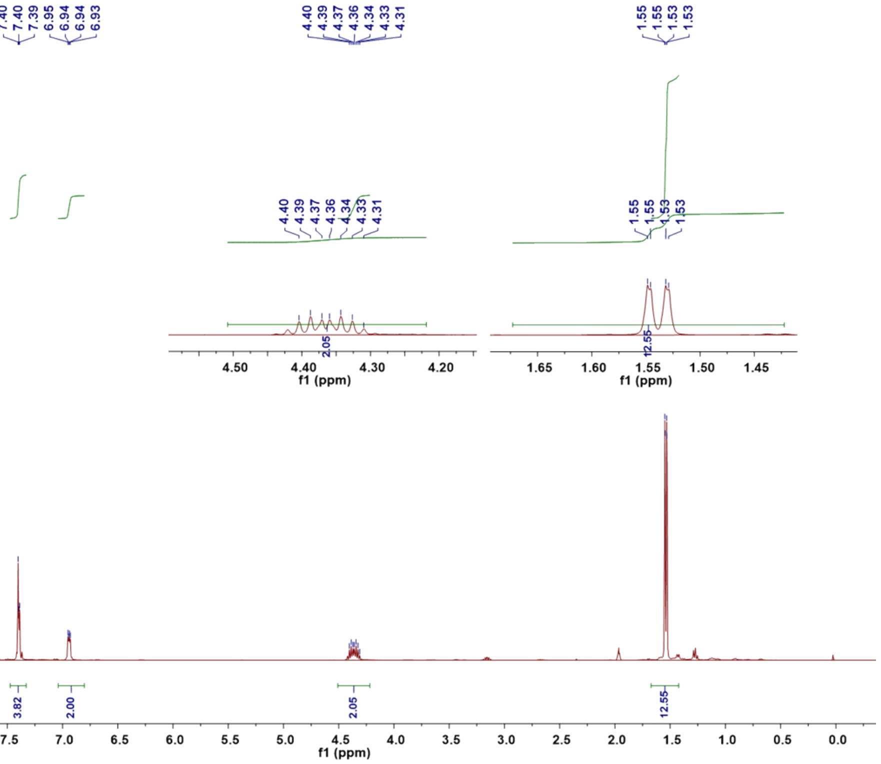

^{31}P NMR in CD_3CN

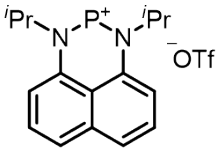

1a-[P]$^+$

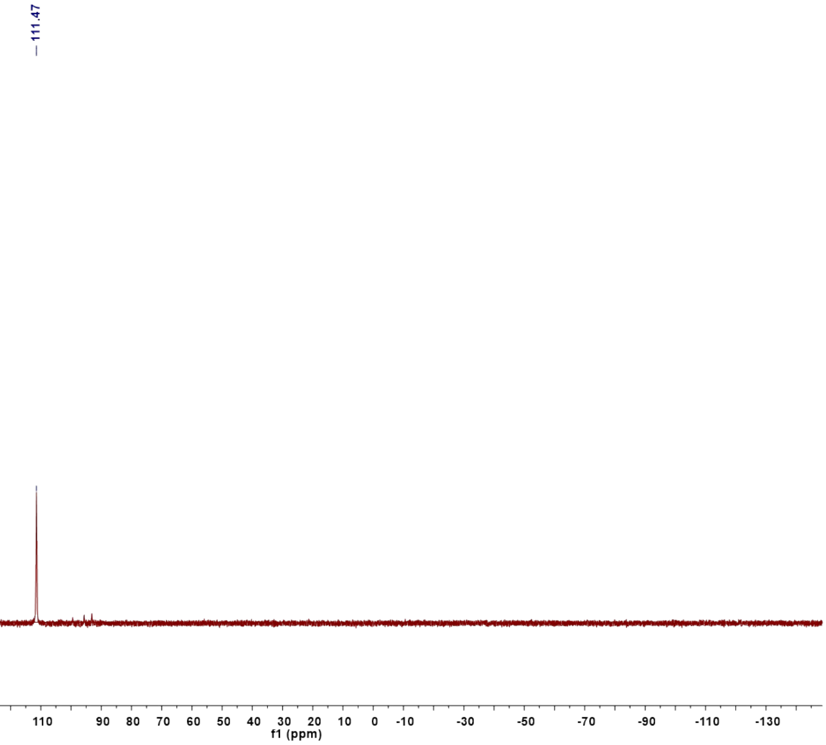

^{13}C NMR in CD_3CN

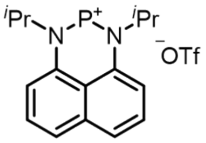

1a-[P]$^+$

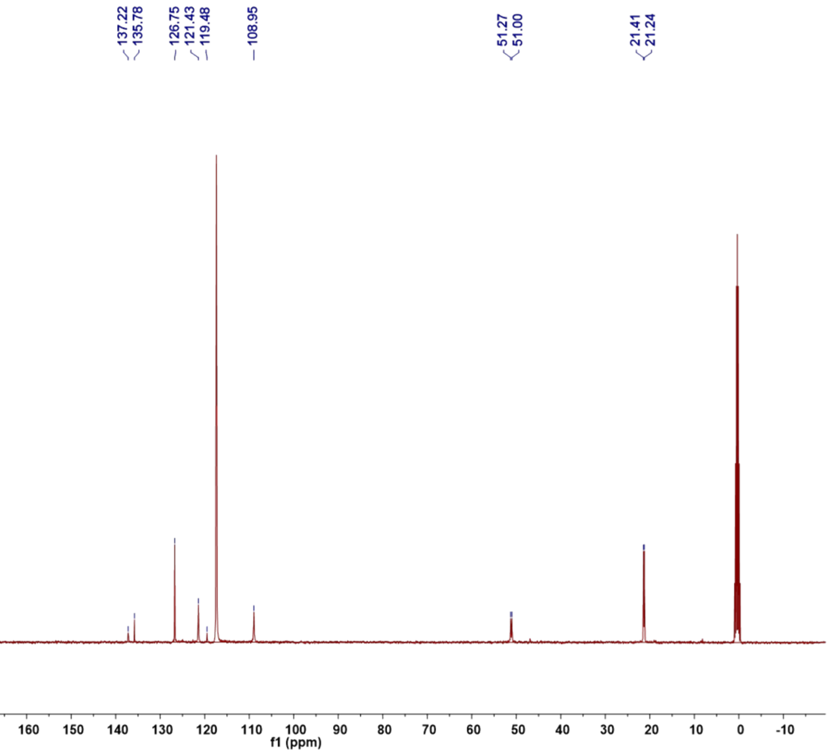

^{1}H NMR in toluene-d_8

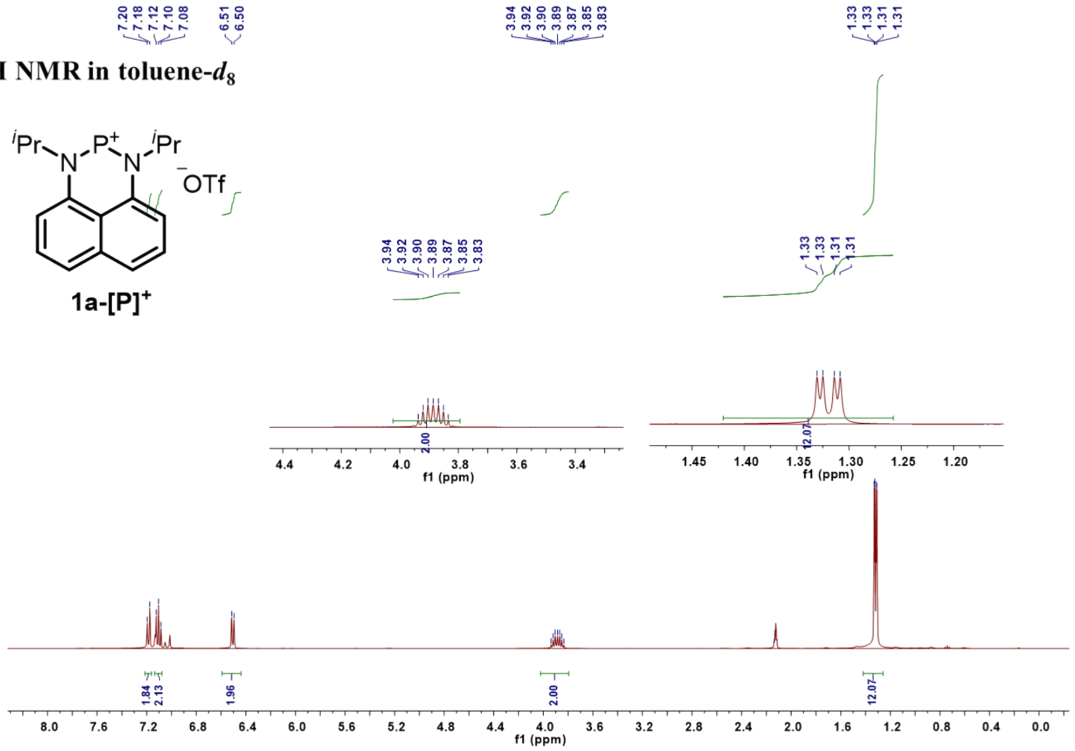

^{31}P NMR in toluene-d_8

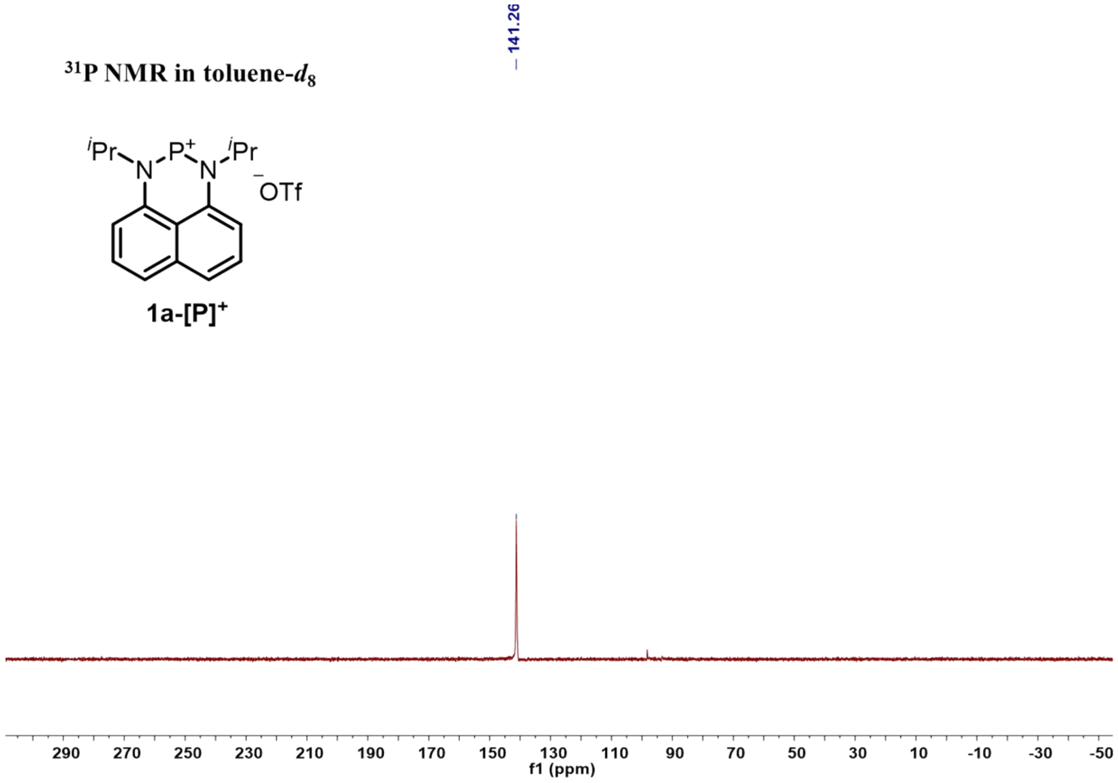

^{1}H NMR in CD_3CN

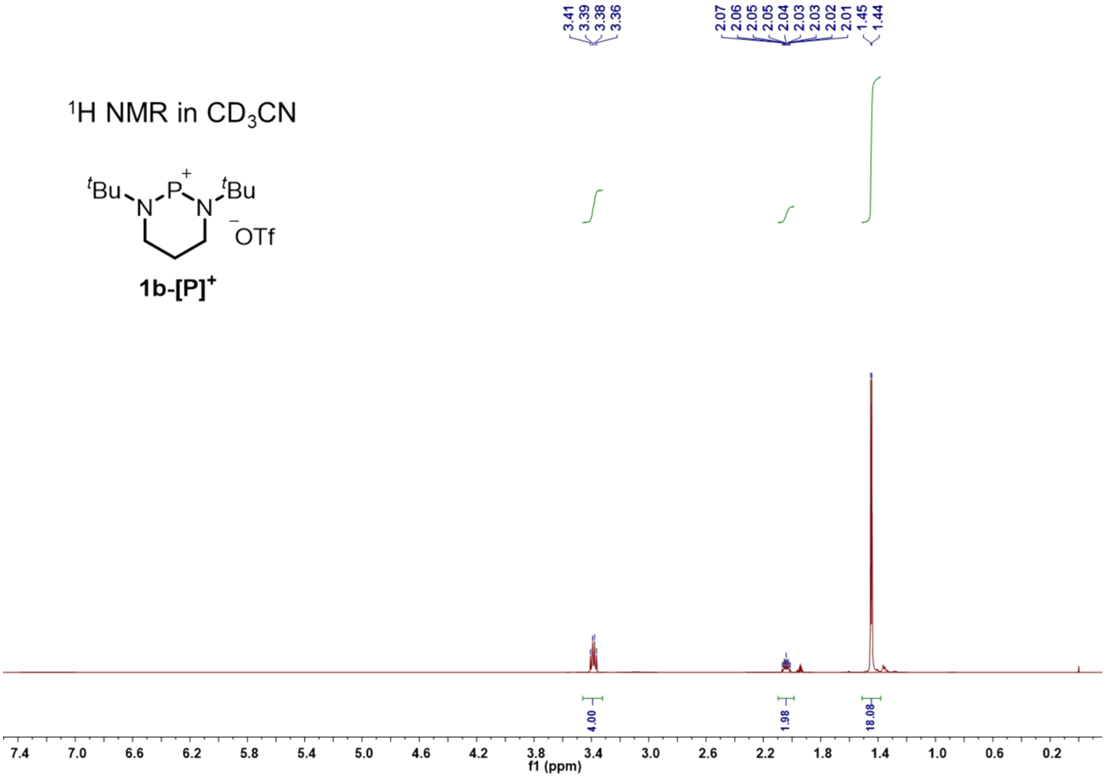

1b-[P]+

^{13}C NMR in CD_3CN

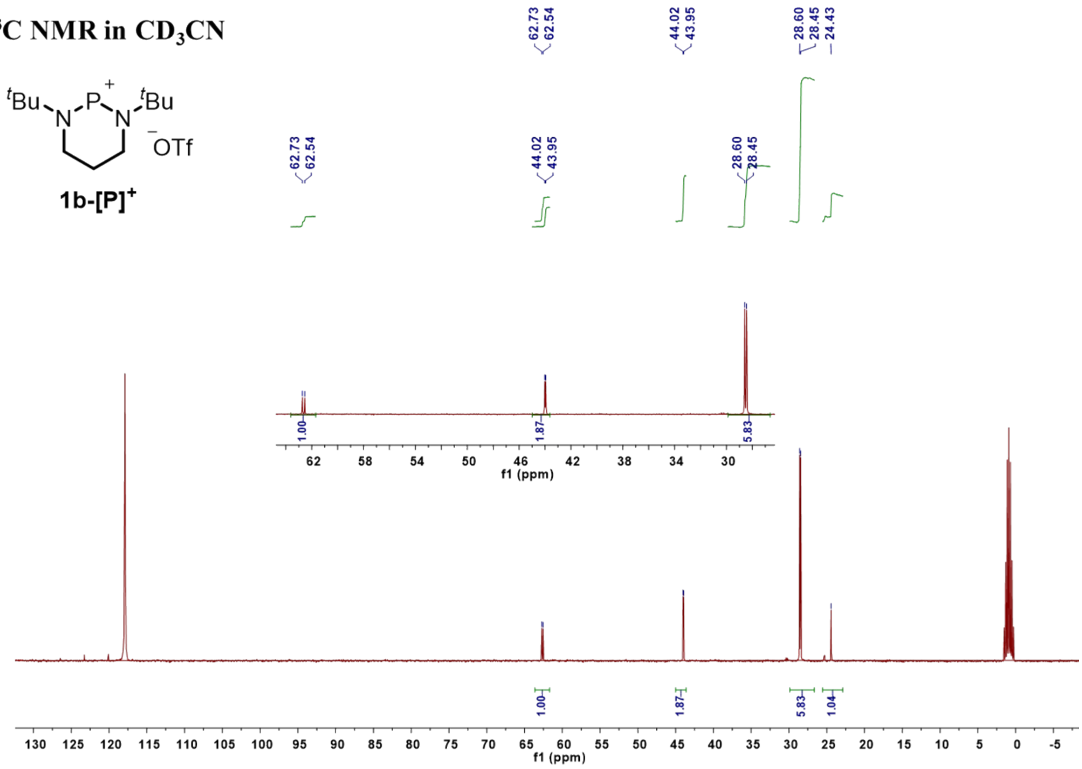

1b-[P]+

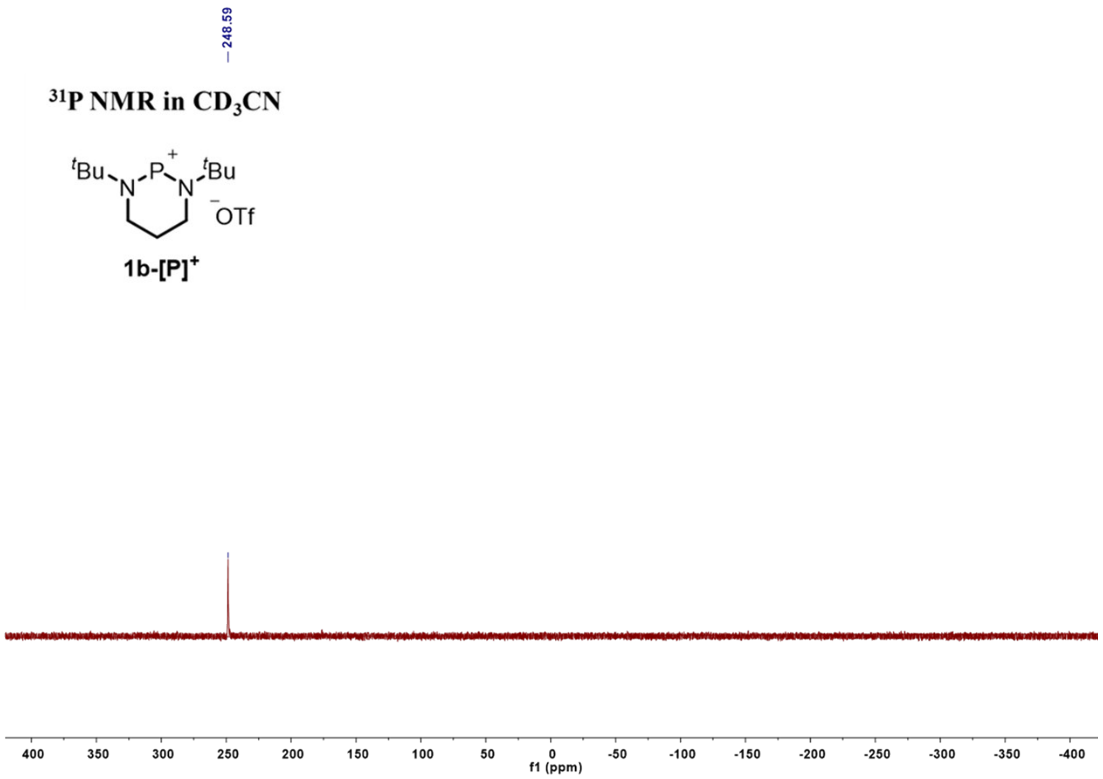

248.59
31P NMR in CD3CN
tBu
P
+
N
tBu
N
OTf
1b-[P]+
400 350 300 250 200 150 100 50 0 -50 -100 -150 -200 -250 -300 -350 -400
f1 (ppm)

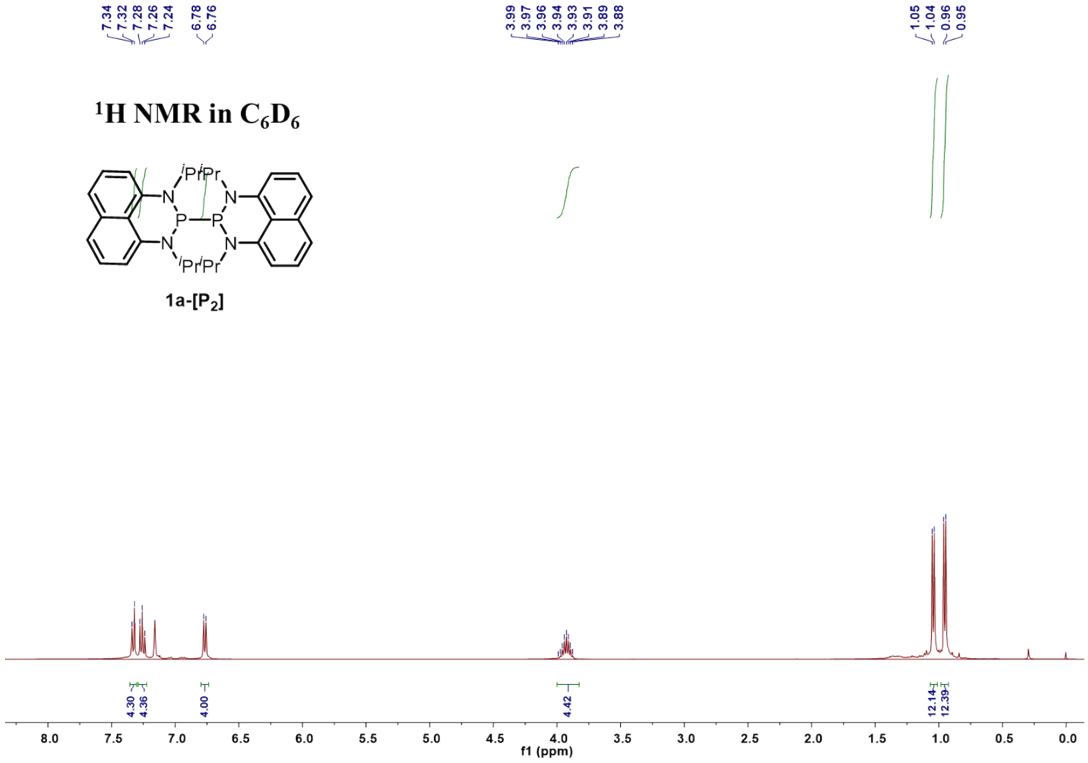

7.34 7.32 7.28 7.26 7.24 6.78 6.76
3.99 3.97 3.96 3.94 3.93 3.91 3.89 3.88
1.05 1.04 0.96 0.95
1H NMR in C6D6
iPr iPr
N
P
P
N
1a-[P2]
4.30 4.36 4.00 4.42 12.14 12.39
8.0 7.5 7.0 6.5 6.0 5.5 5.0 4.5 4.0 3.5 3.0 2.5 2.0 1.5 1.0 0.5 0.0
f1 (ppm)

^{31}P NMR in C_6D_6

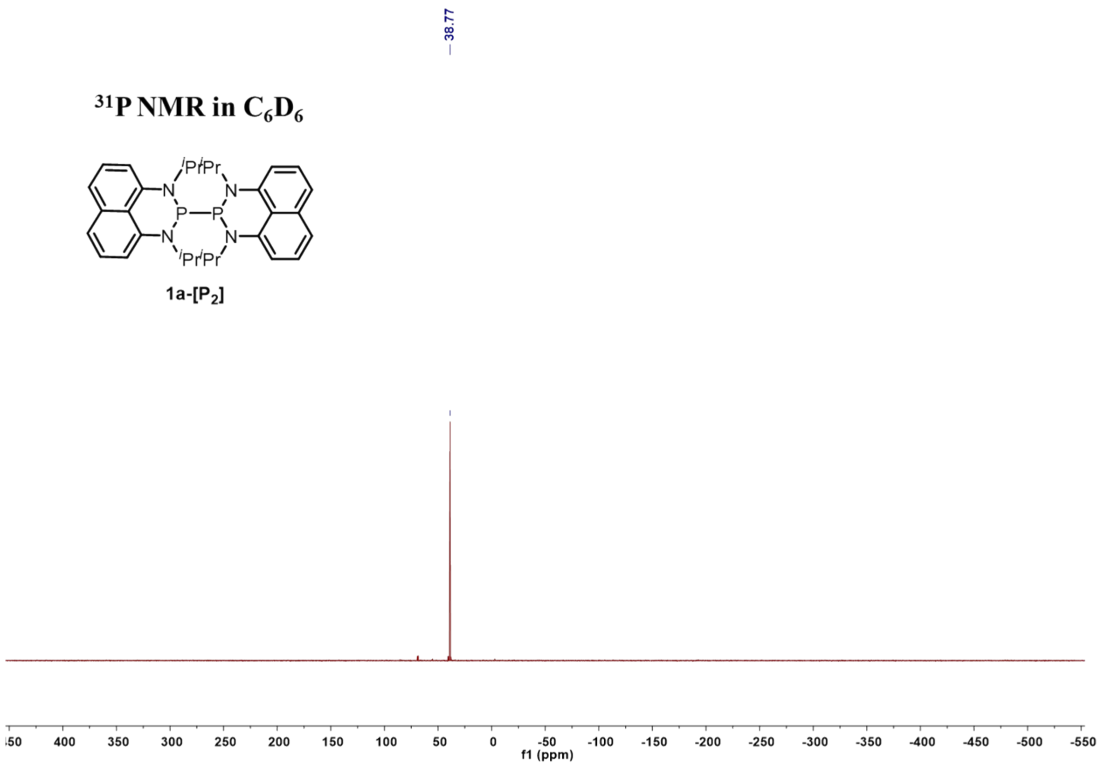

^{13}C NMR in C_6D_6

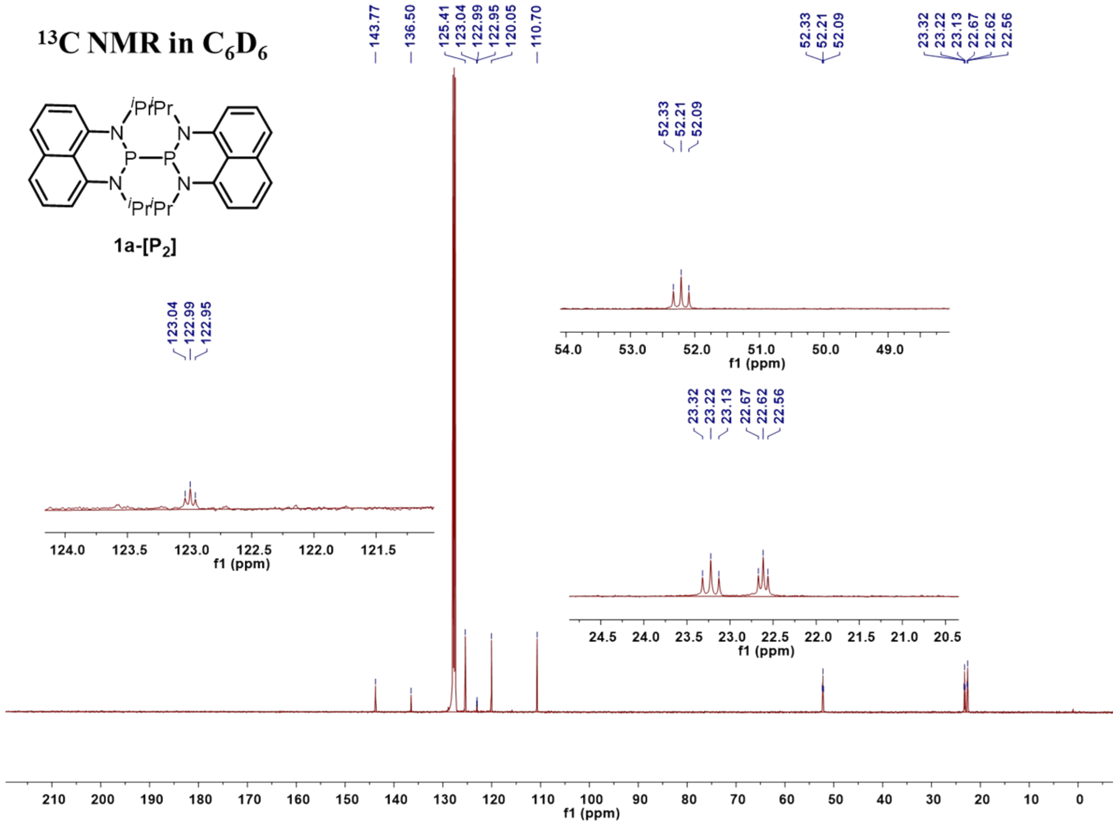

21. Crystal data of 1a.

The crystal structure of **1a** could be obtained by the volatilization of solution of **1a** in hexane at -30 °C. Crystallographic data of **1a** structure including CIF file have been deposited with the Cambridge Crystallographic Data Centre with the numbers of 1947293. Copies of these data can be requested from, free of charge, the CCDC website at https://www.ccdc.cam.ac.uk/structures/.

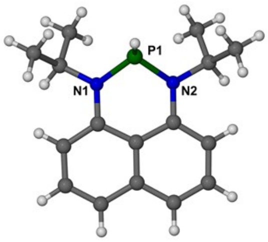


```
Bond precision:  = 0.0000 A              Wavelength=1.54184

Cell:            a=8.6130(19)     b=17.700(8)      c=9.511(2)
                 alpha=90         beta=90          gamma=90
Temperature:     173 K

                 Calculated                 Reported
Volume           1450.0(8)                  1449.9(8)
Space group      P n m a                    P n m a
Hall group       -P 2ac 2n                  -P 2ac 2n
                 0.9(C16 H21 N2 P), 2(C0.70 C14.4 H18.9 N1.8 P0.9,
Moiety formula   H0.75 N0.10 O0.05 P0.05),  2(C0.8 H1.05 N0.1 O0.05
                 0.2(C H3)                  P0.05)
Sum formula      C16 H21 N2 O0.10 P         C16 H21 N2 O0.10 P
Mr               273.92                     273.92
Dx,g cm-3        1.255                      1.255
Z                4                          4
Mu (mm-1)        1.575                      1.575
F000             587.2                      587.0
F000'            589.71
h,k,lmax         9,19,10                    9,19,10
Nref             1119                       1119
Tmin,Tmax        0.828,0.924                0.591,1.000
Tmin'            0.790

Correction method= # Reported T Limits: Tmin=0.591 Tmax=1.000
AbsCorr = MULTI-SCAN

Data completeness= 1.000          Theta(max)= 59.971

R(reflections)= 0.1192( 887)      wR2(reflections)= 0.3238( 1119)

S = 1.152                Npar= 134
```

22. SMD-M06-2X/6-31+G(d) calculated cartesian coordinates and energies.

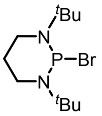

C	0.13815500	-0.82334900	2.18607300
C	-1.27395300	-0.63252400	1.62621300
H	-1.92284300	-1.43537700	1.99123300
H	-1.67798500	0.32196700	1.98567900
H	0.07128900	-1.32542800	3.15699500
C	0.99734600	-1.64084400	1.23221200
H	0.50975600	-2.59882200	1.00468300
H	1.95865400	-1.86321200	1.69771900
H	0.62306200	0.14505100	2.34559300
P	-0.02938900	-0.08509800	-0.75494100
N	1.22885000	-0.90816700	-0.01801900
N	-1.31829000	-0.65755200	0.15327900
C	-2.69504400	-0.62222600	-0.43601700
C	-3.33913300	-1.99889400	-0.23104500
C	-3.54689300	0.46954900	0.22623300

C	-2.64429300	-0.33951100	-1.94087500
H	-2.75164400	-2.77318100	-0.73618300
H	-3.42229500	-2.26371700	0.82731800
H	-4.35178900	-2.00263900	-0.64946200
H	-3.03577400	1.43767500	0.18293300
H	-4.50205500	0.55806600	-0.30238400
H	-3.77400600	0.24039300	1.27194900
H	-3.66203700	-0.40341700	-2.33868300
H	-2.26366400	0.66488600	-2.15713500
H	-2.02925600	-1.07035200	-2.47703500
C	2.63966500	-0.64916600	-0.42003100
C	2.68726700	0.09524800	-1.75920300
C	3.34176700	0.20709100	0.64313000
C	3.35522500	-1.99381700	-0.59576600
H	2.18294700	-0.46535000	-2.55359200
H	2.24760000	1.09620600	-1.69472100
H	3.73594300	0.21817900	-2.04918900
H	3.37533700	-0.29411700	1.61650900
H	4.37663100	0.40348700	0.34035600
H	2.82652400	1.16516000	0.76200400
H	4.38796400	-1.82516500	-0.91962200
H	3.39525600	-2.57179900	0.33272900
H	2.84816600	-2.59815700	-1.35594600
Br	0.11315700	2.20133800	0.19742900

Zero-point correction= 0.349784 (Hartree/Particle)

Thermal correction to Energy= 0.368050

Thermal correction to Enthalpy= 0.368994

Thermal correction to Gibbs Free Energy= 0.304532

Sum of electronic and zero-point Energies= -3455.717547

Sum of electronic and thermal Energies= -3455.699282

Sum of electronic and thermal Enthalpies= -3455.698338

Sum of electronic and thermal Free Energies= -3455.762799

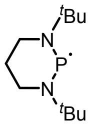

C	-0.16571600	2.17598100	0.37143100
C	1.26903400	1.66958100	0.24320100
H	1.75889000	2.22174500	-0.56696600
H	1.82293600	1.88199200	1.17255500
H	-0.17388200	3.26044400	0.21644100
C	-1.06969400	1.48343700	-0.64697500
H	-0.61711100	1.54868400	-1.64747100

H	-2.03292900	1.99594100	-0.69307500
H	-0.56186600	1.98193900	1.37562300
P	0.01527400	-0.77561000	0.44613700
N	-1.28622700	0.07923000	-0.29164800
N	1.30911800	0.23848600	-0.09014100
C	2.66635100	-0.37546000	-0.10623000
C	3.64467200	0.56401600	-0.82335600
C	3.17567400	-0.64581300	1.31863000
C	2.62706300	-1.69038300	-0.89481000
H	3.27228100	0.82694600	-1.82034400
H	3.82970800	1.48604400	-0.26348100
H	4.60785200	0.05696600	-0.94170400
H	2.53510200	-1.37274500	1.83147600
H	4.19451000	-1.05000900	1.29453800
H	3.19315300	0.27255500	1.91628500
H	3.64515000	-2.07617100	-1.01732500
H	2.03859700	-2.46140900	-0.38981400
H	2.19278300	-1.52717000	-1.88733800
C	-2.68135600	-0.35637700	-0.04606700
C	-2.72075400	-1.86177200	0.24094400
C	-3.28704500	0.39190500	1.15187700
C	-3.51385900	-0.10375200	-1.31123500
H	-2.24903600	-2.43045500	-0.56759700
H	-2.22459400	-2.11926100	1.18268000
H	-3.76488800	-2.18169300	0.32187900
H	-3.31235500	1.47481000	0.98578400
H	-4.31709100	0.06201200	1.33111700
H	-2.70063800	0.19598500	2.05705600
H	-4.53134200	-0.48559000	-1.17120000
H	-3.59574600	0.95966600	-1.55590500
H	-3.06505900	-0.61927700	-2.16718800

Zero-point correction= 0.346785 (Hartree/Particle)

Thermal correction to Energy= 0.363505

Thermal correction to Enthalpy= 0.364449

Thermal correction to Gibbs Free Energy= 0.303085

Sum of electronic and zero-point Energies= -883.837231

Sum of electronic and thermal Energies= -883.820511

Sum of electronic and thermal Enthalpies= -883.819567

Sum of electronic and thermal Free Energies= -883.880931

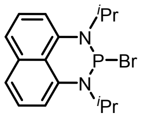

C	2.99007800	2.41605600	-0.17082000

C	3.66165300	1.22760300	-0.07714000
C	2.95386800	0.00010400	-0.16339100
C	1.54409100	0.00005100	-0.38478700
C	0.86806500	1.26609400	-0.48653500
C	1.59261000	2.43865900	-0.35783300
H	4.73739100	-1.19964600	0.07426500
H	3.52295800	3.35925800	-0.08825400
H	4.73729400	1.19998900	0.07430300
C	3.66175100	-1.22734100	-0.07717900
C	0.86815800	-1.26604100	-0.48656300
H	1.09825900	3.40124800	-0.38828000
C	1.59280100	-2.43855200	-0.35789500
C	2.99026800	-2.41584200	-0.17088700
H	1.09853300	-3.40118200	-0.38835200
H	3.52322300	-3.35900500	-0.08834700
N	-0.52240000	1.28187100	-0.71888200
N	-0.52231400	-1.28190900	-0.71889300
P	-1.49521300	-0.00005400	-0.19651400
C	-1.21012600	2.57020200	-0.97796400
C	-1.59289300	3.29541500	0.31251500
C	-2.42041700	2.37869800	-1.89149200
H	-0.48779300	3.16850100	-1.53950500
H	-0.75117700	3.36228600	1.00809600
H	-1.94101000	4.30963700	0.08777500
H	-2.40645400	2.76618100	0.82285200
H	-2.15168100	1.79536300	-2.77831900
H	-3.24978200	1.87839100	-1.38195200
H	-2.77797500	3.36010800	-2.21977500
C	-1.20997200	-2.57027200	-0.97799300
C	-2.42037100	-2.37878200	-1.89137600
C	-1.59255800	-3.29559500	0.31247800
H	-0.48766500	-3.16848600	-1.53965700
H	-2.15176700	-1.79536800	-2.77819200
H	-2.77790200	-3.36019200	-2.21968800
H	-3.24971300	-1.87856100	-1.38171100
H	-0.75076100	-3.36246500	1.00796000
H	-2.40609300	-2.76644100	0.82293900
H	-1.94064100	-4.30982100	0.08770700
Br	-1.00308800	-0.00004200	2.08284100

Zero-point correction= 0.333962 (Hartree/Particle)

Thermal correction to Energy= 0.353733

Thermal correction to Enthalpy= 0.354677

Thermal correction to Gibbs Free Energy= 0.285126

Sum of electronic and zero-point Energies= -3643.827442

Sum of electronic and thermal Energies= -3643.807671

Sum of electronic and thermal Enthalpies= -3643.806727

Sum of electronic and thermal Free Energies= -3643.876278

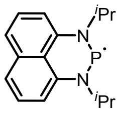

C	2.88137500	2.27275800	-0.08881800
C	3.49766900	1.05090900	-0.07218600
C	2.71999600	-0.13693100	-0.06054600
C	1.29293600	-0.06567000	-0.05911000
C	0.67201100	1.23715500	-0.08094500
C	1.47670300	2.36952800	-0.08887100
H	4.46162800	-1.41907400	-0.06776700
H	3.46897200	3.18678800	-0.09255800
H	4.58078700	0.96550600	-0.07012900
C	3.37532800	-1.39616400	-0.06943100
C	0.54515100	-1.30014500	-0.07951500
H	1.03878900	3.35847500	-0.07328400
C	1.23283500	-2.50702600	-0.08310600
C	2.64038000	-2.55051600	-0.08282000
H	0.69922300	-3.44796200	-0.06498200
H	3.13402900	-3.51866700	-0.08411600
N	-0.72621400	1.33880000	-0.10992100
N	-0.85635400	-1.26036200	-0.11263000
P	-1.73070800	0.08567600	0.50285100
C	-1.35525300	2.67125400	-0.24728900
C	-1.46008600	3.39294200	1.09823900
C	-2.71809700	2.58750100	-0.93430600
H	-0.70587100	3.23866300	-0.91995000
H	-0.49665100	3.43111300	1.61540800
H	-1.81855700	4.41875400	0.95768200
H	-2.17158000	2.87320400	1.75101200
H	-2.65937500	1.99728200	-1.85439400
H	-3.48549100	2.14840900	-0.28922500
H	-3.04325200	3.60040300	-1.19455500
C	-1.61703500	-2.52286300	-0.24814000
C	-2.95962400	-2.30323800	-0.94484500
C	-1.80210700	-3.22175800	1.10128400
H	-1.02509200	-3.15706200	-0.91390500
H	-2.83251200	-1.72775200	-1.86730400
H	-3.38673700	-3.27837300	-1.20212900
H	-3.68168100	-1.78291200	-0.30774500
H	-0.85015100	-3.34813600	1.62588700

H	-2.46431000	-2.63002000	1.74443900
H	-2.25666100	-4.20926500	0.96515000

Zero-point correction= 0.333028 (Hartree/Particle)

Thermal correction to Energy= 0.350802

Thermal correction to Enthalpy= 0.351746

Thermal correction to Gibbs Free Energy= 0.287505

Sum of electronic and zero-point Energies= -1071.950951

Sum of electronic and thermal Energies= -1071.933178

Sum of electronic and thermal Enthalpies= -1071.932233

Sum of electronic and thermal Free Energies= -1071.996475

23. Reference.

1. H. A. Spinney, I. Korobkov, G. A. DiLabio, G. P. A. Yap and D. S. Richeson, *Organometallics*, 2007, **26**, 4972-4982.
2. J. Zhang, J.-D. Yang and J.-P. Cheng, *Angew. Chem. Int. Ed.*, 2019, **58**, 5983-5987.
3. J. Eberhard, K. Peuntinger, R. Fröhlich, D. M. Guldi and J. Mattay, *Eur. J. Org. Chem.*, 2018, **2018**, 2682-2700.
4. A. Motaleb, A. Bera and P. Maity, *Org. Biomol. Chem.*, 2018, **16**, 5081-5085.
5. X.-Q. Zhu, M.-T. Zhang, A. Yu, C.-H. Wang and J.-P. Cheng, *J. Am. Chem. Soc.*, 2008, **130**, 2501-2516.
6. J. Auth, J. Padevet, P. Mauleón and A. Pfaltz, *Angew. Chem. Int. Ed.*, 2015, **54**, 9542-9545.
7. J. J. Warren, T. A. Tronic and J. M. Mayer, *Chem. Rev.*, 2010, **110**, 6961-7001.
8. Internet Bond-energy Databank (iBonD) Home Page: http://ibond.nankai.edu.cn, accessed at Oct. 2019.
9. H. A. Spinney, G. P. A. Yap, I. Korobkov, G. DiLabio and D. S. Richeson, *Organometallics*, 2006, **25**, 3541-3543.
10. S. Ilic, A. Alherz, C. B. Musgrave and K. D. Glusac, *Chem. Soc. Rev.*, 2018, **47**, 2809-2836.
11. (a) B. Rao, C. C. Chong and R. Kinjo, *J. Am. Chem. Soc.*, 2018, **140**, 652-656; (b) T. Hynes, E. N. Welsh, R. McDonald, M. J. Ferguson and A. W. H. Speed, *Organometallics*, 2018, **37**, 841-844.
12. E. E. Nifantiev, N. S. Vyazankin, S. F. Sorokina, L. A. Vorobieva, O. A. Vyazankina, D. A. Bravo-Zhivotovsky and A. R. Bekker, *J. Organomet. Chem.*, 1984, **277**, 211-225.
13. M. J. T. Frisch, G. W.; Schlegel, H. B.; Scuseria, G. E.;Robb, M. A.; Cheeseman, J. R.; Scalmani, G.; Barone, V.; Mennucci,B.; Petersson, G. A.; Nakatsuji, H.; Caricato, M.; Li, X.; Hratchian, H.P.; Izmaylov, A. F.; Bloino, J.; Zheng, G.; Sonnenberg, J. L.; Hada, M.;Ehara, M.; Toyota, K.; Fukuda, R.; Hasegawa, J.; Ishida, M.; Nakajima,T.; Honda, Y.; Kitao, O.; Nakai, H.; Vreven, T.; Montgomery, J. A., Jr.;J. E, P.; Ogliaro, F.; Bearpark, M.; Heyd, J. J.; Brothers, E.; Kudin, K.N.; Staroverov, V. N.; Keith, T.; Kobayashi, R.; Normand, J.;Raghavachari, K.; Rendell, A.; Burant, J. C.; Iyengar, S. S.; Tomasi,J.; Cossi, M.; Rega, N.; Millam, J. M.; Klene, M.; Knox, J. E.; Cross, J.B.; Bakken, V.; Adamo, C.; Jaramillo, J.; Gomperts, R.; Stratmann, R.E.; Yazyev, O.; Austin, A. J.; Cammi, R.; Pomelli, C.; Ochterski, J. W.;Martin, R. L.; Morokuma, K.; Zakrzewski, V. G.; Voth, G. A.; Salvador,P.; Dannenberg, J. J.; Dapprich, S.; Daniels, A. D.; Farkas, O.;Foresman, J. B.; Ortiz, J. V.; Cioslowski, J.; Fox, D. J.Gaussian 09,Revision D.01, Gaussian, Inc., Wallingford, CT, 2013.
14. (a) Y. Zhao and D. G. Truhlar, *Acc. Chem. Res.*, 2008, **41**, 157-167; (b) Y. Zhao and D. G. Truhlar, *Chem. Phys. Lett.*, 2011, **502**, 1-13.
15. A. V. Marenich, C. J. Cramer and D. G. Truhlar, *J. Phys. Chem. B*, 2009, **113**, 6378-6396.
